# Tox21Enricher-Shiny: an R Shiny application for toxicity functional annotation analysis

**DOI:** 10.3389/ftox.2023.1147608

**Published:** 2023-06-27

**Authors:** Parker Combs, Jeremy Erickson, Jui-Hua Hsieh, Kai Guo, Sue Nolte, Charles Schmitt, Scott Auerbach, Junguk Hur

**Affiliations:** ^1^ Department of Biomedical Sciences, School of Medicine and Health Sciences, University of North Dakota, Grand Forks, ND, United States; ^2^ National Institute of Environmental Health Sciences, Durham, NC, United States; ^3^ Michigan Medicine, University of Michigan Health, Ann Arbor, MI, United States

**Keywords:** toxicity analysis, Tox21, enrichment analysis, R shiny, web-based application

## Abstract

Inference of toxicological and mechanistic properties of untested chemicals through structural or biological similarity is a commonly employed approach for initial chemical characterization and hypothesis generation. We previously developed a web-based application, Tox21Enricher-Grails, on the Grails framework that identifies enriched biological/toxicological properties of chemical sets for the purpose of inferring properties of untested chemicals within the set. It was able to detect significantly overrepresented biological (e.g., receptor binding), toxicological (e.g., carcinogenicity), and chemical (e.g., toxicologically relevant chemical substructures) annotations within sets of chemicals screened in the Tox21 platform. Here, we present an R Shiny application version of Tox21Enricher-Grails, Tox21Enricher-Shiny, with more robust features and updated annotations. Tox21Enricher-Shiny allows users to interact with the web application component (available at http://hurlab.med.und.edu/Tox21Enricher/) through a user-friendly graphical user interface or to directly access the application’s functions through an application programming interface. This version now supports InChI strings as input in addition to CASRN and SMILES identifiers. Input chemicals that contain certain reactive functional groups (nitrile, aldehyde, epoxide, and isocyanate groups) may react with proteins in cell-based Tox21 assays: this could cause Tox21Enricher-Shiny to produce spurious enrichment analysis results. Therefore, this version of the application can now automatically detect and ignore such problematic chemicals in a user’s input. The application also offers new data visualizations, and the architecture has been greatly simplified to allow for simple deployment, version control, and porting. The application may be deployed onto a Posit Connect or Shiny server, and it uses Postgres for database management. As other Tox21-related tools are being migrated to the R Shiny platform, the development of Tox21Enricher-Shiny is a logical transition to use R’s strong data analysis and visualization capacities and to provide aesthetic and developmental consistency with other Tox21 applications developed by the Division of Translational Toxicology (DTT) at the National Institute of Environmental Health Sciences (NIEHS).

## 1 Introduction

Comprehensive toxicological characterization of chemicals is a time-consuming and costly undertaking, which has motivated scientists to explore alternative, more targeted and hypothesis-driven approaches to evaluate chemical toxicity (e.g., structure-activity relationship (SAR) analysis). These efforts have focused primarily on either chemical structure or high dimensional *in vitro* data (e.g., Tox21 and ToxCast) coupled with informatic/machine learning methods that leverage a variety of data streams to infer toxicological properties of untested chemicals based on those that do have data (e.g., SAR, read-across). Our tool, Tox21Enricher-Shiny, facilitates the latter approach by performing chemical set enrichment using an extensive collection of annotations associated with chemicals in the Tox21 10k library that describe chemical, biological, and toxicological properties.

We envision using Tox21Enricher-Shiny for hypothesis generation, i.e., a user can infer toxicological properties of untested chemicals through set-based enrichment of chemicals that contain a mix of tested and untested chemicals. Other tools like quantitative structure-activity relationship (QSAR) models and the Generalized Read-Across (GenRA) tool provide powerful and high-certainty read-across capabilities in which they can make more focused and specific predictions based on a more constrained information domain (Generalized Read-Across GenRA, 2023). In comparison, Tox21Enricher-Shiny is built using a broader, less focused landscape of annotations relating to toxicology, mechanistic and molecular pathways, chemistry, and pharmacological properties. Because of the diverse-but-general nature of its information domain, Tox21Enricher-Shiny is not appropriate for making very specific, focused predictions about chemicals; rather, the tool’s results should be used to support and provide additional context to the predictions derived from tools like GenRA. Therefore, during hypothesis generation, it is recommended to incorporate Tox21Enricher-Shiny alongside other read-across tools to supplement the predictions and establish a collective weight of evidence.

The user may prepare input sets for Tox21Enricher-Shiny by formulating lists of similar chemicals related by chemical structure similarity or biological similarity (e.g., measured or predicted patterns of bioactivity across high throughput assays). The application natively supports the lookup of structurally similar chemicals for a given chemical (either by finding chemicals with shared structures or by calculating similarity using Tanimoto coefficients), but biological similarity calculations must be done externally before using the application. Bearing in mind the underlying concept that structurally similar chemicals or those that exhibit similar patterns of bioactivity often demonstrate similar toxicological and mechanistic properties, using our tool to enrich chemical sets defined through chemical and/or biological similarity will inform on the overall properties of each chemical set. Therefore, Tox21Enricher-Shiny allows for the inference of a hypothetical set of biological/toxicological properties of the untested chemicals contained in the input through a process akin to nearest neighbor-based inference.

Tox21 Enricher-Shiny contains annotations from various sources for 8,948 chemicals derived from the Tox21 10K library, which is a set of 10,000 chemicals currently being studied by the National Center for Advancing Translational Sciences (NCATS) (National Center for Advancing Translational Sciences, 2022). The scope of the annotations in the database is limited to data that could be collected from studying these chemicals.

Tox21Enricher-Shiny accepts input in the form of a single chemical, multiple chemicals, or multiple groups of chemicals. If the input chemicals are in Tox21, the application will provide the user with those chemicals’ properties as they appear among the annotations from Tox21. If the input chemicals are not in Tox21 (and, therefore, have no directly corresponding annotations from Tox21), Tox21Enricher-Shiny allows a user to profile each chemical and infer its own properties based on the properties of its “chemical neighbors” (i.e., chemicals that are similar in some way to the input chemical) within the Tox21 dataset. Tox21Enricher-Shiny determines if chemicals are similar by either checking if an input chemical is a substructure of another chemical or by calculating the structural similarity between an input chemical and another chemical based on a Tanimoto coefficient by calculating the distance between each chemical’s bit vector Morgan fingerprint. A list of similar chemicals in Tox21, if any were found, is then substituted for each input chemical that is not in Tox21 as new input(s).

After a list or lists of chemicals are input by the user or a list is identified by the application via substructure or chemical similarity functions, Tox21Enricher-Shiny will enrich the given input by attaching biological, toxicological, and chemical properties (annotations) associated with the input chemicals. It detects which annotations are significantly overrepresented among the input chemicals and visualizes these data as a series of heatmaps and network graphs. Tox21Enricher-Shiny currently features 39 categories of annotations from various data sources: DrugMatrix, the Comparative Toxicogenomics Database (CTD), DrugBank, PubChem, Leadscope/Instem, Multicase, the Environmental Protection Agency’s (EPA) CompTox Chemicals Dashboard, ToxPrint, the Toxin and Toxin Target Database (T3DB), and the ToxRefDB (Lim et al., 2010; Altamira LLC and Molecular Networks GmbH, 2013; Wishart et al., 2015; USEPA, 2019; EPA, 2022; Instem, 2022; MDI Biological Laboratory and NC State University, 2022a; MultiCASE, 2022; National Library of Medicine, 2022a; 2022b; 2022c; 2022b; 2022c; 2022d U.S. Department of Health and Human Services, 2022; DrugBank, 2023a; 2023b).

Tox21Enricher-Grails (originally called “Tox21 Enricher”) is the older version of the Tox21Enricher-Shiny application and was originally documented in a publication in *Molecular Informatics* in 2018 (Hur et al., 2018). It was built using the Grails framework for Groovy and uses additional scripts in Perl (Hur et al., 2018). The Grails application accepts a chemical or a list of chemicals as input from a user and determines the significantly overrepresented annotations among the input chemical(s) and the input’s chemical neighbors (Hur et al., 2018). The Grails application displays these annotations and their associations in two types of visualizations: heatmaps and network graphs (Hur et al., 2018). Although powerful, the Grails application had a few limitations: outdated annotations, reliance on an old version of the Grails framework, and limited portability and scalability. As we gained access to new annotations, we realized the need for a new iteration of Tox21Enricher that should be more intuitive to use and set up, be easily portable and scalable to different environments, and take advantage of currently supported technology. As such, the Grails version was formally supplanted in 2022 by the newer Tox21Enricher-Shiny version that began development in 2020. Tox21Enricher-Shiny uses the Shiny package for RStudio and primarily uses R to perform its tasks. Compared to the older application, Tox21Enricher-Shiny hosts new features, including the ability to view a list of annotations for a single input chemical or multiple input chemicals, support for inputting chemicals as InChI strings, new data visualizations, and application programming interface (API) functionality. Tox21Enricher-Shiny also includes a completely updated database and a more streamlined architecture for better portability. The application is now more configurable, and the application managers may easily adjust variables to allow the application to perform effectively across different environments. End users can now enrich data with potentially more annotations, which allows for a more thorough analysis. The new interactive visualizations provide alternative, less-technical views of the data, which are easier for some users to interpret. These updates to Tox21Enricher-Shiny could promote more effective research and an increased user base.

## 2 Methods

### 2.1 Software architecture

Tox21Enricher-Shiny consists of three pieces of software: the Shiny application, the API, and the database. All three components must work together for the application’s system to function correctly.

#### 2.1.1 Shiny application

The Shiny application component of Tox21Enricher requires the Shiny package (version 1.6.0) for the R programming language (version 3.6.3) (The R Foundation, 2020; Posit, 2021). It also implements functionality from the JavaScript Molecule Editor (JSME) to allow a user to draw a chemical’s structure (Bienfait and Ertl, 2013). The Shiny application was designed to be able to run on either the same machine as the API and database components or on a separate machine as a remote client. The Shiny application needs to be able to connect to the API component: the connection string to the API from the Shiny application may be defined in the configuration file (config.yml) for the Shiny application.

The Shiny application provides a graphical user interface (GUI) to a user and allows them to submit an enrichment analysis request to the API, interact with data visualizations, and download files generated during the enrichment analysis process.

#### 2.1.2 API

Tox21Enricher’s API serves as a programmatic interface between the Shiny application or a user and the database component. It requires the Plumber (version 1.1.0) package for R (Schloerke and Allen, 2021), as well as the cheminformatics tool RDKit in the form of the RDKit Postgres cartridge (version 3.8) (Landrum, 2021; The RDKit Documentation, 2022). The API may also either run on the same machine as the database or a different device, and the connection string to the database from the API may be defined in the configuration file (config.yml) for the API. The enrichment analysis process no longer uses the Perl scripts that were necessary for Tox21Enricher-Grails.

The API processes all requests to perform enrichment analysis or view annotations. It also provides information to the Shiny application about the Tox21Enricher system, such as the maximum time a request will be saved until it is automatically deleted, the total number of completed requests, and a list of all the annotation categories in the database. A user may interact with the API indirectly through the Shiny application or querying specific API endpoints. A user can write code to directly query the API to automate the enrichment analysis process.

#### 2.1.3 Database

The database component of Tox21Enricher-Shiny contains thirty-nine annotation categories in the database. A comprehensive overview of the current annotation categories is given in the Annotation Category Table (see Supplementary Materials). Descriptions, sources, selection criteria, and annotation counts for the annotation categories can be viewed on the front page of the Shiny web application.^1^

**FIGURE 1:**
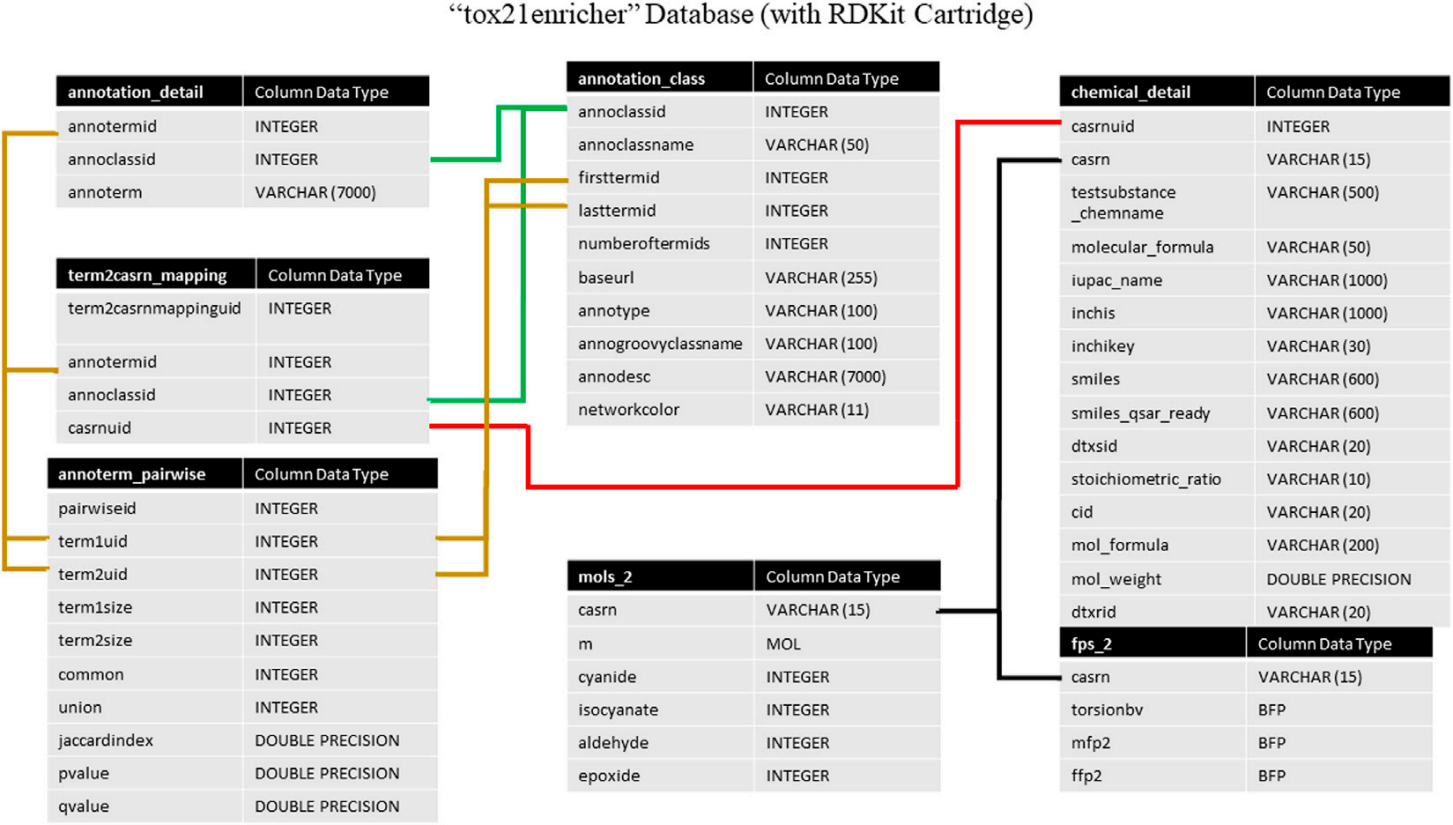
A high-level architectural diagram of the tables in the *tox21enricher* database. This diagram shows each table in each database and how to link across them.

Tox21Enricher’s database system consists of two databases managed by Postgres: *tox21enricher* and *tox21queue* (The PostgreSQL Global Development Group, 2020). The *tox21enricher* database uses the RDKit Postgres cartridge to determine chemical similarity, generate chemical structure images, and convert input InChI strings to SMILES strings (Landrum, 2021; The RDKit Documentation, 2022). The *tox21enricher* database contains all the chemical and annotation information across ten tables. This information includes details for each annotation category; details for each annotation; details for all the chemicals in Tox21; pairwise comparisons for each annotation in the database; and a list of which annotations are associated with each chemical in Tox21. These tables also include information used to calculate chemical similarity. The architecture of this database can be seen in Figure 1. The *annotation_class* table stores attributes for each of the annotation categories, while the *annotation_detail* table stores the names of the individual annotations in each category. The *term2casrn_mapping* table maps each annotation to a related Tox21 chemical’s CASRN. The *annoterm_pairwise* table stores a list of pairwise comparisons between every annotation. The *annotation_matrix* table stores a list of all the annotation IDs for each CASRN in Tox21, while the *annotation_matrix_terms* table maps each annotation ID to the annotation’s name. The *chemical_detail* table is a list of attributes relating to all the chemicals in Tox21 supported by the application. The *mols_2* table maps each chemical’s CASRN to a corresponding “mol” object required during similarity calculations. Finally, the *fps_2* table contains a list of bit vector Morgan fingerprints for each chemical to be used in similarity calculations.

**FIGURE 2:**
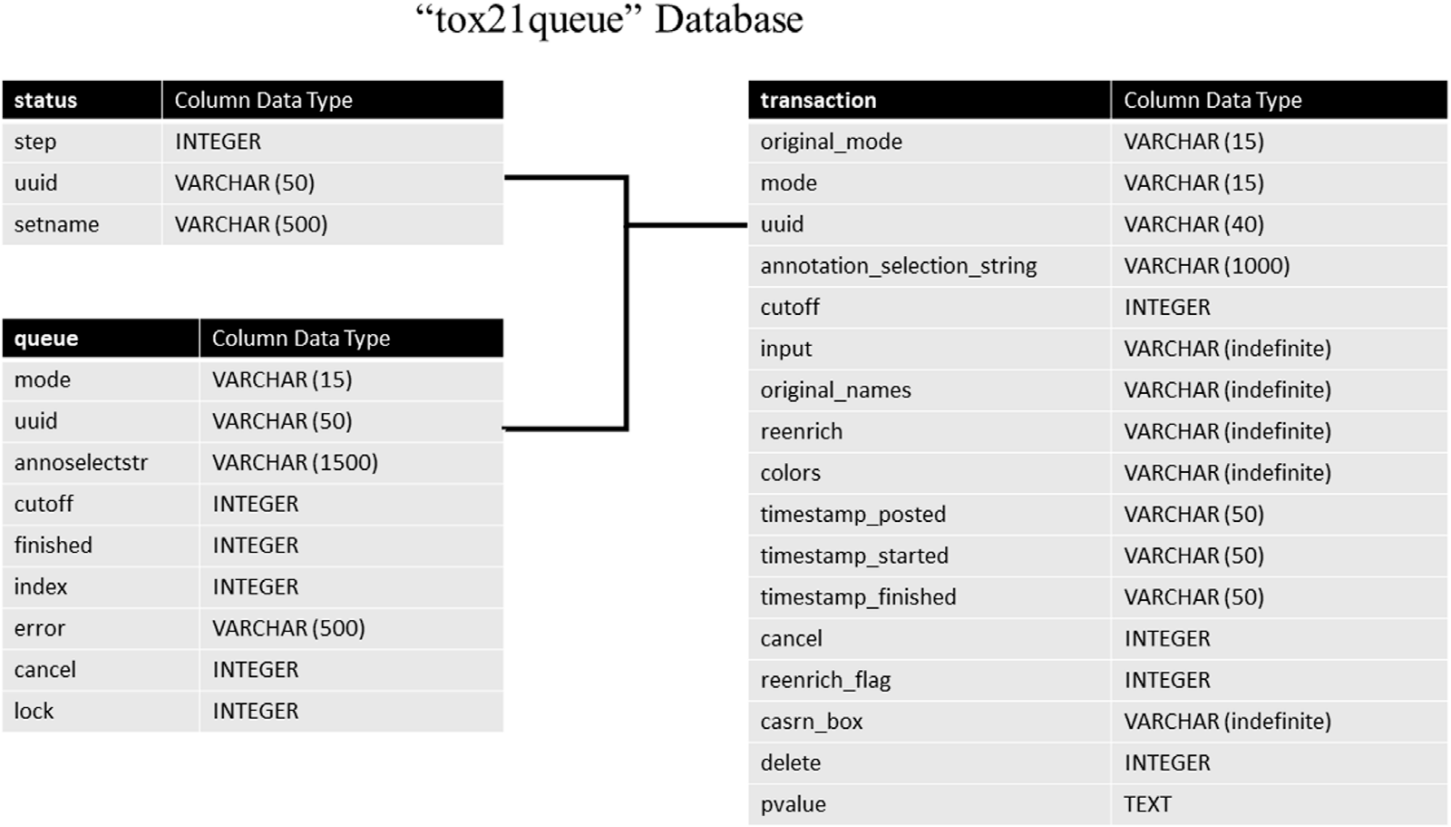
A high-level architectural diagram of the tables in the *tox21queue* database. This diagram shows each table in each database and how to link across them.

The *tox21queue* database contains information related to each request submitted to the API in four tables. This information consists of a unique identifier assigned to the request (Urbanek and Ts’o, 2020); the mode of the request; the various parameters of the request; timestamps for when the request was posted, started, and finished (if applicable); if the request was canceled, and if the request is marked for deletion. The architecture of this database can be seen in Figure 2. The *transaction* table stores in-depth details about each request submitted to Tox21Enricher-Shiny, such as the start and end time, the arguments supplied, and if the request was canceled. The *status* table tracks the stage of each input set for each request in the enrichment analysis process. Finally, the *queue* table stores each request’s overall status and if it has been completed.

**FIGURE 3:**
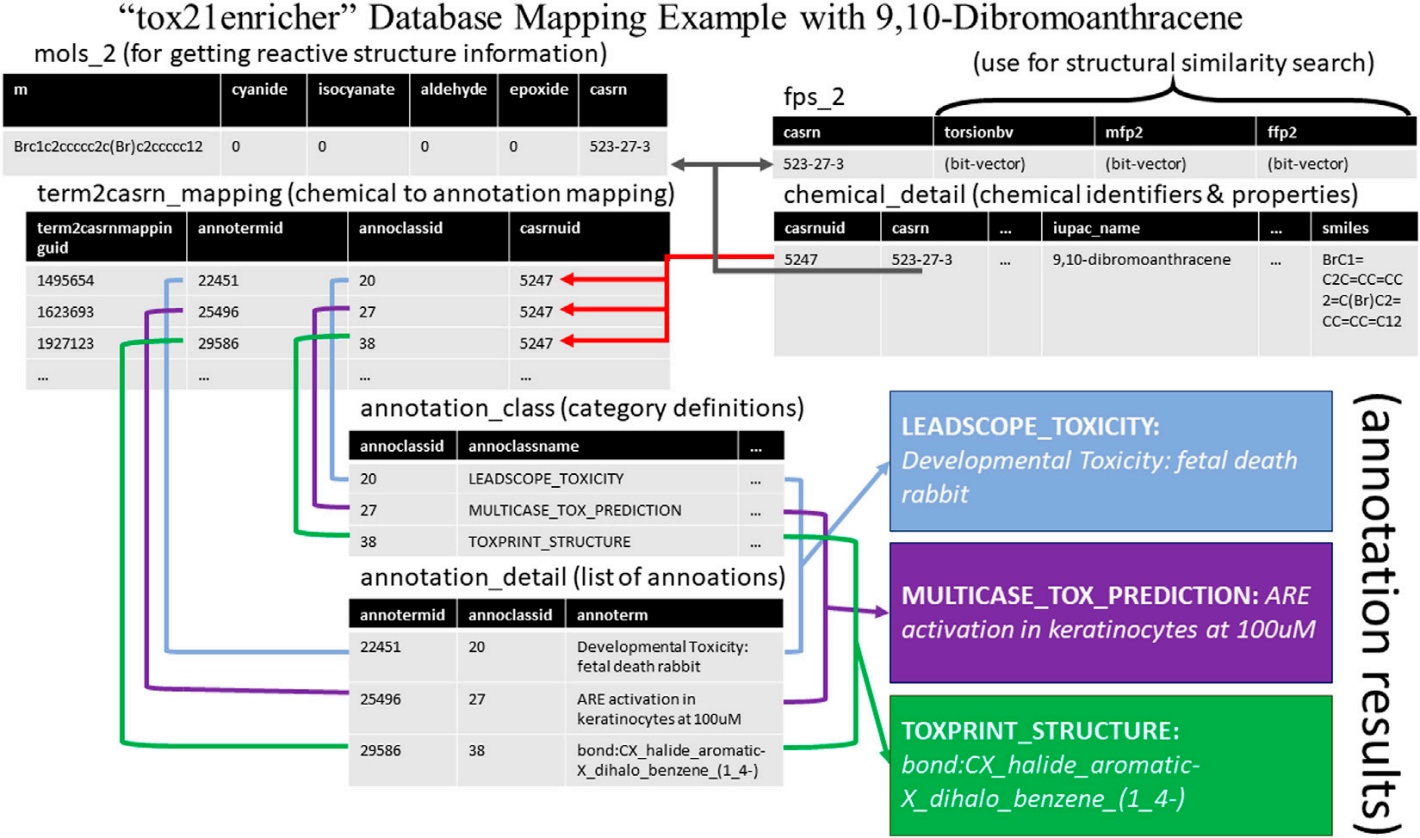
An example of chemical-to-annotation and chemical-to-property mapping using the *tox21enricher* database with 9,10-dibromoanthracene as the input chemical. A user may link together tables in the relational database using certain identifiers in the database. The results shown here are a subset of the full results that may be retrieved using 9,10-dibromoanthracene.

An example of linking across the database tables to match a given chemical to its annotations is demonstrated in Figure 3. This example retrieves a few annotations and their categories as well as some properties for the chemical 9,10-dibromoanthracene.

### 2.2 System description

#### 2.2.1 Software overview

Tox21Enricher-Shiny was designed using the Shiny package for R for consistency with other NIEHS-developed Tox21 applications and because R is well-equipped for working with and creating visualizations for the large amount of data in the Tox21 dataset (U.S. National Toxicology Program, 2023). While Tox21Enricher-Shiny implements HTML and JavaScript code to perform specialized client-side functions, the Shiny application and API components are mostly written in R and integrates powerful R-based visualization tools such as the Plotly, ggplot2, and visNetwork packages that allow for configurable and interactive data visualizations (Wickham, 2016; Sievert, 2020; DataStorm, 2021; Plotly, 2022). Tox21Enricher-Shiny also leverages R functionality like dataframes and the *dplyr* package to easily manipulate large data sets (Wickham et al., 2021). Tox21Enricher-Shiny’s backend consists of two databases: *tox21enricher*, which contains all the chemical and annotation data, and *tox21queue*, which contains information related to requests received by the API component. Both databases are managed by Postgres (The PostgreSQL Global Development Group, 2020), and the *tox21enricher* database is equipped with the RDKit Postgres cartridge (Landrum, 2021; The RDKit Database Cartridge, 2022). The API component is entirely new for the Tox21Enricher-Shiny system and allows for asynchronous and automated request processing. The API was created using the Plumber package for R and provides a Swagger user interface that details the API’s functions and usage (Schloerke and Allen, 2021). A full list of software dependencies used in the development of Tox21Enricher-Shiny can be found in the Software Dependency Table in the Supplementary Materials and the readme file in the project’s GitHub Repository.^2^

#### 2.2.2 Input handling

**FIGURE 4:**
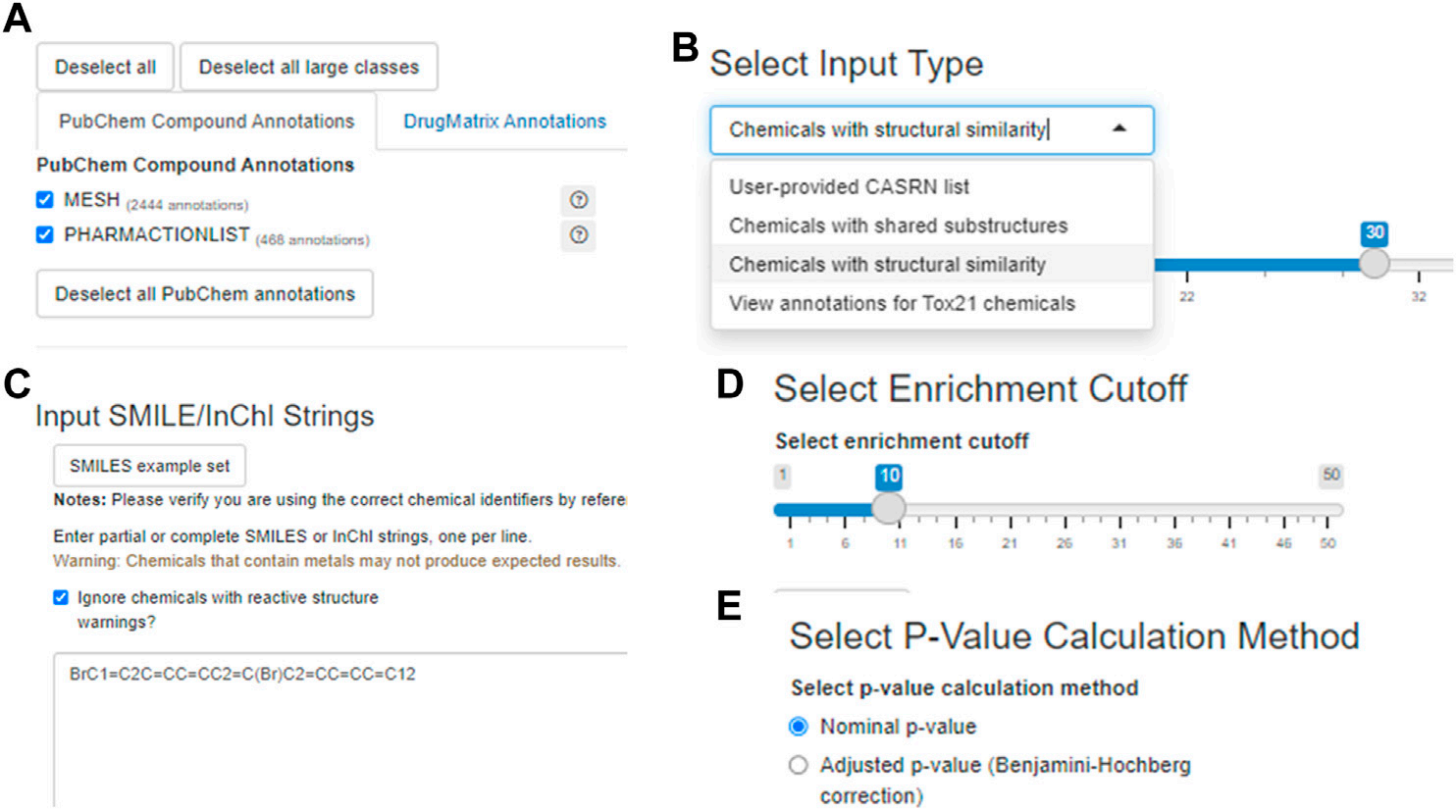
A visualization of some input options for the Shiny application component with 9,10-dibromoanthracene as the example input: the annotation selection menu **(A)** the chemical input mode/type selector **(B)** the text input box in which the user may enter chemical identifier(s) **(C)** the enrichment cutoff selector **(D)** and the *p*-value calculation method selector **(E)**.

The user may specify which annotation categories should be considered by Tox21Enricher-Shiny before submitting a request (Figure 4A). If a category is unchecked in this menu, no annotations from it will appear in the results. The application includes three enrichment input modes: *User-provided CASRN list*, *Chemicals with shared substructures*, and *Chemicals with structural similarity* (Figure 4B). All three modes work similarly: each converts the user-provided list of chemicals into a list of valid CASRN identifiers in the Tox21 database and then calculates significantly overrepresented annotations for this list from the selected annotation categories. However, the method by which the user’s input is translated to compatible CASRNs differs between the modes. For the *User-provided CASRN list* mode, no conversion is performed apart from filtering out CASRNs not in Tox21. For the *Chemicals with shared substructures* mode, Tox21Enricher-Shiny accepts any combination of SMILES and InChI strings as input and maps them to Tox21 chemicals that contain each input chemical as a substructure. For the *Chemicals with structural similarity* mode, Tox21Enricher-Shiny also accepts any combination of SMILES and InChI strings as input and calculates Tanimoto coefficients between the fingerprints of Tox21 chemicals to determine which chemicals are structurally similar to each input chemical. The user may specify the similarity threshold prior to enrichment. Bit vector Morgan (circular) fingerprints using connectivity invariants and a radius of 2 are used in the similarity calculation (Landrum, 2021; The RDKit Database Cartridge, 2022). For the modes that require SMILES or InChI strings as input, a single SMILES or InChI string may be mapped to multiple CASRNs that represent similar chemicals. Tox21Enricher-Shiny also integrates the JSME directly into the Shiny application’s web interface. The JSME may be used to draw a chemical structure within the application and generate a corresponding SMILES string that may be copied into the Shiny application’s input form (Bienfait and Ertl, 2013).

#### 2.2.3 Modes of operation

Tox21Enricher-Shiny’s main mode of operation is to enrich a given list of chemicals with read-across data corresponding to related chemicals in the Tox21 dataset. The platform employs a modified Fisher’s exact test to identify overrepresented (*p* < 0.05) annotations within the chemical list. The *p*-values calculated using this method are included in the enrichment results in nominal form as well as with Bonferroni, Benjamini-Yekutieli, and Benjamini-Hochberg correction (Benjamini and Hochberg, 1995; Benjamini and Yekutieli, 2001; Abdi, 2007). This approach is like those implemented in various gene set functional enrichment analysis tools, such as the Database for Annotation, Visualization, and Integrated Discovery (DAVID) (Hosack et al., 2003; Huang et al., 2007; Sherman et al., 2022). Annotation overrepresentation is sometimes given in the application using the equation: $-1\;*\;\log10\left(p\right)$ . If an annotation is especially overrepresented (i.e., p is effectively 0), this score is set to a maximum value of 500, which we have chosen arbitrarily.

Significantly enriched annotations may undergo chemical content-based clustering to reduce redundancy, which adopts the gene annotation clustering method implemented in DAVID (Sherman et al., 2022). Briefly, kappa scores are calculated to measure the degree of annotation overlap between pairs of annotations in terms of chemicals. Seeds, each containing annotations with high kappa scores that indicate a high level of overlap, are subjected to heuristic fuzzy multiple-linkage partitioning. This process iteratively merges seeds (annotations) based on their chemical content overlap, measured by kappa score.

Tox21Enricher-Shiny allows the user to filter certain chemicals that contain specific reactive substructures out of a request (Sushko et al., 2012). When a user performs enrichment analysis with these chemicals, the request may not produce expected results since these structures can covalently interact with proteins in cell-based assays. By default, Tox21Enricher-Shiny ignores such chemicals if included in the input (Figure 4C). If the user deselects the “Ignore chemicals with reactive (sub)structure warnings?” checkbox on the input form, the application will instead check if the most reactive functional groups (nitrile, isocyanate, aldehyde, and/or epoxide groups) are present exclusively in either the input or at least one of the similar chemicals. If Tox21Enricher-Shiny detects a discrepancy, i.e., a reactive group is present only in the input chemical or only in a similar chemical returned by the chemical search, the similar problematic chemicals will be flagged with a warning on the results page for the request. The user may deselect these chemicals on the results page and resubmit the request without them. These four groups are derived from a larger set (Sushko et al., 2012), and the subset that is employed in this context is based on expert observations from *in vitro* assays (e.g., Tox21). It is important to note that this is not an exhaustive list of reactive functional groups, but it does account for certain reactivity-based discrepancies in concordant behavior (Sushko et al., 2012). Before submitting the request, the user may also adjust the enrichment cutoff, which is the maximum number of annotations to include from a single category in the results (Figure 4D). The user may also toggle if they want to filter the resulting annotations by their nominal *p*-values or by their Benjamini-Hochberg-corrected *p*-values (Figure 4E).

A user may also view all annotations in the database associated with given input chemicals by using the *View annotations* mode. This mode accepts only CASRNs within Tox21 as input. CASRNs not in Tox21 are dropped prior to sending the request, much like the *User-provided CASRN list* mode. The input may be in the form of a single CASRN, multiple CASRNs, or multiple groups of CASRNs with each set given a unique set name. The user may specify which annotation categories should be represented in the results. This mode of request will generate a file for each input chemical that contains each annotation (if any) on a separate line. It will also generate a matrix file that shows a present (1) or not present (0) relationship between each input chemical and each annotation. This matrix is also represented as an interactive and configurable heatmap graph.

**FIGURE 5:**
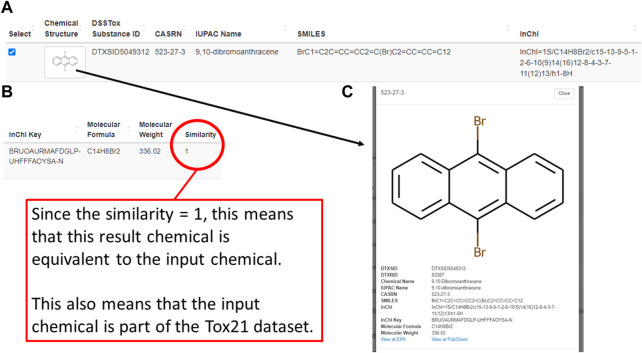
A depiction of one similar chemical (9,10-dibromoanthracene) matched using the structural similarity search mode of the Shiny application’s enrichment analysis process. This figure shows the chemical’s entry in a table on the results page **(A,B)** as well as its detailed view **(C)**, which can be viewed by clicking on the image of the chemical’s chemical structure.

Following a successful enrichment request, Tox21Enricher-Shiny includes a table with additional info for each Tox21 chemical mapped to the input on the results page. Each row of the table includes an image of the chemical’s structure; chemical identifiers in the form of a DSSTox substance ID, CASRN, IUPAC name, SMILES string, InChI string, and InChI key; the chemical’s molecular formula and weight; the chemical’s Tanimoto coefficient (if using the *Chemicals with structural similarity* mode); and a reactive chemical substructure warning, if applicable (Figure 5A; Figure 5B). Clicking the structure image in each row will open a modal menu with a larger representation of the chemical’s structure, additional chemical details, and links to view the chemical’s entry on the PubChem website and the United States EPA CompTox Chemicals Dashboard if one exists (Figure 5C). A user may select some or all similar chemicals from each set in this table to perform re-enrichment with the selected chemicals only. Unselected chemicals will not be processed during re-enrichment and will no longer show up on subsequent results pages.

#### 2.2.4 Data visualization

**FIGURE 6:**
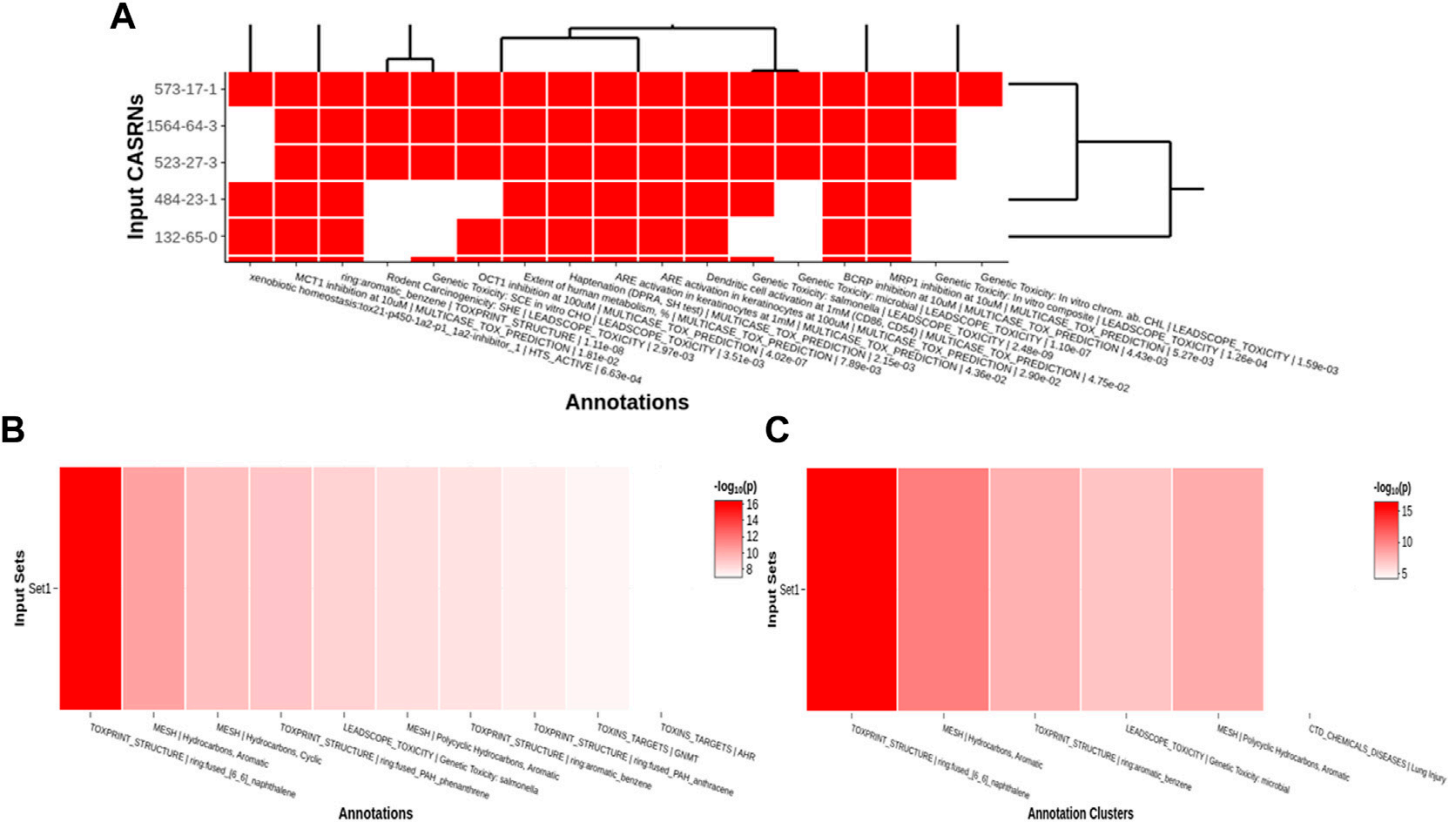
An example of per set **(A)**, chart **(B)**, and cluster **(C)** heatmaps for a given chemical input. For the per set heatmap, values are binary: a white square means that a chemical is not associated with a given annotation, while a red square means that a chemical is associated with an annotation. The chart and cluster heatmaps depict the $-\log_{10}\left(p\right)$ values of the most significant annotations across all input sets.

Tox21Enricher-Shiny uses the Plotly (version 4.9.3) library for R to dynamically generate interactive heatmaps (Sievert, 2020; Plotly, 2022) after enriching the input data. If sufficient result data are available, the application generates a heatmap per input set (“set heatmap”) to show which CASRNs in the set are associated with each annotation (Figure 6A). The annotations in this plot are clustered by row and column (shown by a dendrogram). It also generates one heatmap that depicts which input sets are associated with each of the most significant annotations across all sets (“chart heatmap”) (Figure 6B) and another heatmap that depicts which input sets are associated with clusters (the same clustering process described in Section 2.2.3) of similar annotations (“cluster heatmap”) (Figure 6C).

A user may pan across and zoom in and out of each heatmap. Hovering over each cell in a set heatmap will display a tooltip with the CASRN and annotation corresponding to that cell and if there is (shown as “1”) or is not (shown as “0”) a relationship between the chemical and the annotation. Hovering over each cell in the chart or cluster heatmap will display a tooltip with the set name and annotation corresponding to that cell and its significance, given by either a raw *p*-value or a Benjamini-Hochberg-corrected *p*-value (Benjamini and Hochberg, 1995).

**FIGURE 7:**
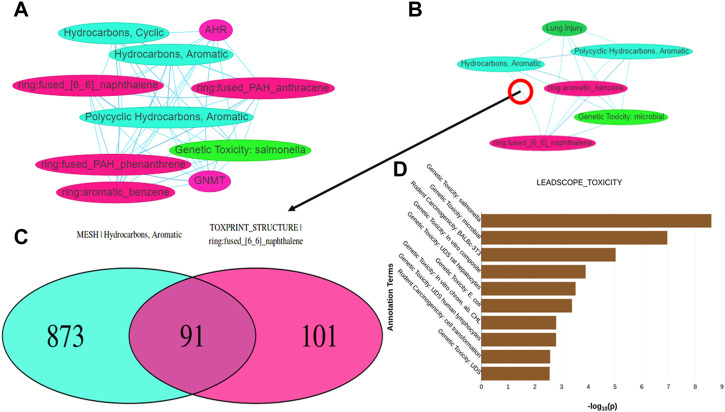
Depictions of other data visualization tools in the Shiny interface: **(A,B)** show network graphs for the chart and cluster files, respectively. When clicking on an edge between two nodes in either type of graph, a Venn diagram **(C)** is generated showing the number of chemicals associated with the annotations represented by the nodes. **(D)** displays a bar graph per annotation category found in the results that shows which annotations in each category are significantly overrepresented within any of the input sets.

Tox21Enricher-Shiny also can generate interactive network graphs using the visNetwork package that represents each significant annotation as a node (DataStorm, 2021). Each edge in the network represents the overlap of the chemicals associated with each annotation corresponding to the edge’s nodes. The significant overlap of chemicals associated with each annotation is calculated based on a modified Fisher’s exact test using the R “stats” package (R Core Team, 2020) and may be adjusted for multiple tests using Benjamini-Hochberg correction if specified by the user (Benjamini and Hochberg, 1995). The application creates one network (“chart network”) (Figure 7A) that includes the most significantly overrepresented annotations for each input set. Tox21Enricher-Shiny also generates a network (“cluster network”) (Figure 7B) that compiles the most significantly overrepresented clusters of annotations from each input set (as described in Section 2.2.3). The user may interact with the network graphs by setting the Benjamini-Hochberg-corrected q-value to determine the significance necessary for an annotation to be included in the graph (Benjamini and Hochberg, 1995); selecting which input sets should be represented in the graph; and viewing additional information about each annotation when its corresponding node is clicked. A user may also show or hide annotations belonging to certain annotation categories, enable a graph’s physics simulation, toggle a graph’s edges between straight and curved, and export a graph as a static Portable Network Graphics (PNG) image. Selecting any edge of a graph generates a color-coded Venn diagram that shows how many chemicals are associated with each annotation represented in the edge’s two nodes (Figure 7C). The user may click buttons to the side of this Venn diagram to view a list of chemicals associated with each annotation and a list of chemicals associated with both annotations. The Venn diagram is a static visualization and may be downloaded in either PNG or PDF formats.

Tox21Enricher-Shiny includes another data visualization in the form of a series of bar charts at the bottom of the results page after data enrichment is completed (Figure 7D). A bar chart is created for each annotation category represented among the most significant annotations for each input set. The maximum number of annotations in each bar chart is N×I , where *N* is a user-defined cutoff value that determines the number of significantly overrepresented annotations to include per category, and *I* is the number of input sets. Each bar chart depicts the *p*-value for each of the overrepresented annotations in a specific category. The bars on each chart are color-coded to match each input set. Under each bar chart is a table that represents the data in the bar chart as text.

#### 2.2.5 Request handling

Tox21Enricher-Shiny was designed to process requests asynchronously from when they were received to ensure the availability of the system’s resources. When a user submits a request from Tox21Enricher-Shiny’s Shiny application component or by directly querying the API component, the API saves information relating to that request into the *tox21queue* database for further reference. If multiprocessing is enabled, the API then spawns a new thread from the main process in which the request is carried out in the background using the future package for R (Bengtsson, 2021). After this quick initial processing stage, the API sends a response back to the client, thus preventing the need to maintain a live connection to the API for the duration of the request’s lifetime. As the API is performing work on the request, it will periodically update the *tox21queue* database with the request’s status. As the Shiny application is waiting for the request’s work to complete, it will continually query the API to retrieve the status of the work process and display it to the user via the web interface. When the request is finished, the API updates the database entry for the request to signify that the request has been completed: the Shiny application will then start loading the request’s results to display to the user.

If the API unexpectedly ceases functioning and must be restarted for whatever reason, Tox21Enricher-Shiny will now also reprocess any requests that were currently being processed (without error) when the API failed. These requests will begin processing from the beginning and not from the stage they were at when the API failed: this is to ensure that each request can be processed fully without the chance of data being lost. Requests may also now be “canceled” by the user from within the Shiny application. If a user opts to cancel their request, the request’s entry in the database will be updated to signify that the request was canceled. This process does not remove the request’s records from the database, nor does it remove any files created by the request on the computer running the API’s filesystem; rather, it just sets a flag so that the components of Tox21Enricher-Shiny will ignore this request when showing previous requests to the user. A database administrator or manager must archive or remove database records and generated files from the filesystem by using a provided R script or by doing so manually.

Detailed instructions with interface images for performing enrichment analysis are included in the Tox21Enricher-Shiny user manual, which is included as Supplementary Materials.

#### 2.2.6 User experience

Tox21Enricher-Shiny implements some convenience features to improve user experience. In addition to the ability to process requests asynchronously and the added documentation, Tox21Enricher-Shiny also features the ability to view a previous request’s results, which was a possible but unsupported feature in Tox21Enricher-Grails. Each request is now assigned a 32-character unique identifier string (not including hyphens) created with the “uuid” R package (Urbanek and Ts’o, 2020). When a user submits a request, this identifier is displayed on the waiting page and can be copied to the user’s clipboard by clicking the “Copy UUID to clipboard” button on that page (rclipboard, 2023). Additionally, if the user’s browser allows cookies, the Shiny application will save a temporary cookie with the request’s identifier string to the user’s browser storage. The user can click the “View previous results” button from the main page of the Shiny application to view an interface that allows them to input the identifier of a previous request or select a previously run request from a list to view the results of the provided request. This list will only appear if the Shiny application can detect saved cookies detailing previous requests on the browser. The Shiny application component also features tooltips describing the functionality of certain inputs and interface elements and details of the annotation categories in the database.

#### 2.2.7 Security

Tox21Enricher-Shiny’s previously discussed unique identifier strings supplant Tox21Enricher-Grails’s identification system, which was to assign each new request with a single, incremental integer. The original system would make it trivial to guess the identifiers of and snoop on other users’ requests. It is more difficult to guess a 32-character alphanumeric string used previously by the system. Tox21Enricher-Shiny’s API component also employs input validation measures to prevent SQL injection attacks and viewing and interacting with the API host machine’s filesystem. The Tox21Enricher-Shiny system has the *tox21enricher* and *tox21queue* databases managed by two different users with limited permissions on each other’s databases: this is to prevent access to the other database if one account is compromised.

#### 2.2.8 Differences from Tox21Enricher-Grails

Tox21Enricher-Shiny changes and expands upon Tox21Enricher-Grails in multiple areas to improve usability, scalability, and availability. Tox21Enricher-Grails’s backend code relied on two databases: one database used MySQL and contained all the annotation-related information, while the other database used Postgres and contained information relating to the chemicals in the Tox21 dataset as well as the RDKit Postgres cartridge functionality. Connections to these databases from the Grails application had to be configured differently and separately. To make the database component easier to maintain and update, all the data from the MySQL-managed database was converted and imported into the Postgres database. Tox21Enricher-Shiny’s *tox21enricher* database in the Postgres database is the result of merging the two databases into a single, Postgres-managed database with the RDKit cartridge installed. Tox21Enricher-Shiny adds support for asynchronous and automated behavior to prevent a single long request from monopolizing the system’s resources. Tox21Enricher-Grails originally did not support InChI strings as input, but they are now supported in the Shiny version.

Tox21Enricher-Shiny adds additional functionalities to those present in the Grails application: new data visualizations, the ability to view all annotations for a given chemical(s), and the ability to view previous requests. Tox21Enricher-Shiny also implements additional filtering on clusters derived from annotations during enrichment so that only clusters with an enrichment score greater than 1 are included in the results.

The Tox21Enricher-Grails annotation database contained 36 separate annotation categories. Most of these original categories are preserved in Tox21Enricher-Shiny, but five categories from the Grails application were not transferred, six categories are new, four are adapted into new categories, and two are adapted into separate categories. A category called ZERO_CLASS existed as a fallback label that would be assigned to an annotation if it had no assigned category in Tox21Enricher-Grails. Because the database was reconstructed to ensure no annotation was missing a category, the ZERO_CLASS category became obsolete and was thus removed in Tox21Enricher-Shiny. Four other categories were removed: CTD_SF, MESH_LEVEL1, MESH_LEVEL2, and MESH_LEVEL3. The CTD_CHEMICALS_GOENRICH_CELLCOMP, CTD_CHEMICALS_GOENRICH_MOLFUNCT, DRUGBANK_ATC, DRUGBANK_CARRIERS, DRUGBANK_ENZYMES, and DRUGBANK_TRANSPORTERS are completely new categories. CTD_CHEM2DISEASE, CTD_CHEM2GENE_25, CTD_PATHWAY, and TOXCAST are now relabeled to CTD_CHEMICAL_DISEASES, CTD_CHEMICALS_GENES, CTD_CHEMICALS_PATHWAYS, and TOXCAST_ACTIVE, respectively. CTD_GO_BP is now CTD_GOFAT_BIOPROCESS with an additional subset, CTD_GOSLIM_BIOPROCESS. HTS_ACTIVE has an additional subset called HTS_STRONGACTIVE. Due to these annotation changes, Tox21Enricher-Shiny may return different annotations for a given input than the Grails application would for the same input.

### 2.3 Documentation and examples

Detailed instructions for the installation and deployment of each component of the system are available in the Tox21Enricher-Shiny GitHub repository.^3^ This document (Setup Guide) is also supplied as Supplementary Material.

#### 2.3.1 Documentation

Two major instructional documents, the user manual and the setup guide, are included with Tox21Enricher-Shiny. The user manual details the steps for a user to properly interact with and perform the functions of an already-running instance of the Shiny web application. The setup guide provides instructions for obtaining the necessary code and dependencies and configuring and deploying the project. The user manual is intended for the average system end-user, while the setup guide is designed for a server administrator who is trying to deploy an instance of Tox21Enricher-Shiny onto a host. In addition to these two documents, Tox21Enricher-Shiny also includes documentation in other areas. The configuration files for the Shiny application and API are annotated with instructions on how to set each parameter. The code itself for the Shiny application and API is thoroughly commented on.

#### 2.3.2 Examples

Tox21Enricher-Shiny includes a few examples to demonstrate the use cases of the application. The Shiny application contains three built-in example sets that can be run from the main page of the web application to explore the intended output of the data enrichment process. An example shell script is also included to test the direct user-to-API interactions. This script demonstrates how to construct a query to submit a request directly to the API. The script is annotated with instructions detailing how to set each argument of the request string. The code in this script should be used as an example when developing code to do automated querying.

## 3 Results

### 3.1 Example use cases

This section will detail a few practical example use cases for Tox21Enricher-Shiny and their results. Each example was run using the input specified in its respective section: if an input option is not specified as being changed, then it was left as the default value for that example. Tox21Enricher-Shiny’s default values are as follows: all annotation categories enabled except for CTD_GOFAT_BIOPROCESS; an enrichment cutoff of 10; annotation filtering using the nominal *p*-value; a Tanimoto similarity of 50% (if using the “Chemicals with structural similarity” input type); and the setting to ignore chemicals with reactive structure warnings enabled.

The data in the tables supporting each example below are derived from the “Chart Simple” files produced by the application given each example input. Each “Chart Simple” file includes some of the annotations found to be the most significantly overrepresented among each example’s respective input chemicals. Only the top *N* annotations per category are included in this file, where *N* is the user-defined enrichment cutoff. Each table from 1 to 8 below depicts a subset of annotations relevant to a certain effect or endpoint. Each of these tables includes the annotation category name, the annotation name, the number of chemicals from the input set that the annotation is found in (as both a number and percentage), and the *p*-value calculated from the Fisher’s exact test (to determine overrepresentation) for each annotation pulled directly from the corresponding “Chart Simple” file. The “results” tables in the supplementary spreadsheet (“Tox21Enricher-Example Results and Chemical Lists”) are pulled directly from the “Chart” files generated for each example, which contain the same information as the corresponding “Chart Simple” file as well as a list of relevant CASRNs, an enrichment score, adjusted *p*-values, and additional statistics relating to the annotation’s presence across the entire Tox21 dataset for each annotation. The “chemical list” tables in the supplementary spreadsheet (“Tox21Enricher-Example Results and Chemical Lists”) contain the inputs used for each example. The chemical list tables were constructed using Tox21Enricher-Shiny’s chemical search feature. In example 1, the application returned chemicals in Tox21 that contain the input chemical as a substructure. In example 2, the application returned chemicals in Tox21 that are structurally similar to the input chemical. In example 3, the chemical list table was created using external tools, and these chemicals were supplied directly as CASRNs as the input set to Tox21Enricher-Shiny.

#### 3.1.1 Example 1: bisphenol class analysis using substructure search

Toxicologists often study classes of chemicals that are defined by a specific substructure. One class of compounds that has seen a great deal of research in recent years is bisphenols (Pelch et al., 2019). Bisphenols are used in the manufacturing of plastic, which has spurred public health concerns due to widespread human exposure. To get an overall view of the various toxicological and chemical characteristics of the bisphenols, we performed a search for chemicals with shared substructures using the base bisphenol substructure (SMILES: OC1 = CC = C(CC2 = CC = C(O)C=C2)C=C1). To obtain a broader view of the class, the enrichment cutoff was set to 25 instead of the default 10; therefore, up to 25 relevant annotations per category could be included in the results. This search returned seventy-six chemicals, which were then used for chemical annotation enrichment (see the bisphenol chemical list in the supplementary spreadsheet, “Tox21Enricher-Example Results and Chemical Lists”). Because we are doing a substructure search, the chemical structure information is not particularly valuable. Hence, we focus on the biological and toxicological properties of the chemical class. A review of the enriched Leadscope-predicted toxicities shows the top-ranked toxicities to be related to reproductive toxicity in multiple species, consistent with the class’s predicted effects on hormonal signaling (Table 1).

**TABLE 1:** A summary of the most significantly overrepresented annotations relating to reproductive toxicity as returned by Tox21Enricher-Shiny using bisphenols as the chemical input. For each row: the “Category” column refers to the source of the annotation; the “Term” column is the annotation name; the “Count” column refers to how many of the 76 input chemicals are associated with the given annotation; the “%” column is the “Count” column’s value converted to a percentage; and “*p*-value” column is the statistical significance of the annotation’s representation calculated using a modified Fisher’s exact test.

Table 1				
Category	Term	Count	%	*p*-value
MULTICASE_TOX_PREDICTION	MRP2 substrates	61	80.26	0
MULTICASE_TOX_PREDICTION	OCT1 substrates	50	65.79	0
LEADSCOPE_TOXICITY	Reproductive Toxicity: repro rat female	26	34.21	0
LEADSCOPE_TOXICITY	Reproductive Toxicity: repro rodent female	29	38.16	0
MULTICASE_TOX_PREDICTION	MRP1 substrates	54	71.05	0
MULTICASE_TOX_PREDICTION	OATP2B1 inhibition at 100uM	48	63.16	0
MULTICASE_TOX_PREDICTION	Tox21 ARE activation assay, strong and weak actives by NIEHS calls, research only	46	60.53	0
MULTICASE_TOX_PREDICTION	Human plasma protein binding, %bound	54	71.05	0
MULTICASE_TOX_PREDICTION	ARE activation in keratinocytes at 1 mM	52	68.42	0
MULTICASE_TOX_PREDICTION	MRP4 substrates	3	3.95	0
LEADSCOPE_TOXICITY	Genetic Toxicity: SCE *in vitro* CHO	13	17.11	0
LEADSCOPE_TOXICITY	Genetic Toxicity: chrom. ab. other rodent	20	26.32	0
MULTICASE_TOX_PREDICTION	Tox21 ARE activation assay, only strong actives by NIEHS calls, research only	38	50.00	0
LEADSCOPE_TOXICITY	Genetic Toxicity: micronucleus mouse	24	31.58	0
LEADSCOPE_TOXICITY	Reproductive Toxicity: repro mouse female	17	22.37	0
MULTICASE_TOX_PREDICTION	RCA survival pre-implantation, rodent	18	23.68	0
LEADSCOPE_TOXICITY	Genetic Toxicity: micronucleus rodent	22	28.95	0
LEADSCOPE_TOXICITY	Rodent Carcinogenicity: cell transformation	9	11.84	0
LEADSCOPE_TOXICITY	Developmental Toxicity: pre impl. loss rat	22	28.95	0
MULTICASE_TOX_PREDICTION	Draize eye irritation test, rabbit	56	73.68	0
MULTICASE_TOX_PREDICTION	NTCP substrates	25	32.89	0
MULTICASE_TOX_PREDICTION	BSEP inhibition at 100uM	47	61.84	0
MULTICASE_TOX_PREDICTION	RCA fetal weight decrease, rodent	22	28.95	0
MULTICASE_TOX_PREDICTION	Local lymph node assay, mouse, weak sensitizers (EC3 < 100%)	54	71.05	0
LEADSCOPE_TOXICITY	Developmental Toxicity: retardation rat	23	30.26	0
MULTICASE_TOX_PREDICTION	Human volume of distribution (L/kg)	12	15.79	0

Consistent with the anticipated toxicological mode of action of the bisphenol class, the ToxCast and Tox21 strong actives indicated enrichment for activity in the estrogen and androgen-related pathways, specifically activation of estrogen receptors (ER) and inhibition of androgen receptors (AR) (Table 2). These would be consistent with the enrichment of the reproductive toxicity properties noted above. There is also an indication of some activity on other steroid receptors, including glucocorticoid receptor and progesterone receptor, in addition to potential effects on mitochondrial membrane potential. Finally, some associations suggest that bisphenols may be autofluorescent, which is valuable information if one is developing *in vitro* assays. These findings are consistent with the enrichment of CTD genes, DrugBank targets, and toxin targets related to sex steroid nuclear receptor signaling (estrogen receptor 1 (ESR1), progesterone receptor (PGR), estrogen receptor 2 (ESR2), AR) and metabolism (cytochrome P450 family 19 subfamily A member 1 (CYP19A1)). Other less certain associations are with topoisomerase (TOP2A) and glutathione-S-transferase (GSTP1) (Table 3). Overall, the results provide a broad-level profile with a focus on certain endpoints of concern, such as endocrine disruption and reproductive function, which is consistent with the literature.

**TABLE 2:** A summary of the most significantly overrepresented annotations relating to estrogen/androgen pathways, steroid receptor activity, mitochondrial membrane potential, and autofluorescence as returned by Tox21Enricher-Shiny using bisphenols as the chemical input. For each row: the “Category” column refers to the source of the annotation; the “Term” column is the annotation name; the “Count” column refers to how many of the 76 input chemicals are associated with the given annotation; the “%” column is the “Count” column’s value converted to a percentage; and “*p*-value” column is the statistical significance of the annotation’s representation calculated using a modified Fisher’s exact test.

Table 2				
Category	Term	Count	%	*p*-value
HTS_STRONGACTIVE	sex hormone homeostasis:tox21-er-luc-bg1-4e2-agonist-p4_er-agonist_1	18	23.68	0
HTS_STRONGACTIVE	sex hormone homeostasis:tox21-erb-bla-antagonist-p1_erb-antagonist_1	25	32.89	0
TOXCAST_ACTIVE	NVS_NR_hER	14	18.42	0
HTS_STRONGACTIVE	sex hormone homeostasis:tox21-er-bla-antagonist-p1_er-antagonist_1	19	25.00	0
TOXCAST_ACTIVE	NVS_NR_bER	12	15.79	0
HTS_STRONGACTIVE	sex hormone homeostasis:tox21-ar-bla-antagonist-p1_ar-antagonist_1	23	30.26	0
HTS_STRONGACTIVE	xenobiotic homeostasis:tox21-car-antagonist-p1_car-antagonist_1	15	19.74	0
HTS_STRONGACTIVE	glucocorticoid homeostasis:tox21-gr-hela-bla-antagonist-p1_gr-antagonist_1	20	26.32	0
TOXCAST_ACTIVE	OT_ER_ERaERa_1440	11	14.47	0
HTS_STRONGACTIVE	sex hormone homeostasis:tox21-pr-bla-antagonist-p1_pr-antagonist_1	24	31.58	0
HTS_STRONGACTIVE	autofluorescence:tox21-spec-hek293-p1_autofluor@cell-red-n_1	9	11.84	0
TOXCAST_ACTIVE	NVS_NR_mERa	12	15.79	0
HTS_STRONGACTIVE	autofluorescence:tox21-spec-hek293-p1_autofluor@med-red-n_1	9	11.84	0
HTS_STRONGACTIVE	counter screen (cell viability):tox21-er-luc-bg1-4e2-agonist-p4_viability_1	16	21.05	0
HTS_STRONGACTIVE	mitochondrial membrane permeability:tox21-mitotox-p1_mmp-inhibitor_1	26	34.21	0
TOXCAST_ACTIVE	NVS_NR_rAR	11	14.47	0
TOXCAST_ACTIVE	NVS_NR_hGR	13	17.11	0
HTS_STRONGACTIVE	lipid homeostasis:tox21-pparg-bla-antagonist-p1_pparg-antagonist_1	18	23.68	0
TOXCAST_ACTIVE	OT_ER_ERaERa_0480	11	14.47	0
HTS_STRONGACTIVE	autofluorescence:tox21-spec-hepg2-p1_autofluor@med-red-n_1	9	11.84	0
HTS_STRONGACTIVE	sex hormone homeostasis:tox21-er-luc-bg1-4e2-agonist-p2_er-agonist_1	20	26.32	0
HTS_STRONGACTIVE	autofluorescence:tox21-spec-hek293-p1_autofluor@red_1	9	11.84	0
HTS_STRONGACTIVE	genotoxic stress:tox21-p53-bla-p5_p53-agonist_1	16	21.05	0
HTS_STRONGACTIVE	xenobiotic homeostasis:tox21-p450-2c9-p1_2c9-inhibitor_1	43	56.58	0
TOXCAST_ACTIVE	OT_ERa_EREGFP_0480	11	14.47	0
HTS_STRONGACTIVE	counter screen (cell viability):tox21-casp3-cho-p1_viability@cho-k1_1	27	35.53	0
HTS_STRONGACTIVE	autofluorescence:tox21-spec-hepg2-p1_autofluor@red_1	9	11.84	0
HTS_STRONGACTIVE	counter screen (cell viability):tox21-er-luc-bg1-4e2-antagonist-p2_viability_1	13	17.11	0
HTS_STRONGACTIVE	autofluorescence:autofluor@med-red-n_1	9	11.84	0
HTS_STRONGACTIVE	autofluorescence:autofluor@med-red-n_2	9	11.84	0
TOXCAST_ACTIVE	OT_ER_ERaERb_0480	12	15.79	0
HTS_STRONGACTIVE	autofluorescence:tox21-spec-hepg2-p1_autofluor@cell-green_1	6	7.89	0
HTS_STRONGACTIVE	autofluorescence:tox21-spec-hepg2-p1_autofluor@med-green_1	6	7.89	0
HTS_STRONGACTIVE	sex hormone homeostasis:tox21-ar-mda-kb2-luc-antagonist-p2_ar-antagonist_1	21	27.63	0
TOXCAST_ACTIVE	OT_ERa_EREGFP_0120	11	14.47	0
HTS_STRONGACTIVE	sex hormone homeostasis:tox21-er-bla-agonist-p2_er-agonist_1	13	17.11	0
TOXCAST_ACTIVE	NVS_GPCR_hAdoRA1	8	10.53	0
HTS_STRONGACTIVE	counter screen (cell viability):tox21-ks-are-p1_viability_1	19	25.00	0
HTS_STRONGACTIVE	cytotoxicity:tox21-rt-viability-hek293-p1_viability@glo_1	33	43.42	0
HTS_STRONGACTIVE	autofluorescence:tox21-spec-hek293-p1_autofluor@med-green-n_1	8	10.53	0
HTS_STRONGACTIVE	counter screen (cell viability):tox21-er-luc-bg1-4e2-antagonist-p1_viability_1	15	19.74	0
HTS_STRONGACTIVE	autofluorescence:tox21-spec-hek293-p1_autofluor@cell-green-n_1	8	10.53	0
HTS_STRONGACTIVE	autofluorescence:tox21-spec-hepg2-p1_autofluor@cell-red-n_1	7	9.21	0
TOXCAST_ACTIVE	OT_ER_ERaERb_1440	11	14.47	0
TOXCAST_ACTIVE	OT_ER_ERbERb_0480	11	14.47	0
HTS_STRONGACTIVE	autofluorescence:tox21-spec-hek293-p1_autofluor@green_1	8	10.53	0
HTS_STRONGACTIVE	genotoxic stress:tox21-p53-bla-p1_p53-agonist_1	14	18.42	0
HTS_STRONGACTIVE	autofluorescence:tox21-spec-hek293-p1_autofluor@med-green_1	5	6.58	0
TOXCAST_ACTIVE	Tox21_ERa_LUC_BG1_Agonist	12	15.79	0
HTS_STRONGACTIVE	counter screen (cell viability):tox21-casp3-hepg2-p1_viability@hepg2_1	16	21.05	0
HTS_STRONGACTIVE	autofluorescence:autofluor@med-green-n_1	9	11.84	0
HTS_STRONGACTIVE	autofluorescence:tox21-spec-hek293-p1_autofluor@cell-green_1	5	6.58	0
HTS_STRONGACTIVE	genotoxic stress:tox21-p53-bla-p2_p53-agonist_1	16	21.05	0
HTS_STRONGACTIVE	counter screen (cell viability):tox21-p53-bla-p2_viability_1	14	18.42	0
TOXCAST_ACTIVE	OT_ER_ERbERb_1440	10	13.16	0
TOXCAST_ACTIVE	Tox21_TR_LUC_GH3_Antagonist	14	18.42	0
HTS_STRONGACTIVE	counter screen (cell viability):tox21-h2ax-cho-p2_viability_1	16	21.05	0
HTS_STRONGACTIVE	counter screen (cell viability):tox21-gh3-tre-antagonist-p1_viability_1	26	34.21	0
HTS_STRONGACTIVE	counter screen (cell viability):tox21-aromatase-p1_viability_1	23	30.26	0
HTS_STRONGACTIVE	counter screen (cell viability):tox21-pxr-p1_viability_1	21	27.63	0
HTS_STRONGACTIVE	genotoxic stress:tox21-p53-bla-p4_p53-agonist_1	13	17.11	0

**TABLE 3:** A summary of other significantly overrepresented annotations relating to sex steroid nuclear receptor signaling, metabolism, and TOP2A and GSTP1 as returned by Tox21Enricher-Shiny using bisphenols as the chemical input. For each row: the “Category” column refers to the source of the annotation; the “Term” column is the annotation name; the “Count” column refers to how many of the 76 input chemicals are associated with the given annotation; the “%” column is the “Count” column’s value converted to a percentage; and “*p*-value” column is the statistical significance of the annotation’s representation calculated using a modified Fisher’s exact test.

Table 3				
Category	Term	Count	%	*p*-value
CTD_CHEMICALS_GENES	ESR1	11	14.47	0
CTD_CHEMICALS_GENES	THRB	5	6.58	0.0006
TOXINS_TARGETS	CYP19A1	5	6.58	0.0022
CTD_CHEMICALS_GENES	ESR2	6	7.89	0.0028
TOXINS_TARGETS	PGR	5	6.58	0.0037
DRUGBANK_TARGETS	ESRRG	3	3.95	0.0037
TOXINS_TARGETS	NR3C1	5	6.58	0.0039
TOXINS_TARGETS	OPRM1	4	5.26	0.0043
CTD_CHEMICALS_GENES	PGR	5	6.58	0.0045
DRUGBANK_TARGETS	TOP2A	3	3.95	0.0052
TOXINS_TARGETS	AR	7	9.21	0.0054
CTD_CHEMICALS_GENES	CYP11A1	4	5.26	0.0071

#### 3.1.2 Example 2: enrichment of structural neighbors of a chemical not in Tox21 using structural similarity search

Polycyclic aromatic hydrocarbons are an ongoing environmental health challenge (Wikipedia, 2023). They arise from both anthropogenic and non-anthropogenic sources, primarily through combustion. A particular subclass of concern is the halogenated polycyclic aromatic hydrocarbons (Sun et al., 2013). Recent studies have identified a variety of these halogenated polycyclic aromatic hydrocarbons, and often there is little to no data on these compounds. Here, we used Tox21Enricher-Shiny to identify chemicals annotated in the Tox21Enricher database that are structurally similar to 9,10-Dibromoanthracene (SMILES: BrC1 = C2C = CC = CC2 = C(Br)C2 = CC = CC = C12) with a Tanimoto threshold of 30% similarity. We then used the application to determine the enriched biological and toxicological characteristics in structurally similar chemicals with the goal of hypothesizing the toxicological effects of 9,10-Dibromoanthracene. A total of 42 compounds were used for enrichment (see the PAH chemical list in the supplementary spreadsheet, “Tox21Enricher-Example Results and Chemical Lists”).

Enriched toxicological predictions from Leadscope indicated that the structural neighbors are enriched for genotoxic, carcinogenic, and reprotoxic properties, consistent with what would be expected of polycyclic aromatic hydrocarbons (Table 4).

**TABLE 4:** A summary of the most significantly overrepresented annotations relating to genotoxicity, carcinogenicity, and reproductive toxicity as returned by Tox21Enricher-Shiny using polycyclic aromatic hydrocarbons as the chemical input. For each row: the “Category” column refers to the source of the annotation; the “Term” column is the annotation name; the “Count” column refers to how many of the 42 input chemicals are associated with the given annotation; the “%” column is the “Count” column’s value converted to a percentage; and “*p*-value” column is the statistical significance of the annotation’s representation calculated using a modified Fisher’s exact test.

Table 4				
Category	Term	Count	%	*p*-value
LEADSCOPE_TOXICITY	Genetic Toxicity: *salmonella*	22	52.38	0
LEADSCOPE_TOXICITY	Genetic Toxicity: microbial	19	45.24	0
MULTICASE_TOX_PREDICTION	Sensory irritation, mouse	4	9.52	0
MULTICASE_TOX_PREDICTION	Draize eye irritation test, rabbit	8	19.05	0
LEADSCOPE_TOXICITY	Rodent Carcinogenicity: BALBc-3T3	25	59.52	0
MULTICASE_TOX_PREDICTION	Allergic contact dermatitis, guinea pig and human	9	21.43	0
LEADSCOPE_TOXICITY	Rodent Carcinogenicity: C3H10T1-2	4	9.52	0.0001
LEADSCOPE_TOXICITY	Genetic Toxicity: *In vitro* composite	15	35.71	0.0001
LEADSCOPE_TOXICITY	Genetic Toxicity: UDS rat hepatocytes	8	19.05	0.0003
LEADSCOPE_TOXICITY	Genetic Toxicity: *E. coli*	15	35.71	0.0004
MULTICASE_TOX_PREDICTION	RCA human acute liver damage	3	7.14	0.0013
LEADSCOPE_TOXICITY	Genetic Toxicity: *In vitro* chrom. ab. CHL	14	33.33	0.0016
LEADSCOPE_TOXICITY	Genetic Toxicity: UDS human lymphocytes	9	21.43	0.0016
LEADSCOPE_TOXICITY	Rodent Carcinogenicity: cell transformation	24	57.14	0.0027
LEADSCOPE_TOXICITY	Genetic Toxicity: UDS	10	23.81	0.0028
LEADSCOPE_TOXICITY	Rodent Carcinogenicity: SHE	24	57.14	0.003
LEADSCOPE_TOXICITY	Genetic Toxicity: SCE *in vitro* CHO	28	66.67	0.0035

The somewhat weaker enrichment of the DrugMatrix tissue toxicity and adverse effect annotations related to carcinogenic properties is consistent with chemicals that are genotoxic and are members of the polycyclic aromatic hydrocarbon class of chemicals (Table 5).

**TABLE 5:** A summary of some less-significantly overrepresented annotations relating to carcinogenicity as returned by Tox21Enricher-Shiny using polycyclic aromatic hydrocarbons as the chemical input. For each row: the “Category” column refers to the source of the annotation; the “Term” column is the annotation name; the “Count” column refers to how many of the 42 input chemicals are associated with the given annotation; the “%” column is the “Count” column’s value converted to a percentage; and “*p*-value” column is the statistical significance of the annotation’s representation calculated using a modified Fisher’s exact test.

Table 5			
Term	Count	%	*p*-value
DNA_Carcinogenicity	3	7.14	0.0136
Carcinogenicity	3	7.14	0.0276

The enrichment in the Toxin Target, CTD chemicals to genes/chemicals to pathway and Tox21 high-throughput screening (HTS) strong actives indicates that structurally similar chemicals to 9,10-Dibromoanthracene interact at the molecular level with known polycyclic aromatic hydrocarbons such as glycine N-methyltransferase (GNMT), aryl hydrocarbon receptor (AHR), cytochrome P450 family 1 subfamily A member 1 (CYP1A1), cytochrome P450 family 1 subfamily A member 2 (CYP1A2), epoxide hydrolase 1 (EPHX1), cytochrome P450 family 1 subfamily B member 1 (CYP1B1) and deoxyribonucleic acid (DNA) (related to genotoxicity consistent with the above Leadscope predictions) (Table 6). Consistent with the above results, the enriched CTD pathways suggest an association with drug/xenobiotic metabolism. Overall, the results suggest that 9,10-Dibromoanthracene is potentially genotoxic, carcinogenic, and toxic to the reproductive system. In addition, mechanistic enrichment analysis highlights possible interactions with DNA, AHR, and drug/xenobiotic pathways, further supporting the predicted adverse effects of this compound.

**TABLE 6:** A summary of other significantly overrepresented annotations relating to genotoxicity and drug/xenobiotic metabolism as returned by Tox21Enricher-Shiny using polycyclic aromatic hydrocarbons as the chemical input. For each row: the “Category” column refers to the source of the annotation; the “Term” column is the annotation name; the “Count” column refers to how many of the 42 input chemicals are associated with the given annotation; the “%” column is the “Count” column’s value converted to a percentage; and “*p*-value” column is the statistical significance of the annotation’s representation calculated using a modified Fisher’s exact test.

Table 6				
Category	Term	Count	%	*p*-value
TOXINS_TARGETS	GNMT	7	16.67	0
TOXINS_TARGETS	AHR	11	26.19	0
CTD_CHEMICALS_GENES	CYP1A	8	19.05	0
CTD_CHEMICALS_GENES	AHR	11	26.19	0
CTD_CHEMICALS_GENES	CYP1A2	11	26.19	0
CTD_CHEMICALS_GENES	CYP1B1	7	16.67	0
HTS_STRONGACTIVE	xenobiotic homeostasis:tox21-p450-1a2-p1_1a2-inhibitor_1	19	45.24	0
TOXINS_TARGETS	DNA	8	19.05	0.0001
CTD_CHEMICALS_GENES	EPHX1	5	11.90	0.0001
CTD_CHEMICALS_PATHWAYS	KEGG:hsa00982|Drug metabolism - cytochrome P450	6	14.29	0.003
CTD_CHEMICALS_GENES	CYP1A1	9	21.43	0.0042
HTS_STRONGACTIVE	sex hormone homeostasis:tox21-ar-mda-kb2-luc-antagonist-p2_ar-antagonist_1	8	19.05	0.005
HTS_STRONGACTIVE	xenobiotic homeostasis:tox21-ahr-p1_ahr-agonist_1	6	14.29	0.0057
TOXINS_TARGETS	CYP1B1	3	7.14	0.006
CTD_CHEMICALS_PATHWAYS	REACT:R-HSA-114608|Platelet degranulation	5	11.90	0.0064
CTD_CHEMICALS_PATHWAYS	REACT:R-HSA-211945|Phase 1 - Functionalization of compounds	6	14.29	0.0075
CTD_CHEMICALS_PATHWAYS	KEGG:hsa00980|Metabolism of xenobiotics by cytochrome P450	6	14.29	0.0078

#### 3.1.3 Example 3: enrichment of a set of chemicals with Tox21 biological assay fingerprints similar to DGRE

Diglycidyl resorcinol ether (DGRE) is used in the manufacturing of epoxy resins and is an International Agency for Research on Cancer (IARC) group 2B carcinogen (Wikipedia, 2022). We used this chemical in this example analysis because it has undergone in-depth toxicological characterization that can be used to compare the enriched annotations from Tox21Enricher-Shiny (CLH Report, 2018). We performed a Tox21 biological fingerprint correlation with the DGRE biological fingerprint and identified the top 25 most similar chemicals (i.e., those with similar patterns of biological activity in the Tox21 assays) using the area under the curve (AUC) metric and an n threshold of 100 in the NIEHS (2023) (see the DGRE chemical list in the supplementary spreadsheet, “Tox21Enricher-Example Results and Chemical Lists”) (Rstudio, 2023). One could evaluate a chemical with more limited data in Tox21 to infer its toxicological properties based on its biological fingerprint neighbors or develop bioactivity fingerprints from QSAR models developed on the Tox21 assays (Sedykh et al., 2021) to allow for immersion of the *in silico* fingerprint into the Tox21 biological activity landscape followed by annotation enrichment.

The top toxicological properties of the DGRE neighborhood chemical set include enrichment of genotoxicity, liver toxicity, developmental toxicity, and bone marrow toxicity (Table 7). Genotoxicity is a well-documented property of DGRE, and bone marrow toxicity has also been observed. It is quite common for genotoxic chemicals to be developmentally toxic; however, due to the high degree of point of contact toxicity, the systemic exposure to DGRE is likely limited, which blunts the developmental effects (i.e., dose-limiting toxicity is likely as the point of contact precludes high enough systemic exposure to produce developmental toxicity). Notably, many of the enriched toxicities here are related to genotoxic chemotherapeutics that are in the DGRE biological fingerprint neighborhood, which is a reasonable association given the strong genotoxic properties of DGRE. Also associated and noted in the Supplementary Data (DGRE results in the supplementary spreadsheet, “Tox21Enricher-Example Results and Chemical Lists”) is a weaker association with cancer. This weaker association may be because chemotherapeutics are often not assessed for their carcinogenicity, as in most cases, it is presumed based on their genotoxic properties. Hence, chemotherapeutics are underrepresented in training data for QSAR models such as those formulated by Leadscope.

**TABLE 7:** A summary of the most significantly overrepresented annotations relating to genotoxicity, liver toxicity, developmental toxicity, and bone marrow toxicity as returned by Tox21Enricher-Shiny using biologically similar chemicals to DGRE as the chemical input. For each row: the “Category” column refers to the source of the annotation; the “Term” column is the annotation name; the “Count” column refers to how many of the input chemicals are associated with the given annotation; the “%” column is the “Count” column’s value converted to a percentage; and “*p*-value” column is the statistical significance of the annotation’s representation calculated using a modified Fisher’s exact test.

Table 7				
Category	Term	Count	%	*p*-value
MULTICASE_TOX_PREDICTION	RCA human acute liver damage	19	76.00	0
LEADSCOPE_TOXICITY	Human Adverse Hepatobiliary Effects: bile duct	6	24.00	0
MULTICASE_TOX_PREDICTION	RCA fetal dysmorphogenesis, rodent	13	52.00	0
MULTICASE_TOX_PREDICTION	RCA human liver function (liver enzymes release blood test)	12	48.00	0
ADVERSE_EFFECT	BBM_Neutropenia	6	24.00	0.0001
ADVERSE_EFFECT	BBM_Thrombocytopenia	8	32.00	0.0001
ADVERSE_EFFECT	BBM_Anemia	7	28.00	0.0002
LEADSCOPE_TOXICITY	Human Adverse Hepatobiliary Effects: gall bladder	7	28.00	0.0005
ADVERSE_EFFECT	XXX_Infection	5	20.00	0.0006
LEADSCOPE_TOXICITY	Human Adverse Urinary Effects: nephropathy	8	32.00	0.001
KNOWN_TOXICITY	Blood and Bone Marrow Toxicity	5	20.00	0.0011
MULTICASE_TOX_PREDICTION	RCA fetal death, rodent	17	68.00	0.0014
ADVERSE_EFFECT	XXX_Mucositis	3	12.00	0.0028
ADVERSE_EFFECT	NEU_Peripheral Neuropathy	4	16.00	0.0037
ADVERSE_EFFECT	SKN_Alopecia/Hair Loss	5	20.00	0.0041
MULTICASE_TOX_PREDICTION	RCA fetal dysmorphogenesis, rat	7	28.00	0.0041
LEADSCOPE_TOXICITY	Genetic Toxicity: *drosophila* HT	6	24.00	0.0054
ADVERSE_EFFECT	NEU_Neurotoxicity	3	12.00	0.0055
LEADSCOPE_TOXICITY	Genetic Toxicity: *E. coli* w	7	28.00	0.0061
LEADSCOPE_TOXICITY	Developmental Toxicity: weight dec. rabbit	7	28.00	0.0063
ADVERSE_EFFECT	XXX_Fever	5	20.00	0.0075
LEADSCOPE_TOXICITY	Developmental Toxicity: retardation rodent	6	24.00	0.0093

The mechanistic annotations strongly indicate that the biological fingerprint neighbors of DGRE have genotoxic properties, specifically with enrichment of strong actives in the p53, dt40, and h2ax assays, and the enrichment of cell cycle and p53 signaling pathway annotations from the CTD along with parallel associations with genes involved in genotoxicity signaling (cyclin dependent kinase inhibitor 1A (CDKN1A), H2A.X Variant Histone (H2AX)) and apoptosis (caspase 8 (CASP8), poly(ADP-ribose) polymerase 1 (PARP1), caspase 9 (CASP9), B-cell lymphoma 2 (BCL2)) (Table 8). Also notable is the activity in the sex hormone assays, which are related to antagonism that is often associated with cytotoxicity, despite significant efforts to filter these responses out. Notably, there is no indication that DGRE is an endocrine disruptor. Some of the target and mechanistic associations include indications of interaction with DNA, effects on cytoskeleton (tubulin beta 1 class VI (TUBB1), multiple microtubule-associated proteins (MAPs)). These findings largely align with the known mechanistic properties of DGRE, i.e., genotoxicity.

**TABLE 8:** A summary of other significantly overrepresented annotations relating to genotoxicity as returned by Tox21Enricher-Shiny using biologically similar chemicals to DGRE as the chemical input. For each row: the “Category” column refers to the source of the annotation; the “Term” column is the annotation name; the “Count” column refers to how many of the input chemicals are associated with the given annotation; the “%” column is the “Count” column’s value converted to a percentage; and “*p*-value” column is the statistical significance of the annotation’s representation calculated using a modified Fisher’s exact test.

Table 8				
Category	Term	Count	%	*p*-value
HTS_STRONGACTIVE	genotoxic stress:tox21-p53-bla-p4_p53-agonist_1	16	64.00	0
HTS_STRONGACTIVE	cell viability:tox21-dt40-p1_viability@657_1	21	84.00	0
HTS_STRONGACTIVE	cell viability:tox21-dt40-p1_viability@100_1	21	84.00	0
HTS_STRONGACTIVE	cell viability:tox21-dt40-p1_viability@653_1	21	84.00	0
HTS_STRONGACTIVE	counter screen (cell viability):tox21-gh3-tre-antagonist-p1_viability_1	17	68.00	0
HTS_STRONGACTIVE	sex hormone homeostasis:tox21-ar-bla-antagonist-p1_ar-antagonist_1	12	48.00	0
HTS_STRONGACTIVE	thyroid homeostasis:tox21-gh3-tre-antagonist-p1_tr-antagonist_1	9	36.00	0
CTD_CHEMICALS_GENES	CASP8	5	20.00	0
CTD_CHEMICALS_GENES	CDKN1A	6	24.00	0.0001
MECHANISM	Incorporate into DNA/RNA/Protein	4	16.00	0.0001
DRUGBANK_TARGETS	TUBB1	3	12.00	0.0001
HTS_STRONGACTIVE	sex hormone homeostasis:tox21-er-luc-bg1-4e2-antagonist-p1_er-antagonist_1	7	28.00	0.0002
HTS_STRONGACTIVE	genotoxic stress:tox21-dt40-p1_dsb-inducer_1	5	20.00	0.0002
HTS_STRONGACTIVE	counter screen (cell viability):tox21-elg1-luc-agonist-p1_viability_1	7	28.00	0.001
HTS_STRONGACTIVE	xenobiotic homeostasis:tox21-car-antagonist-p1_car-antagonist_1	5	20.00	0.0043
CTD_CHEMICALS_GENES	PARP1	4	16.00	0.0048
CTD_CHEMICALS_GENES	H2AX	3	12.00	0.005
CTD_CHEMICALS_GENES	BCL2	5	20.00	0.006
CTD_CHEMICALS_PATHWAYS	KEGG:hsa04110|Cell cycle	5	20.00	0.0064
HTS_STRONGACTIVE	counter screen (cell viability):tox21-aromatase-p1_viability_1	9	36.00	0.0086
CTD_CHEMICALS_GENES	CETP	2	8.00	0.0088
CTD_CHEMICALS_PATHWAYS	KEGG:hsa04115|p53 signaling pathway	5	20.00	0.0103
CTD_CHEMICALS_GENES	CASP9	4	16.00	0.0108
TOXINS_TARGETS	MAP2	2	8.00	0.0114
TOXINS_TARGETS	MAP4	2	8.00	0.0114
CTD_CHEMICALS_GENES	CASP3	6	24.00	0.0121
MECHANISM	Inhibit DNA synthesis, repair, and function	4	16.00	0.0122
DRUGBANK_TARGETS	MAP4	2	8.00	0.0144
TOXINS_TARGETS	MAPT	2	8.00	0.0171
HTS_STRONGACTIVE	genotoxic stress:tox21-h2ax-cho-p2_gh2ax-inducer_1	5	20.00	0.019
TOXINS_TARGETS	DNA	4	16.00	0.0191
CTD_CHEMICALS_GENES	TP53	4	16.00	0.0203

Overall, the results of DGRE neighborhood enrichment analysis suggest that it is genotoxic and has effects on the bone marrow and liver. These associations are consistent with what has been observed with DGRE. There was also a weaker association with cancer, which is consistent with its genotoxic properties. One association that does not appear to be true is developmental toxicity, although a specific study assessing this endpoint was not identified in a literature search, and as noted above, the chemical properties may preclude it from being developmentally toxic. One association that was missed in this analysis is dermal irritancy, although there was a weak association for activation of nuclear factor erythroid 2-related factor 2 (Nrf2) in the skin (see the DGRE results in the supplementary spreadsheet, “Tox21Enricher-Example Results and Chemical Lists”), suggestive of irritancy and hypersensitivity.

### 3.2 Limitations

Tox21Enricher-Shiny’s limitations on its data and system architecture are discussed in this section.

#### 3.2.1 Biological and data limitations

Tox21Enricher-Shiny is contingent upon the annotations in its database component being accurate and current. Further, annotations are constrained to the chemical set used in Tox21; hence, the diversity of biological/toxicological annotations that can be enriched is inherently limited to what is represented in this chemical space. These annotations are sourced from a variety of providers, so the Tox21Enricher-Shiny system relies on those providers to make their data available. Tox21Enricher-Shiny’s result output should not be interpreted as a readout of real, pathological manifestation. As a predictive tool, it cannot make any definitive statements about how a chemical behaves and reacts; rather, Tox21Enricher-Shiny is only intended to suggest potential avenues to a user for further research about a chemical substance. Tox21Enricher-Shiny is intended to support research efforts and provide parallel, supplementary information. Tox21Enricher-Shiny also cannot accept all substances as input. Tox21Enricher-Shiny is only equipped to process the chemicals within the Tox21 dataset. The system will ignore user-supplied CASRNs that are not within Tox21, and it will only find similar chemicals for a user-provided SMILES or InChI string with respect to the Tox21 dataset. Tox21Enricher-Shiny may not correctly process chemicals that contain certain reactive functional groups (nitrile, isocyanate, aldehyde, epoxide) as these chemicals may react with proteins in cell-based Tox21 assays (Sushko et al., 2012). The Tox21Enricher-Shiny system relies on multiple packages and modules from other providers: the overall functionality and security of Tox21Enricher-Shiny may be influenced by the abilities, availabilities, and integrities of these tools.

#### 3.2.2 Architectural and software limitations

Tox21Enricher-Shiny was designed to have its database, API, and Shiny application components deployed from within separate Docker containers (Docker Inc., 2023a). Therefore, in a personal installation of Tox21Enricher-Shiny, it is not a requirement for all containers to be on the same machine. While this means that Tox21Enricher should technically be operational on any machine(s) that supports Docker, Tox21Enricher was only developed and tested on machines running Ubuntu 20.04 LTS (Canonical Ltd., 2023). The development machine for Tox21Enricher-Shiny used a 64-bit Linux architecture. The code for the API employs the use of parallel, multicore processing, which will not work as expected in Windows environments (Bengtsson, 2021). We do not guarantee that Tox21Enricher-Shiny will work as intended in other environments running architecturally different operating systems.

The throughput of Tox21Enricher-Shiny is dependent on the hardware running the system. Optimal performance may only be possible in environments that support parallel processing and can handle computationally expensive and memory-intensive procedures. The speed of processing a request may depend on the size of the input set(s) and/or the number of corresponding annotations. Tox21Enricher-Shiny officially supports only a maximum of sixteen separate input sets at a time to limit excessively long-running requests, but the upper limit may be configured lower in the API’s configuration settings. Since some requests are computationally expensive, we chose to limit input size to ensure the availability of the application’s functions. This limit may be changed in future releases if we continue testing using more robust systems. If a SMILES or InChI string input matches more than 1,500 CASRNs in the database, it will not be included in the enrichment analysis, and the user will be asked to refine their search criteria to reduce the size of the input chemical list.

A network connection is required for the components of the Tox21Enricher-Shiny system to interact and communicate with one another. If the Shiny application component is hosted as a public-facing web application, then an internet connection is necessary for users to be able to connect to the Shiny application through a browser or to connect directly to the API.

## 4 Discussion

Tox21Enricher-Shiny is currently hosted by the UND School of Medicine and Health Sciences as a public-facing tool.^4^ The application is also being used by the National Institute of Environmental Health Sciences (NIEHS) for further toxicological research on chemicals. Because of the application’s public-facing nature and accessible data and source code, other scientists and developers may also host their own instances of the Tox21Enricher-Shiny application with their own specific configurations. Tox21Enricher-Shiny may be used alone or in conjunction with read-across tools like GenRA to create comprehensive profiles, using annotations across different domains of information, for chemicals to be further studied.

### 4.1 Hosting and deployment

Tox21Enricher-Shiny can be deployed using any web server that supports R Shiny applications. UND is currently hosting the public Tox21Enricher-Shiny application through Docker Compose (Docker Inc., 2023b), while NIEHS hosts the Shiny application on an organizational Posit Connect server (Posit, 2023).

### 4.2 Scalability

Tox21Enricher-Shiny’s API uses multisession processing to perform some work in parallel. The number of resources available is dependent on the environment hosting the API, so the parallel computing power of the API component may scale up or down depending on the available resources. Environments with limited parallel computing resources may cause the API to perform work less efficiently or even sequentially with no parallelization.

### 4.3 Implications

Tox21Enricher-Shiny is a powerful tool for predicting possible behaviors of chemicals with sparse or insufficient literature. In this way, Tox21Enricher-Shiny may be used to identify chemicals in preparation for further toxicological research.

### 4.4 Future work

UND and NIEHS plan to continue developing Tox21Enricher-Shiny by improving existing mechanisms and expanding the system’s functionality and robustness. First, we plan to integrate Tox21Enricher-Shiny’s functionality, particularly the API component, with other NIEHS-developed applications for chemical research. We also plan to improve the clustering process’s speed and accuracy as well to improve users’ productivity. We may update the database component as new data become available. Finally, we plan to implement new data visualizations and a way to search for chemicals from provided SMILES or InChI strings based on similar results from predictive models.

## 5 Availability

The source code, documentation, database, and Docker containers for Tox21Enricher-Shiny may be accessed and downloaded from its GitHub repository ^5^.

## Figures and Tables

**Table:** 

**AHR**	aryl hydrocarbon receptor
**API**	application programming interface
**AR**	androgen receptor
**AUC**	area under the curve
**BCL2**	B-cell lymphoma 2
**CASP8**	caspase 8
**CASP9**	caspase 9
**CASRN**	Chemical Abstracts Service Registry Number
**CDKN1A**	cyclin dependent kinase inhibitor 1A
**COBRE**	Centers of Biomedical Research Excellence
**CTD**	Comparative Toxicogenomics Database
**CYP1A1**	cytochrome P450 family 1 subfamily A member 1
**CYP1A2**	cytochrome P450 family 1 subfamily A member 2
**CYP1B1**	cytochrome P450 family 1 subfamily B member 1
**CYP19A1**	cytochrome P450 family 19 subfamily A member 1
**DAVID**	Database for Annotation, Visualization, and Integrated Discovery
**DGRE**	Diglycidyl resorcinol ether
**DNA**	deoxyribonucleic acid
**DTT**	Division of Translational Toxicology
**EPA**	Environmental Protection Agency
**EPHX1**	epoxide hydrolase 1
**ER**	estrogen receptor
**ESR1**	estrogen receptor 1
**ESR2**	estrogen receptor 2
**GenRA**	Generalized Read-Across
**GNMT**	glycine N-methyltransferase
**GSTP1**	glutathione-S-transferase
**GUI**	graphical user interface
**HTS**	high-throughput screening
**HTTP**	Hypertext Transfer Protocol
**H2AX**	H2A.X Variant Histone
**IARC**	International Union of Pure and Applied Chemistry
**JSME**	JavaScript Molecular Editor
**LTS**	long-term support
**MAP**	Environmental Health Sciences
**NIGMS**	National Institute of General Medical Sciences
**NQO1**	NAD(P)H quinone dehydrogenase 1
**Nrf2**	nuclear factor erythroid 2-related factor 2
**PARP1**	poly(ADP-ribose) polymerase 1
**PGR**	progesterone receptor
**PNG**	Portable Network Graphics
**QSAR**	quantitative structure-activity relationship
**SAR**	structure-activity relationship
**SMILES**	simplified molecular-input line-entry system
**TOP2A**	topoisomerase
**ToxRefDB**	Toxicity Reference Database
**Tox21**	Toxicology Testing in the 21st Century
**TUBB1**	tubulin beta 1 class VI
**T3DB**	Toxin and Toxin Target Database
**UND**	University of North Dakota
**UUID**	universally unique identifier

## Data Availability

The source code, documentation, database, and Docker containers for Tox21Enricher-Shiny may be accessed and downloaded from the following link - http://hurlab.med.und.edu/tox21enricher_db.tar.gz.

## Footnotes

1
http://hurlab.med.und.edu/Tox21Enricher/.

2
https://github.com/hurlab/tox21enricher.

3
https://github.com/hurlab/tox21enricher.

4
http://hurlab.med.und.edu/Tox21Enricher/.

5
https://github.com/hurlab/tox21enricher.
