# Corrigendum: Tox21Enricher-Shiny: an R Shiny application for toxicity functional annotation analysis

**DOI:** 10.3389/ftox.2023.1278066

**Published:** 2023-08-24

**Authors:** Parker Combs, Jeremy Erickson, Jui-Hua Hsieh, Kai Guo, Sue Nolte, Charles Schmitt, Scott Auerbach, Junguk Hur

**Affiliations:** ^1^ Department of Biomedical Sciences, School of Medicine and Health Sciences, University of North Dakota, Grand Forks, ND, United States; ^2^ National Institute of Environmental Health Sciences (NIH), Durham, NC, United States; ^3^ Michigan Medicine, University of Michigan Health, Ann Arbor, MI, United States

**Keywords:** toxicity analysis, Tox21, enrichment analysis, R shiny, web-based application

In the published article, the citation for National Center for Advancing Translational Sciences was displayed as “National Center for Advancing Translational Sciences, 2018”. The correct citation is in **Introduction**, Paragraph 4 and should read:

“Tox21Enricher-Shiny contains annotations from various sources for 8,948 chemicals derived from the Tox21 10K library, which is a set of 10,000 chemicals currently being studied by the National Center for Advancing Translational Sciences (NCATS) (National Center for Advancing Translational Sciences, 2022). The scope of the annotations in the database is limited to data that could be collected from studying these chemicals.”

In the published article, the reference for Hur et al. (2018) was incorrectly omitted. It should be “Hur J., Danes, L., Hsieh, J.-H., McGregor, B., Krout, D., and Auerbach, S. (2018). Tox21 Enricher: Web-based Chemical/Biological Functional Annotation Analysis Tool Based on Tox21 Toxicity Screening Platform. Molecular Informatics 2018 May; 37(5): e1700129. doi: 10.1002/minf.201700129”.

In the published article, the reference for Sushko et al. (2012) was incorrectly omitted. It should be “Sushko, I., Salmina, E., Potemkin, V. A., Poda, G., and Tetko, I. V. (2012). ToxAlerts: a Web server of structural alerts for toxic chemicals and compounds with potential adverse reactions. J Chem Inf Model. 2012 Aug 27; 52(8): 2310-6. doi: 10.1021/ci300245q”.

In the published article, there was an error in the reference for National Center for Advancing Translational Sciences (2022). The corrected reference appears below.

“National Center for Advancing Translational Sciences (2022). About Tox21. Available at: https://ncats.nih.gov/tox21 (Accessed August 14 2023)”.

The authors apologize for these errors and state that they do not change the scientific conclusions of the article in any way. The original article has been updated.
