# Supplementary material for: Tox21Enricher-Shiny: an R Shiny application for toxicity functional annotation analysis
Source: Front Toxicol. 2023 Jun 27;5:1147608. doi: 10.3389/ftox.2023.1147608 (PMC10333489; doi:10.3389/ftox.2023.1147608)
Supplement: Supplementary file 1 [file DataSheet2.PDF]

Tox21Enricher-Shiny
Setup Guide

(Last Updated May 25, 2023)

Shiny Instance: http://hurlab.med.und.edu/tox21enricher

API Instance: http://hurlab.med.und.edu/tox21enricher-api

GitHub Repository: https://github.com/hurlab/tox21enricher

Copyright 2023 University of North Dakota

All rights reserved.

Junguk Hur, Ph.D.

Department of Biomedical Sciences

University of North Dakota, School of Medicine and Health Sciences

Grand Forks, North Dakota 58202, USA

Email: InformaticsTools@gmail.com

http://hurlab.med.und.edu

Table of Contents

I. Introduction to Tox21Enricher-Shiny 3

II. Quick Start 4

III. Advanced Setup 5

III.1. Tox21Enricher-Shiny Source Code 5

III.2. Application Configuration 5

III.2.a. Plumber API Configuration 5

III.2.b. Shiny Application Configuration 6

III.3. Database Access and Specifications 7

III.3.a. Getting Copies of the Databases 7

III.3.b. Restoring the Databases from the Backups 7

III.3.c. Database Tables and Functions 8

III.4. Rebuilding the Docker Images 12

III.5. Launching the Docker Images with Docker Compose 12

IV. Other Utilities 13

IV.1. API Demos 13

V. Common Issues 14

I. Introduction to Tox21Enricher-Shiny

Humans are exposed to tens of thousands of chemicals that are used in daily life, some at levels that may pose a health risk. There is limited toxicological information for many of these chemicals, which makes risk assessment difficult or impossible. The United States Toxicology Testing in the 21st Century (Tox21) program was established to develop more efficient and human-relevant toxicity assessment methods. The Tox21 program is currently screening a set of over 10,000 chemicals, the Tox21 10K library, using quantitative high-throughput screening (qHTS) of assays that measure effects on toxicity pathways. To date, more than 70 assays have yielded >12 million concentration-response curves by Tox21 researchers. To efficiently apply these data for identifying potential hazardous compounds and for informing further toxicological testing, the United States National Toxicology Program (NTP) has developed several web applications (Tox21 Toolbox: https://ntp.niehs.nih.gov/whatwestudy/tox21/toolbox/index.html), including tools for data visualization (Tox21 Curve Browser) and exploration (Tox21 Activity Profiler).

One critical usage of this dataset is to perform chemical-relational analysis based on the patterns of activity across the Tox21 assays and then to use nearest neighbor-based prediction to infer the toxicological properties of untested chemicals via their association with tested chemicals. One approach to inferring the specific properties is to perform chemical annotation enrichment of chemical neighborhoods.

Here, we present Tox21Enricher-Shiny, a web-based chemical annotation enrichment tool for Tox21 assay data built using the R Shiny framework. Tox21Enricher-Shiny identifies significantly over-represented chemical biological annotations among sets of chemicals (neighborhoods), which facilitates the identification of the toxicological properties and mechanisms in the chemical sets.

II. Quick Start

Docker (https://www.docker.com/) is used to provide a quick way to set up a locally hosted instance of Tox21Enricher-Shiny. There are three official images, **tox21enricher/db**, **tox21enricher/api**, and **tox21enricher/shiny**, for the Postgres database server, Plumber API, and Shiny application components, respectively. Tox21Enricher-Shiny uses Docker Compose (https://docs.docker.com/compose/compose-file/) to launch containers for all three images on their own internal network. The following instructions will explain how to set up an instance of Tox21Enricher-Shiny in full on your own machine.

1. Make sure you have Docker and Docker Compose installed on the host machine as per the instructions at https://docs.docker.com/engine/install/ for the appropriate OS on the host machine.
2. Download the zipped archive containing the Docker images and Docker Compose configuration file at http://hurlab.med.und.edu/tox21enricher-docker.zip.
3. Unzip the archive's files (docker-compose.yml, tox21enricher_image_db.tar, tox21enricher_image_api.tar, and tox21enricher_image_shiny.tar) to a directory of your choosing.
4. From within that directory, run the commands:
    - `docker load --input tox21enricher_image_db.tar`
    - `docker load --input tox21enricher_image_api.tar`
    - `docker load --input tox21enricher_image_shiny.tar`

    To restore the images from the .tar files.
5. Also from within that directory (in the same directory as **docker-compose.yml**) and after all the images are loaded, run the command: `docker-compose up`.
6. Wait until the API container displays the messages:
    - `Running plumber API at http://0.0.0.0:<port>`
    - `Running swagger Docs at http://127.0.0.1:<port>/__docs__/`

    After these messages appear, you should be able to safely connect to the API and Shiny app (by default, the database's port is not exposed to the host for security reasons). By default, the API is accessible on port 9990 and the Shiny app is accessible on port 9991 from the host machine. The ports to use may be configured in the **docker-compose.yml** file.
7. Use the command `docker-compose down` to stop the running containers when done.

Please refer to the comments in the docker-compose file for additional configuration information.

For advanced usage, configuration, and setup information, please refer to the rest of this document.

III. Advanced Setup

The recommended method for running and deploying Tox21Enricher-Shiny is to use Docker as outlined in **Section II: Quick Start**. This section will detail how to acquire the source code for Tox21Enricher-Shiny, configure a local instance of the application, and rebuild the Docker images using user-supplied settings.

III.1. Tox21Enricher-Shiny Source Code

The Tox21Enricher-Shiny code for both the server-side and client-side applications may be found at https://github.com/hurlab/tox21enricher. Assuming you have cloned the repository into a folder called "/home/user/tox21enricher/" on your machine, the Postgres database code can be found in `/home/user/tox21enricher/database/`, the Plumber API code can be found in `/home/user/tox21enricher/tox21enricher-api/`, and the Shiny application code can be found in `/home/user/tox21enricher/tox21enricher/`.

III.2. Application Configuration

III.2.a. Plumber API Configuration

Assuming you have cloned the code to `/home/user/tox21enricher/`, the API's configuration file can be found at `/home/user/tox21enricher/tox21enricher-api/config.yml`. This configuration file has two namespaces, `tox21enricher` and `tox21enricher-queue`, for the **tox21enricher database** and the **tox21queue database**, respectively. The importance of each of the variables in this configuration file is explained below:

"tox21enricher" namespace

- **host** – The name or IP address of the machine where the database is hosted. This should be set to the host name of the Docker container hosting the Postgres database ("tox21enricher-db" by default).
- **uid** – The username of the Postgres user that owns the tox21enricher database ("tox21enricher_user" by default).
- **pwd** – The password of the Postgres user that owns the tox21enricher database ("tox21enricher_pass" by default).
- **port** – The port that the database server is listening on **on the internal Docker network, not the port mapped on the host**. By default, this should be set to `"5432"`.
- **database** – The name of the database on the Postgres server. This should be `"tox21enricher"`.
- **appdir** – The location of the server-side project files. This should be wherever you cloned the repository to (i.e., `"/home/user/tox21enricher/"`).
- **indir** – The location of the directory where input files from requests should be stored.
- **outdir** – The location of the directory where result (output) files from requests should be stored.
- **cores** – The number of cores available to use when using multicore processing (Unavailable on Windows). This is initially set to 4.
- **apiHost** – The hostname or IP address where the API will be hosted.

- **apiPort** – The port on the host where the API will be hosted.
- **apiSecure** – `true` or `false` value. Should be `true` if the host where the API is located is using HTTPS and `false` if it is using HTTP.

“tox21enricher-queue” namespace

- **host** – The name or IP address of the machine where the database is hosted. This should be set to the host name of the Docker container hosting the Postgres database (“tox21enricher-db” by default).
- **uid** – The username of the Postgres user that owns the tox21queue database (“tox21enricher_queuemanager” by default).
- **pwd** – The password of the Postgres user that owns the tox21queue database (“tox21enricher_pass2” by default).
- **port** – The port that the database server is listening on **on the internal Docker network, not the port mapped on the host**. By default, this should be set to `“5432”`.
- **database** – The name of the database on the Postgres server. This should be `“tox21queue”`.
- **inputMax** – The number of concurrent sets that may be accepted as part of a single request. This value *n* must be 1 <= *n* <= 16. If More than 16 sets are specified, the application will set this value to 16. If less than 1 set is specified, the application will set this value to 1.
- **pvaluedisplay** – The numeric value that determines how significant an annotation’s p-value must be to be included in the result files. This will default to 0.2 if an invalid value is supplied.
- **archivedir** – The location of the directory where the zipped archives from old requests should be saved after they can no longer be accessed from the application. If blank, results will be deleted instead of archived.
- **cleanupTime** – The number of hours a transaction will be kept alive in the queue before being canceled and marked for deletion. Also, the number of hours until the expiration of a cookie created on the client-side that stores a request’s UUID.
- **deleteTime** – The number of days (>= 1) before an old transaction is archived. If set to -1, old request results will always be kept indefinitely and archival is disabled.

III.2.b. Shiny Application Configuration

Assuming you have cloned the code to `/home/user/tox21enricher/`, the API’s configuration file can be found at `/home/user/tox21enricher/tox21enricher/config.yml`. The importance of each of the variables in this configuration file is explained below:

- **host** – The name or IP address of the machine where the Tox21Enricher-Shiny Plumber API is hosted. This should be set to the host name of the Docker container hosting the Plumber API (“tox21enricher-api” by default).
- **port** – The port that the API is listening on **on the internal Docker network, not the port mapped on the host**. By default, this should be set to `“8000”`.
- **secure** – Should be `true` or `false` (without any quotation marks). Set to `true` if the API address uses HTTPS. Set to `false` if it uses HTTP instead.

III.3. Database Access and Specifications

Tox21Enricher-Shiny provides two databases on the same Postgres server: **tox21enricher** and **tox21queue**. **tox21enricher** stores read-only data relating to the chemicals in the Tox21 dataset, while **tox21queue** contains a few writable tables that store information about submitted requests. This section will detail the specifications of the Tox21Enricher-Shiny database.

III.3.a. Getting Copies of the Databases

The **tox21enricher** database may be downloaded in its entirety (with all tables filled and all necessary functions included) here: http://hurlab.med.und.edu/tox21enricher_db.tar.gz. The **tox21queue** database with all tables created (but unpopulated) may be downloaded here: http://hurlab.med.und.edu/tox21enricher_queue.sql. These data are also linked to on the project's GitHub repository: https://github.com/hurlab/tox21enricher.

III.3.b. Restoring the Databases from the Backups

To restore the **tox21enricher** and **tox21queue** database from scratch, follow these instructions:

1. Install a **clean** instance of Postgres following the instructions at https://www.postgresql.org/download/. The minimum supported version is **Postgres 11.2**.
2. Create a new directory to house the data for the database. For example, this directory can be called "tox21enricher-postgres."
3. Use Postgres's **initdb** utility (https://www.postgresql.org/docs/current/app-initdb.html) to create a database cluster in the new directory. The command in this example would be `initdb -D /home/user/tox21enricher-postgres/`.
4. Start the Postgres server using the **pg_ctl** command and specifying the previously initialized database cluster directory: `pg_ctl -D /home/user/tox21enricher-postgres/ -l /home/user/tox21enricher-postgres/logfile start`.
5. Create the necessary databases and users using the following commands:
   - `psql -c "CREATE ROLE <user who has read permission on the tox21enricher database> WITH LOGIN PASSWORD '<password for user>';" postgres`
   - `psql -c "CREATE ROLE <user who has read and write permission on the tox21queue database> WITH LOGIN PASSWORD '<password for user>';" postgres`
   - `createdb tox21enricher`
   - `createdb tox21queue`

   In this example, the user that has read permission on the "tox21enricher" database will be called "tox21enricher_user" and the user that has read and write permission on the "tox21queue" database will be called "tox21enricher_queuemanager."
6. Download the database dumps from the links provided above. Decompress the **tox21enricher_db.tar.gz** file to retrieve the **tox21enricher_db.sql** file.
7. Restore the database content to the currently empty databases:
   - psql -d tox21enricher -a -f tox21enricher_db.sql
   - psql -d tox21enricher -a -f tox21enricher_queue.sql

8. Finally, set the correct permissions for the “tox21enricher_user” and “tox21enricher_queuemanager” users using the following Postgres commands:
   - `psql tox21enricher -c "REVOKE ALL ON ALL TABLES IN SCHEMA public FROM tox21enricher_user;"`
   - `psql tox21queue -c "REVOKE ALL ON ALL TABLES IN SCHEMA public FROM tox21enricher_user;"`
   - `psql tox21enricher -c "REVOKE ALL ON ALL TABLES IN SCHEMA public FROM tox21enricher_queuemanager;"`
   - `psql tox21queue -c "REVOKE ALL ON ALL TABLES IN SCHEMA public FROM tox21enricher_queuemanager;"`
   - `psql tox21enricher -c "GRANT SELECT ON ALL TABLES IN SCHEMA public TO tox21enricher_user;"`
   - `psql tox21queue -c "GRANT SELECT, INSERT, UPDATE ON ALL TABLES IN SCHEMA public TO tox21enricher_queuemanager;"`

III.3.c. Database Tables and Functions

“tox21enricher” database tables and functions

9. annotation_class

**Name**	**Type**	**Constraints**
annoclassid	INTEGER	NOT NULL, AUTO INCREMENT, PRIMARY KEY
annoclassname	VARCHAR(50)	NOT NULL
firsttermid	INTEGER	NOT NULL
lasttermid	INTEGER	NOT NULL
numberoftermids	INTEGER	NOT NULL
baseurl	VARCHAR(255)	NOT NULL
annotype	VARCHAR(100)	
annogroovyclassname	VARCHAR(100)	
annodesc	VARCHAR(7000)	
networkcolor	VARCHAR(11)	

10. annotation_detail

**Name**	**Type**	**Constraints**
annotermid	INTEGER	NOT NULL, AUTO INCREMENT, PRIMARY KEY
annoclassid	INTEGER	NOT NULL, FOREIGN KEY REFERENCES annotation_class(annoclassid)
annoterm	VARCHAR(7000)	NOT NULL

11. annoterm_pairwise

**Name**	**Type**	**Constraints**
pairwise	INTEGER	NOT NULL, AUTO INCREMENT, PRIMARY KEY
term1uid	INTEGER	NOT NULL

term2uid	INTEGER	NOT NULL
term1size	INTEGER	NOT NULL
term2size	INTEGER	NOT NULL
common	INTEGER	NOT NULL
union	INTEGER	NOT NULL
jaccardindex	DOUBLE	NOT NULL
pvalue	DOUBLE	NOT NULL
qvalue	DOUBLE	NOT NULL

12. chemical_detail

Name	Type	Constraints
casrnuid	INTEGER	NOT NULL, AUTO INCREMENT, PRIMARY KEY
casrn	VARCHAR(15)	NOT NULL
testsubstance_chemname	VARCHAR(500)	NOT NULL
molecular_formular	VARCHAR(50)	
iupac_name	VARCHAR(1000)	
inchis	VARCHAR(1000)	
inchikey	VARCHAR(30)	
smiles	VARCHAR(600)	
smiles_qsar_ready	VARCHAR(600)	
dtxsid	VARCHAR(20)	
stoichiometric_ratio	VARCHAR(10)	
cid	VARCHAR(20)	
mol_formula	VARCHAR(200)	
mol_weight	DOUBLE	
dtxrid	VARCHAR(20)	

13. fps_2

Name	Type	Constraints
casrn	VARCHAR(15)	
torsionbv	BFP	
mfp2	BFP	
ffp2	BFP	

14. mols_2

Name	Type	Constraints
casrn	VARCHAR(15)	
m	MOL	
cyanide	INTEGER	
isocyanate	INTEGER	
aldehyde	INTEGER	
epoxide	INTEGER	

15. term2casrn_mapping

Name	Type	Constraints
term2casrnmappinguid	INTEGER	NOT NULL, AUTO INCREMENT, PRIMARY KEY

annotermid	INTEGER	NOT NULL
annoclassid	INTEGER	NOT NULL
casrnuid	INTEGER	NOT NULL

16. get_mfp2_neighbors (this is a function added on top of those in the RDKit Postgres cartridge by default)

**Description**
CREATE OR REPLACE FUNCTION public.get_mfp2_neighbors(smiles text)<br>RETURNS TABLE(casrn character varying, m character varying, similarity double precision, cyanide integer, isocyanate integer, aldehyde integer, epoxide integer)<br>LANGUAGE sql<br>STABLE<br>AS $function$<br>select casrn,mol_to_smiles(m)::character varying,tanimoto_sml(morganbv_fp(mol_from_smiles($1::cstring)),mfp2) as similarity,cyanide,isocyanate,aldehyde,epoxide<br>from fps_2 join mols_2 using (casrn)<br>where morganbv_fp(mol_from_smiles($1::cstring))%mfp2<br>order by morganbv_fp(mol_from_smiles($1::cstring))<%>mfp2;<br>$function$

“tox21queue” database tables

17. queue

Name	Type	Constraints
mode	VARCHAR(15)	
uuid	VARCHAR(50)	NOT NULL, PRIMARY KEY
annoselectstr	VARCHAR(1500)	
cutoff	INTEGER	
finished	INTEGER	DEFAULT 0
index	INTEGER	NOT NULL, AUTO INCREMENT
error	VARCHAR(500)	
cancel	INTEGER	NOT NULL, DEFAULT 0
lock	INTEGER	NOT NULL, DEFAULT 0

18. status

Name	Type	Constraints
step	INTEGER	NOT NULL, DEFAULT 0
uuid	VARCHAR(50)	NOT NULL, FOREIGN KEY REFERENCES queue(uuid), ON DELETE CASCADE
setname	VARCHAR(500)	

19. transaction

Name	Type	Constraints
original_mode	VARCHAR(15)	
mode	VARCHAR(15)	NOT NULL
uuid	VARCHAR(40)	NOT NULL, PRIMARY KEY
annotation_selection_string	VARCHAR(1000)	
cutoff	INTEGER	NOT NULL
input	VARCHAR	
original_names	VARCHAR	
reenrich	VARCHAR	
colors	VARCHAR	
timestamp_posted	VARCHAR(50)	NOT NULL, DEFAULT CURRENT_DATE
timestamp_started	VARCHAR(50)	DEFAULT ’not started’
timestamp_finished	VARCHAR(50)	DEFAULT ’incomplete’
cancel	INTEGER	NOT NULL, DEFAULT 0
reenrich_flag	INTEGER	NOT NULL, DEFAULT 0
casrn_box	VARCHAR	
delete	INTEGER	NOT NULL, DEFAULT 0
pvalue	TEXT	

III.4. Rebuilding the Docker Images

To rebuild the Docker images from source, consult the file `docker_build_instructions.txt` in the main project directory. Open a terminal and navigate to the main project directory and run each of the following commands to build the corresponding image:

Build the tox21enricher/db (Postgres server) image

```
cd database && docker build -t tox21enricher/db -f Dockerfile . --
progress=plain --no-cache \
--build-arg USER_USERNAME='user' \
--build-arg USER_PASSWORD='password' \
--build-arg POSTGRES_DATABASE_USERNAME='tox21enricher_user' \
--build-arg POSTGRES_DATABASE_PASSWORD='tox21enricher_pass' \
--build-arg POSTGRES_QUEUE_USERNAME='tox21enricher_queuemanager' \
--build-arg POSTGRES_QUEUE_PASSWORD='tox21enricher_pass2'
```

You must define certain arguments in the command to properly build the image:

- USER_USERNAME: the username of the user who will own and run the Postgres server.
- USER_PASSWORD: the password of the user defined above.
- POSTGRES_DATABASE_USERNAME: the username of the Postgres user who will have read-only access on the “tox21enricher” database ONLY.
- POSTGRES_DATABASE_PASSWORD: the password of the user defined above.
- POSTGRES_QUEUE_USERNAME: the username of the Postgres user who will have read/write access on the “tox21queue” database ONLY.
- POSTGRES_ QUEUE _PASSWORD: the password of the user defined above.

Build the tox21enricher/api (Plumber API) image

```
cd tox21enricher-api && docker build -t tox21enricher/api -f
Dockerfile . --progress=plain --no-cache \
--build-arg USER_USERNAME='user' \
--build-arg USER_PASSWORD='password'
```

You must define certain arguments in the command to properly build the image:

- USER_USERNAME: the username of the user who will own and run the API process.
- USER_PASSWORD: the password of the user defined above.

Build the tox21enricher/shiny (Shiny server) image

```
cd tox21enricher && docker build -t tox21enricher/shiny -f
Dockerfile . --progress=plain --no-cache
```

III.5. Launching the Docker Images with Docker Compose

After the images are finished rebuilding, open a terminal and navigate to the main project directory (the same directory as `docker-compose.yml`) and run the command `docker-compose up` to start containers using the newly built images. Use the command `docker-compose down` to stop the running containers when done.

IV. Other Utilities

IV.1. API Demos

Examples for API usage relating to the headless submission of requests, can be found in the <project_root>/demos/ directory.

V. Common Issues

Both the client and server code for Tox21Enricher-Shiny are still in continued development, so there are likely issues that may arise when trying to either perform certain tasks or set up the application. Known bugs in the code and planned features are documented on the project's GitHub repository at: https://github.com/hurlab/tox21enricher/issues.

END OF THE SETUP GUIDE
