# Supplementary material for: Tox21Enricher-Shiny: an R Shiny application for toxicity functional annotation analysis
Source: Front Toxicol. 2023 Jun 27;5:1147608. doi: 10.3389/ftox.2023.1147608 (PMC10333489; doi:10.3389/ftox.2023.1147608)
Supplement: Supplementary file 3 [file Table1.docx]

**Annotation Category Table**. An overview of each annotation category in the Tox21Enricher database. This table includes each category’s name, a brief description, the number of annotations in the category, and the number of chemicals that are associated with at least one annotation in the category.

| **Category Name** | **Description** | **Number of annotations** | **Number of chemicals** | **Source** |
| --- | --- | --- | --- | --- |
| ACTIVITY_CLASS | Chemical/drug to activity class (e.g., DNA damage, anti-inflammatory) annotations provided by the DrugMatrix Database. | 84 | 612 | (U.S. Department of Health and Human Services, n.d.) |
| ADVERSE_EFFECT | Chemical/drug to adverse effect annotations curated from the PDR, drug package inserts, or primary literature. Effects are scored based on severity (SSS for severe to S for moderate/minimal severity) and frequency of occurrence (1-most frequent, 2-less common, 3-rare). | 1,248 | 941 | (U.S. Department of Health and Human Services, n.d.) |
| CTD_CHEMICALS_DISEASES | Chemical/drug to disease (e.g., prostate cancer) associations that have been curated in the Comparative Toxicogenomics Database through literature review. Some associations in this set have been inferred based on bridging linkages through genes. | 4,057 | 2,583 | (MDI Biological Laboratory and NC State University, 2022a) |
| CTD_CHEMICALS_GENES | Chemical/drug to gene (e.g., ESR1) associations that have been curated in the Comparative Toxicogenomics Database through literature review. | 3,438 | 2,273 | (MDI Biological Laboratory and NC State University, 2022c) |
| CTD_CHEMICALS_GOENRICH_CELLCOMP | Chemical/drug to gene ontology cellular component (e.g., mitochondria) associations that have been curated in the Comparative Toxicogenomics Database through literature review. Some associations in this set have been inferred based on bridging linkages through genes. | 393 | 1,103 | (MDI Biological Laboratory and NC State University, 2022b) |
| CTD_CHEMICALS_GOENRICH_MOLFUNCT | Chemical/drug to gene ontology molecular function (e.g., catalysis) associations that have been curated in the Comparative Toxicogenomics Database through literature review. Some associations in this set have been inferred based on bridging linkages through genes. | 460 | 1,090 | (MDI Biological Laboratory and NC State University, 2022b) |
| CTD_CHEMICALS_ PATHWAYS | Chemical/drug to molecular pathway (e.g., P53 pathway) associations that have been curated in the Comparative Toxicogenomics Database through literature review. | 805 | 939 | (MDI Biological Laboratory and NC State University, 2022d) |
| CTD_GOFAT_BIOPROCESS | Chemical/drug to gene ontology biological processes (e.g., xenobiotic metabolism) associations that have been curated in the Comparative Toxicogenomics Database through literature review. Some associations in this set have been inferred based on bridging linkages through genes. Only includes terms found in the GO Fat subset. | 7,356 | 1,850 | (MDI Biological Laboratory and NC State University, 2022b) |
| CTD_GOSLIM_BIOPROCESS | Chemical/drug to gene ontology biological processes (e.g., xenobiotic metabolism) associations that have been curated in the Comparative Toxicogenomics Database through literature review. Some associations in this set have been inferred based on bridging linkages through genes. Only includes terms found in the GO Slim subset. | 68 | 1,491 | (MDI Biological Laboratory and NC State University, 2022b) |
| DRUGBANK_ATC | Chemical/drug to ATC (anatomic therapeutic category) code annotations maintained by WHO and extracted from DrugBank. | 514 | 1,261 | (DrugBank, n.d.a) |
| DRUGBANK_ATC_CODE | Chemical/drug to ATC (anatomic therapeutic category) code annotations maintained by WHO and extracted from DrugBank. | 321 | 648 | (DrugBank, n.d.a) |
| DRUGBANK_CARRIERS | Chemical/drug to blood carrier protein (e.g., ALB) annotations provided by DrugBank. | 39 | 267 | (DrugBank, n.d.b) |
| DRUGBANK_ENZYMES | Chemical/drug to metabolic enzyme (e.g., CYP1A1) annotations provided by DrugBank. | 262 | 793 | (DrugBank, n.d.b) |
| DRUGBANK_TARGETS | Chemical to molecular target (e.g., ESR1) annotations derived from DrugBank. | 1,806 | 1,249 | (DrugBank, n.d.b) |
| DRUGBANK_TRANSPORTERS | Chemical/drug to transporter (e.g., ABCC1) annotations provided by DrugBank. | 179 | 455 | (DrugBank, n.d.b) |
| HTS_ACTIVE | Chemical to in vitro biological activity annotations from the Tox21 assays. | 209 | 6,512 | (National Library of Medicine, n.d.c) |
| HTS_STRONGACTIVE | Chemical to in vitro biological activity annotations from the Tox21 assays. | 209 | 4,870 | (National Library of Medicine, n.d.c) |
| INDICATION | Chemical/drug to clinical indication annotations provided by the DrugMatrix Database. | 979 | 1,313 | (U.S. Department of Health and Human Services, n.d.) |
| KNOWN_TOXICITY | Chemical/drug to known toxicological effects (e.g., liver toxicity) provided by the DrugMatrix Database. | 22 | 678 | (U.S. Department of Health and Human Services, n.d.) |
| LEADSCOPE_TOXICITY | Chemical to SAR-based predicted toxicological effects provided by Leadscope SAR models. | 103 | 7,259 | (Instem, 2022) |
| MECH_LEVEL_1 | Chemical/drug to mechanistic (e.g., androgen receptor antagonist) annotations that are the least specific of all three levels provided by the DrugMatrix Database. | 89 | 507 | (U.S. Department of Health and Human Services, n.d.) |
| MECH_LEVEL_2 | Chemical/drug to mechanistic (e.g., androgen receptor antagonist) annotations that are more specific than level 1 and less specific than level 3. | 147 | 524 | (U.S. Department of Health and Human Services, n.d.) |
| MECH_LEVEL_3 | Chemical/drug to mechanistic (e.g., androgen receptor antagonist) annotations that are more specific than level 2. | 170 | 539 | (U.S. Department of Health and Human Services, n.d.) |
| MECHANISM | Chemical/drug to mechanism (e.g., lipid metabolism, block neuronal transmission) annotations provided by the DrugMatrix Database. | 68 | 1,530 | (U.S. Department of Health and Human Services, n.d.) |
| MESH | Chemical to MeSH Chemical and Drug [D] annotations; specifically, the terminal branch MeSH terms in the “D” subset provided by PubChem. | 2,444 | 5,355 | (National Library of Medicine, n.d.a) |
| MODE_CLASS | Chemical/drug to mode class (e.g., enzyme inhibitor, receptor agonist) annotations provided by the DrugMatrix Database. | 25 | 1,476 | (U.S. Department of Health and Human Services, n.d.) |
| MULTICASE_TOX_PREDICTION | Chemical to SAR-based predicted toxicological effects provided by Multicase SAR models. | 115 | 7,882 | (MultiCASE, 2022) |
| PHARMACTIONLIST | Chemical to pharmacological action terms derived from the USNLM. Provided by the DrugMatrix Database. | 468 | 2,720 | (National Library of Medicine, n.d.b) |
| PRODUCT_CLASS | Chemical/drug to product class (i.e., target system, e.g., endocrine, CNS) annotations derived from the DrugMatrix Database. | 23 | 1,571 | (U.S. Department of Health and Human Services, n.d.) |
| STRUCTURE_ACTIVITY | Chemical/drug to structural activity class (i.e., molecular target associated with therapeutic effects) annotations derived from the DrugMatrix Database. | 297 | 1,210 | (U.S. Department of Health and Human Services, n.d.) |
| TA_LEVEL_1 | Chemical to drug/therapeutic use category (e.g., infectious disease). TA_LEVEL_1 is the highest (i.e., most abstract) drug use category. Provided by DrugMatrix. | 34 | 543 | (U.S. Department of Health and Human Services, n.d.) |
| TA_LEVEL_2 | Chemical to drug use/therapeutic category that is more specific than TA_LEVEL_1 (e.g., solid tumors). Provided by the DrugMatrix Database. | 144 | 506 | (U.S. Department of Health and Human Services, n.d.) |
| TA_LEVEL_3 | Chemical to drug use/therapeutic category that is more specific than TA_LEVEL_2 (e.g., prostate cancer). Provided by the DrugMatrix Database. | 189 | 492 | (U.S. Department of Health and Human Services, n.d.) |
| THERAPEUTIC_CLASS | Chemical/Drug to therapeutic class (e.g., antidiabetic agents) annotations derived from the DrugMatrix database. | 99 | 1,452 | (U.S. Department of Health and Human Services, n.d.) |
| TISSUE_TOXICITY | Chemical to target tissue toxicity annotations derived from the DrugMatrix Database. | 198 | 678 | (U.S. Department of Health and Human Services, n.d.) |
| TOXCAST_ACTIVE | Chemical to in vitro biological activity annotations from the EPA ToxCast data. | 725 | 1,694 | (EPA, 2022) |
| TOXINS_TARGETS | Chemical to molecular target annotation. The annotations in this set associate chemicals with their known molecular targets/molecular initiators (e.g., receptors) that have been curated in the Toxin and Toxin Target Database. | 1,630 | 1,220 | (Lim et al., 2010; Wishart et al., 2015) |
| TOXPRINT_STRUCTURE | Chemical to substructure/chemotype annotations. | 626 | 8,195 | (Altamira LLC and Molecular Networks GmbH, 2013) |
| TOXREFDB | Chemical to in vivo toxicological effect annotations. The annotations in the set associate chemicals with their toxicological effects in guideline toxicity studies. | 4238 | 883 | (USEPA/CompTox-ToxRefDB, 2019) |

**References**

Altamira LLC and Molecular Networks GmbH. (2013). ToxPrint | ChemoTypes. Available at: https://toxprint.org/#GenericStructuralFragments [Accessed July 19, 2022].

DrugBank. (n.d.a). ATC Classification. Available at: https://go.drugbank.com/atc [Accessed June 11, 2023].

DrugBank. (n.d.b). Peptides. Available at: https://go.drugbank.com/categories/DBCAT000052 [Accessed June 11, 2023].

EPA. (2022). CompTox Chemicals Dashboard. Available at: https://comptox.epa.gov/dashboard [Accessed July 19, 2022].

Instem. (2022). Computational Toxicology. Available at: https://www.instem.com/solutions/insilico/computational-toxicology.php [Accessed July 19, 2022].

Lim, E., Pon, A., Djoumbou, Y., Knox, C., Shrivastava, S., Guo, A. C., Neveu, V., and Wishart, D. S. (2010). T3DB: a comprehensively annotated database of common toxins and their targets. Nucleic Acids Research, 38(Database issue), D781-6. doi: 10.1093/nar/gkp934.

MDI Biological Laboratory and NC State University. (2022a). Diseases | CTD. Available at: https://ctdbase.org/voc.go?type=disease [Accessed July 19, 2022].

MDI Biological Laboratory and NC State University. (2022b). Gene Ontology | CTD. Available at: https://ctdbase.org/voc.go?type=go [Accessed July 19, 2022].

MDI Biological Laboratory and NC State University. (2022c). Gene Query | CTD. Available at: https://ctdbase.org/query.go?type=gene [Accessed July 19, 2022].

MDI Biological Laboratory and NC State University. (2022d). Pathways | CTD. Available at: https://ctdbase.org/voc.go?type=pathway [Accessed July 19, 2022].

MultiCASE. (2022). Toxicity Assessments. Available at: http://www.multicase.com/case-ultra-models [Accessed July 19, 2022].

National Library of Medicine. (n.d.a). Home - MeSH - NCBI. Available at: https://www.ncbi.nlm.nih.gov/mesh/?term= [Accessed July 18, 2022].

National Library of Medicine. (n.d.b). Pharmacologic Actions - MeSH - NCBI. Available at: https://www.ncbi.nlm.nih.gov/mesh/?term=actions%2C+pharmacologic [Accessed July 18, 2022].

National Library of Medicine. (n.d.c). PubChem BioAssay. Available at: https://www.ncbi.nlm.nih.gov/pcassay?term=%22Tox21%22%5BSourceName%5D%20hasnohold%5Bfilt%5D [Accessed July 18, 2022].

U.S. Department of Health and Human Services. (n.d.). DrugMatrix/ToxFX.Available at: https://ntp.niehs.nih.gov/data/drugmatrix/ [Accessed July 18, 2022].

USEPA/CompTox-ToxRefDB. (2019). Available at: https://github.com/USEPA/CompTox-ToxRefDB [Accessed July 19, 2022].

Wishart, D., Arndt, D., Pon, A., Sajed, T., Guo, A. C., Djoumbou, Y., Knox, C., Wilson, M., Liang, Y., Grant, J., Liu, Y., Goldansaz, S. A., and Rappaport, S. M. (2015). T3DB: the toxic exposome database. Nucleic Acids Research, 43(Database issue), D928-34. doi: 10.1093/nar/gku1004.
