# Supplementary material for: Tox21Enricher-Shiny: an R Shiny application for toxicity functional annotation analysis
Source: Front Toxicol. 2023 Jun 27;5:1147608. doi: 10.3389/ftox.2023.1147608 (PMC10333489; doi:10.3389/ftox.2023.1147608)
Supplement: Supplementary file 4 [file Table2.docx]

**Software Dependency Table**. A table of the software used in the development of Tox21Enricher-Shiny. This table includes the name, type, version used, purpose, and source of each software.

| Software Name | Type | Version | Function | Reference |
| --- | --- | --- | --- | --- |
| base | R package | 3.6.3 | Dependency of loaded packages | (R Core Team, 2020a) |
| config | R package | 0.3.1 | Persistent settings for database connections, file paths, and other options | (config, n.d.) |
| datasets | R package | 3.6.3 | Dependency of loaded packages | (R Core Team, 2020b) |
| data.table | R package | 1.14.0 | Efficient processing of large data frames | (Dowle and Srinivasan, 2021) |
| DBI | R package | 1.1.1 | Database connection | (R Special Interest Group on Databases (R-SIG-DB) et al., 2021) |
| Docker | Virtualization software | 23.0.1 | Saving environments to be used in deployment | (Docker Inc., 2023a) |
| Docker Compose | Virtualization software | 1.25.0 | Allows saved Docker images to be run together and connect to each other | (Docker Inc., 2023b) |
| dplyr | R package | 1.0.5 | Convenient data processing | (Wickham et al., 2021a) |
| DT | R package | 0.18 | Presenting data tables | (DT, 2021) |
| forcats | R package | 0.5.1 | Dependency of loaded packages | (Wickham, 2021) |
| futile.logger | R package | 1.4.3 | Dependency of loaded packages | (Rowe, 2016) |
| future | R package | 1.31.0 | Asynchronous processing | (Bengtsson, 2021) |
| ggplot2 | R package | 3.3.3 | Data visualization | (Wickham, 2016) |
| graphics | R package | 3.6.3 | Dependency of loaded packages | (R Core Team, 2020c) |
| grDevices | R package | 3.6.3 | Dependency of loaded packages | (R Core Team, 2020d) |
| grid | R package | 3.6.3 | Dependency of loaded packages | (R Core Team, 2020e) |
| heatmaply | R package | 1.4.2 | Clustered heatmaps with dendrograms | (Galili et al., 2017) |
| httr | R package | 1.4.2 | Communication between Shiny application and API | (Wickham, 2020) |
| igraph | R package | 1.2.6 | Data visualization | (Csárdi and Nepusz, 2006) |
| igraphdata | R package | 1.0.1 | Data visualization | (Csárdi and Nepusz, 2006) |
| methods | R package | 3.6.3 | Dependency of loaded packages | (R Core Team, 2020f) |
| parallel | R package | 3.6.3 | Asynchronous processing | (Bengtsson, 2021) |
| plotly | R package | 4.9.3 | Data visualization | (Sievert, 2020) |
| plumber | R package | 1.1.0 | API development and deployment | (Schloerke and Allen, 2021) |
| plyr | R package | 1.8.6 | Convenient data processing | (Wickham, 2011) |
| pool | R package | 0.1.6 | Database connection | (Cheng and Borges, 2021) |
| PostgreSQL | Database management software | 12.2 | Manage the Tox21Enricher-Shiny database | (The PostgreSQL Global Development Group, 2020) |
| promises | R package | 1.2.0.1 | Asynchronous processing | (Cheng, 2021) |
| purrr | R package | 0.3.4 | Convenient data processing | (Wickham and Henry, 2020) |
| R | Programming language | 3.6.3 “Holding the Windsock” | Programming language | (The R Foundation, 2020) |
| rclipboard | R package | 0.1.3 | Provides copy-to-clipboard functionality in the Shiny application | (rclipboard, n.d.) |
| RDKit database cartridge | Postgres extension | 3.8 | Provides cheminformatics utilities for use within the PostgreSQL database | (Landrum, 2021) |
| readr | R package | 1.4.0 | Dependency of loaded packages | (Wickham et al., 2020) |
| rjson | R package | 0.2.20 | Communication between Shiny application and API | (Couture-Beil, n.d.) |
| RPostgres | R package | 1.3.3 | Database connection | (Wickham et al., 2021b) |
| shiny | R package | 1.6.0 | Frontend development | (Posit, 2021) |
| shinyBS | R package | 0.61 | Frontend development | (Bailey, 2015) |
| shinycssloaders | R package | 1.0.0 | Frontend development | (Attali, 2020a) |
| shinyjs | R package | 2.0.0 | Allows JavaScript to be used in Shiny | (Attali, 2020b) |
| stats | R package | 3.6.3 | Dependency of loaded packages | (R Core Team, 2020g) |
| stringr | R package | 1.4.0 | Utilities for processing strings | (Wickham, 2019) |
| tibble | R package | 3.1.0 | Dependency of loaded packages | (Müller and Wickham, 2021) |
| tidyr | R package | 1.1.3 | Dependency of loaded packages | (Wickham et al., 2023) |
| tidyverse | R package | 1.3.0 | Convenient data processing | (Tidyverse, n.d.) |
| Ubuntu | Operating system | 20.04.2 LTS (Focal Fossa) Beta (64-bit Linux) | Recommended operating system | (Canonical Ltd., n.d.) |
| utils | R package | 3.6.3 | Various utilities | (R Core Team, 2020h) |
| uuid | R package | 0.1.4 | Generation of unique identifiers for requests | (Urbanek and Ts’o, 2020) |
| VennDiagram | R package | 1.6.20 | Data visualization | (Chen and Boutros, 2018) |
| visNetwork | R package | 2.0.9 | Data visualization | (DataStorm, 2021) |

**References**

Attali, D. (2020a). shinycssloaders. Available at: https://github.com/daattali/shinycssloaders [Accessed April 12, 2023].

Attali, D. (2020b). shinyjs. Available at: https://deanattali.com/shinyjs/ [Accessed April 12, 2023].

Bailey, E. (2015). shinyBS. Available at: https://ebailey78.github.io/shinyBS/ [Accessed April 12, 2023].

Bengtsson, H. (2021). A Unifying Framework for Parallel and Distributed Processing in R using Futures. R J 13, 208. doi: 10.32614/RJ-2021-048.

Canonical Ltd. (n.d.). Ubuntu 20.04.2 LTS (Focal Fossa) Beta. Available at: https://old-releases.ubuntu.com/releases/20.04.2/ [Accessed April 12, 2023].

Chen, H., and Boutros, P. (2018). VennDiagram: Generate High-Resolution Venn and Euler Plots. Available at: https://cran.r-project.org/package=VennDiagram [Accessed April 12, 2023].

Cheng, J. (2021). Promises. Available at: https://rstudio.github.io/promises/ [Accessed April 12, 2023].

Cheng, J., and Borges, B. (2021). pool: Object Pooling. Available at: https://github.com/rstudio/pool [Accessed April 12, 2023].

config (n.d.). Available at: https://github.com/rstudio/config [Accessed April 12, 2023].

Couture-Beil, A. (n.d.). rjson. Available at: https://github.com/alexcb/rjson [Accessed April 12, 2023].

Csárdi, G., and Nepusz, T. (2006). The igraph software package for complex network research. InterJournal Complex Systems, 1695–1695.

DataStorm (2021). visNetwork, an R package for interactive network visualization. Available at: https://datastorm-open.github.io/visNetwork/ [Accessed April 12, 2023].

Docker Inc. (2023a). Docker. Available at: https://www.docker.com/ [Accessed April 12, 2023].

Docker Inc. (2023b). Docker Compose overview. Available at: https://docs.docker.com/compose/ [Accessed April 12, 2023].

Dowle, M., and Srinivasan, A. (2021). data.table. Available at: https://github.com/Rdatatable/data.table [Accessed April 12, 2023].

DT (2021). Available at: https://github.com/rstudio/DT [Accessed April 12, 2023].

Galili, T., O’Callaghan, A., Sidi, J., and Sievert, C. (2017). heatmaply: an R package for creating interactive cluster heatmaps for online publishing. Bioinformatics 2018;34(9), 1600-1602. doi: 10.1093/bioinformatics/btx657.

Landrum, G. (2021). The RDKit database cartridge. Available at: https://www.rdkit.org/docs/Cartridge.html [Accessed April 12, 2023].

Müller, K., and Wickham, H. (2021). tibble: Simple Data Frames. Available at: https://tibble.tidyverse.org/ [Accessed April 13, 2023].

Posit (2021). Shiny. Available at: https://shiny.rstudio.com/ [Accessed April 12, 2023].

R Core Team (2020a). base. Available at: https://cran.r-project.org/bin/windows/base/old/3.6.3/ [Accessed April 13, 2023].

R Core Team (2020b). datasets. Available at: https://cran.r-project.org/bin/windows/base/old/3.6.3/ [Accessed April 13, 2023].

R Core Team (2020c). graphics. Available at: https://cran.r-project.org/bin/windows/base/old/3.6.3/ [Accessed April 13, 2023].

R Core Team (2020d). grDevices. Available at: https://cran.r-project.org/bin/windows/base/old/3.6.3/ [Accessed April 13, 2023].

R Core Team (2020e). grid. Available at: https://cran.r-project.org/bin/windows/base/old/3.6.3/ [Accessed April 13, 2023].

R Core Team (2020f). methods. Available at: https://cran.r-project.org/bin/windows/base/old/3.6.3/ [Accessed April 13, 2023].

R Core Team (2020g). stats. Available at: https://cran.r-project.org/bin/windows/base/old/3.6.3/ [Accessed April 13, 2023].

R Core Team (2020h). utils. Available at: https://cran.r-project.org/bin/windows/base/old/3.6.3/ [Accessed April 13, 2023].

R Special Interest Group on Databases (R-SIG-DB), Wickham, H., Müller, K., and R Consortium (2021). DBI. Available at: https://github.com/r-dbi/DBvI [Accessed April 12, 2023].

rclipboard (n.d.). Available at: https://github.com/sbihorel/rclipboard/ [Accessed April 12, 2023].

Rowe, B. L. Y. (2016). futile.logger: A Logging Utility for R. Available at: https://cran.r-project.org/package=futile.logger [Accessed April 13, 2023].

Schloerke, B., and Allen, J. (2021). plumber: An API Generator for R. Available at: https://www.rplumber.io [Accessed April 12, 2023].

Sievert, C. (2020). Interactive Web-Based Data Visualization with R, plotly, and shiny. Chapman and Hall/CRC Available at: https://plotly-r.com [Accessed April 12, 2023].

The PostgreSQL Global Development Group (2020). PostgreSQL: The World’s Most Advanced Open Source Relational Database. Available at: https://www.postgresql.org/ [Accessed April 12, 2023].

The R Foundation (2020). The R Project for Statistical Computing. Available at: https://www.r-project.org/ [Accessed April 12, 2023].

Tidyverse (n.d.). Available at: https://www.tidyverse.org/ [Accessed April 12, 2023].

Urbanek, S., and Ts’o, T. (2020). uuid - UUID manipulation. Available at: https://www.rforge.net/uuid/ [Accessed April 12, 2023].

Wickham, H. (2021). forcats: Tools for Working with Categorical Variables (Factors). Available at: https://forcats.tidyverse.org/ [Accessed April 13, 2023].

Wickham, H. (2016). ggplot2: Elegant Graphics for Data Analysis. Springer-Verlag New York.

Wickham, H. (2020). httr: Tools for Working with URLs and HTTP. Available at: https://httr.r-lib.org/ [Accessed April 12, 2023].

Wickham, H. (2019). stringr: Simple, Consistent Wrappers for Common String Operations. Available at: https://stringr.tidyverse.org [Accessed April 12, 2023].

Wickham, H. (2011). The Split-Apply-Combine Strategy for Data Analysis. J Stat Softw 40, 1–29. Available at: https://www.jstatsoft.org/v40/i01/ [Accessed April 12, 2023].

Wickham, H., François, R., Henry, L., Müller, K., and Vaughan, D. (2021a). dplyr: A Grammar of Data Manipulation. Available at: https://dplyr.tidyverse.org [Accessed April 12, 2023].

Wickham, H., and Henry, L. (2020). purrr: Functional Programming Tools. Available at: https://purrr.tidyverse.org/ [Accessed April 13, 2023].

Wickham, H., Hester, J., and Bryan, J. (2020). readr: Read Rectangular Text Data. Available at: https://readr.tidyverse.org/ [Accessed April 13, 2023].

Wickham, H., Ooms, J., Müller, K., and R Consortium (2021b). RPostgres. Available at: https://rpostgres.r-dbi.org [Accessed April 12, 2023].

Wickham H., Vaughan D., and Girlich M. (2023). tidyr: Tidy Messy Data. Available at: https://tidyr.tidyverse.org, https://github.com/tidyverse/tidyr [Accessed June 12, 2023].
