# Supplementary material for: Tox21Enricher-Shiny: an R Shiny application for toxicity functional annotation analysis
Source: Front Toxicol. 2023 Jun 27;5:1147608. doi: 10.3389/ftox.2023.1147608 (PMC10333489; doi:10.3389/ftox.2023.1147608)
Supplement: Supplementary file 5 [file DataSheet1.PDF]

Tox21Enricher-Shiny User's Manual

(Last Updated June 2, 2023)

Shiny Instance: http://hurlab.med.und.edu/tox21enricher

API Instance: http://hurlab.med.und.edu/tox21enricher-api

GitHub Repository: https://github.com/hurlab/tox21enricher

Copyright 2023 University of North Dakota

All rights reserved.

Junguk Hur, Ph.D.

Department of Biomedical Sciences

University of North Dakota, School of Medicine and Health Sciences

Grand Forks, North Dakota 58202, USA

Email: InformaticsTools@gmail.com

http://hurlab.med.und.edu

Table of Contents

I. Introduction to Tox21Enricher-Shiny 2
II. Landing Page 3
II.1. Sidebar 5
II.2. Selecting Annotation Categories 6
II.3. Selecting the Enrichment Mode and Input Type 7
II.3.a. Enrich From User Provided CASRN List 7
II.3.b. Enrich From Chemicals With Shared Substructures 7
II.3.c. Enrich From Chemicals With Structural Similarity 9
II.3.d. View Annotations for Tox21 Chemicals 10
II.4. Selecting Enrichment Cutoff Value 10
II.5. Selecting the P-Value Calculation Method 11
II.6. Performing Enrichment 11
III. Waiting Page 12
IV. Results Page 14
IV.1. Enrichment Results 14
IV.1.a. Result Files 14
IV.1.b. Result Chemicals 15
IV.1.c. Download Results Button 17
IV.1.d. Adjust Network Node Cutoff & Perform Re-enrichment 17
IV.1.e. Chart and Cluster Heatmaps 18
IV.1.f. Chart and Cluster Networks 19
IV.1.g. Significant P-value per Annotation Category Bar Graphs 21
IV.1.h. Start Over Button 21
IV.2. Annotation Results 22
IV.2.a. Result Files 22
IV.2.b. Download Results Button 22
V. Viewing Results From Previous Requests 23
VI. Headless Mode 25
VII. Common Issues & Questions 27
VIII. Resources 28
IX. References 29

I. Introduction to Tox21Enricher-Shiny

Humans are exposed to tens of thousands of chemicals that are used in daily life, some at levels that may pose a health risk. There is limited toxicological information for many of these chemicals, which makes risk assessment difficult or impossible. The United States Toxicology Testing in the 21st Century (Tox21) program was established to develop more efficient and human-relevant toxicity assessment methods. The Tox21 program is currently screening a set of over 10,000 chemicals, the Tox21 10K library, using quantitative high-throughput screening (qHTS) of assays that measure effects on toxicity pathways. To date, more than 70 assays have yielded >12 million concentration-response curves by Tox21 researchers. To efficiently apply these data for identifying potential hazardous compounds and for informing further toxicological testing, the United States National Toxicology Program (NTP) has developed several web applications (Tox21 Toolbox: https://ntp.niehs.nih.gov/whatwestudy/tox21/toolbox/index.html), including tools for data visualization (Tox21 Curve Browser) and exploration (Tox21 Activity Profiler).

One critical usage of this dataset is to perform chemical-relational analysis based on the patterns of activity across the Tox21 assays and then to use nearest neighbor-based prediction to infer the toxicological properties of untested chemicals via their association with tested chemicals. One approach to inferring the specific properties is to perform chemical annotation enrichment of chemical neighborhoods.

Here, we present Tox21Enricher-Shiny, a web-based chemical annotation enrichment tool for Tox21 assay data built using the R Shiny framework. Tox21Enricher-Shiny identifies significantly over-represented chemical biological annotations among sets of chemicals (neighborhoods), which facilitates the identification of the toxicological properties and mechanisms in the chemical sets.

II. Landing Page

Upon first using Tox21Enricher-Shiny via an internet browser or directly through the client application, you will see the landing page.

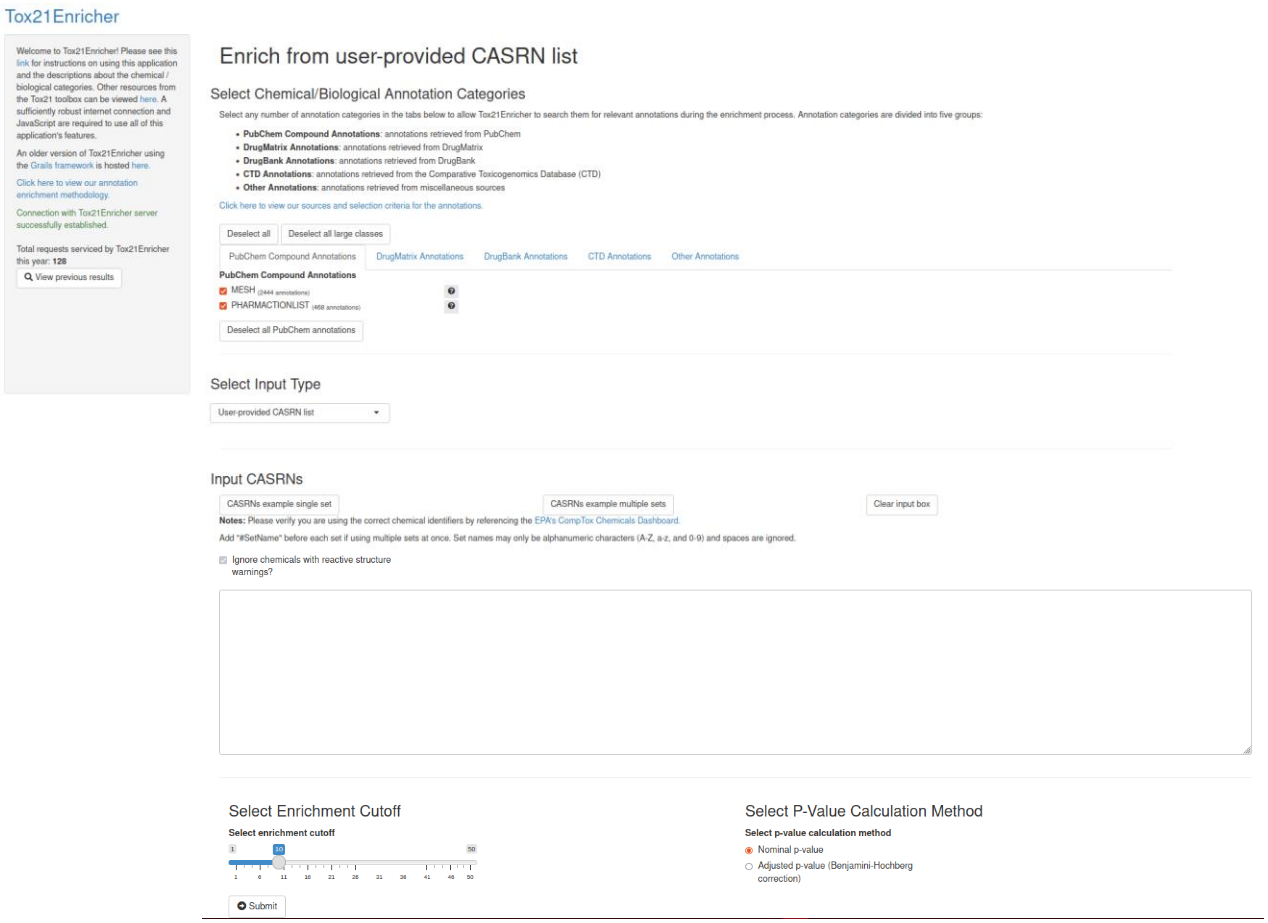

The various utilities and options on the landing page will be discussed at length in this section. At any point during application usage, you can click the title at the top-left that reads “Tox21Enricher” to be taken back to the landing page.

II.1. Sidebar

The sidebar of the application displays helpful information.

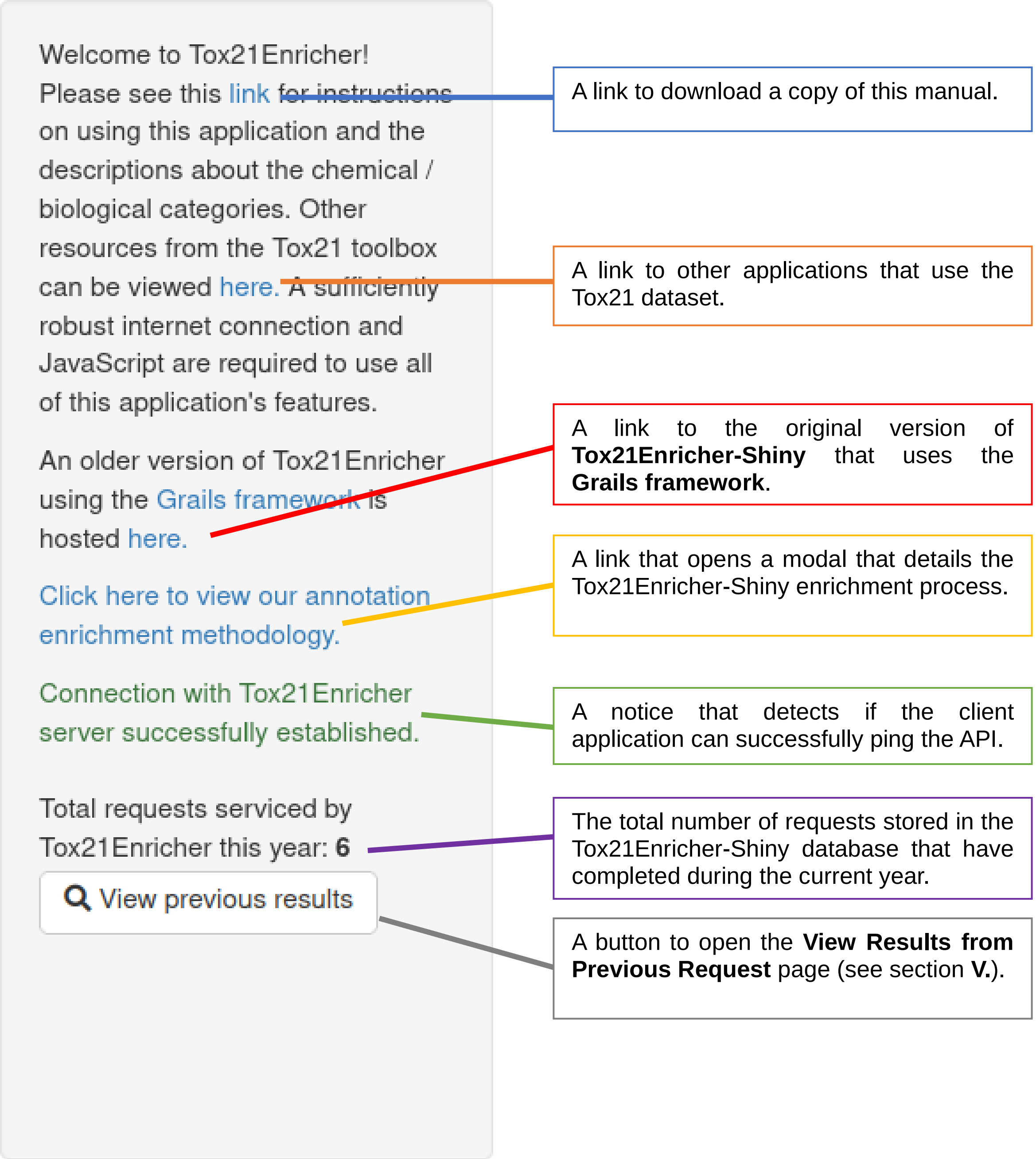

II.2. Selecting Annotation Categories

The top of the landing page displays lists of annotation categories, separated into five category types: **PubChem Compound Annotations**, **DrugMatrix Annotations**, **DrugBank Annotations**, **CTD Annotations**, and **Other Annotations**. By default, the **PubChem Compound Annotations** tab is selected. Additional information about each data source consulted and how annotations were retrieved can be accessed by clicking the “Click here to view our sources and selection criteria for the annotations” link. You can click on the tabs to view each tab’s corresponding annotation categories. Each category name has a checkbox to its left that, when checked, means that the application will attempt to find matching annotations from the given category. The application will ignore any potential results from categories left unchecked. By default, all categories are checked except for the CTD_GOFAT_BIOPROCESS category: using this category can potentially slow down enrichment by a large amount, so it is not recommended to be used in most scenarios. Each category name has a question mark button to its right that, when hovered over, displays additional information about each category. If no categories are selected, the application defaults to just using all the annotation categories (except for CTD_GOFAT_BIOPROCESS).

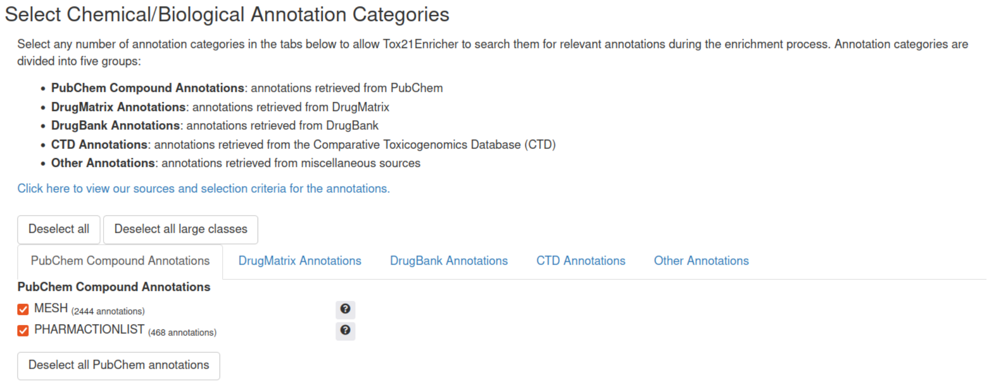

Clicking the **Deselect all/Select all** button will either deselect or select all the annotation categories across all tabs. Note that clicking the **Select all** button will select the CTD_GOFAT_BIOPROCESS category too.

Each tab also has a corresponding button to deselect or select all the annotation categories within the tab. An example can be seen in the image above: clicking the **Deselect all DrugBank annotations** button will deselect only the annotations in the **DrugBank Annotations** tab. Note that clicking the **CTD** tab’s corresponding **Select all CTD annotations** button will select the CTD_GOFAT_BIOPROCESS category too.

The number of annotations for a class is displayed next to the class name. The classes are ordered so that those with the most annotations are at the top. Clicking the **Deselect all large classes** button will deselect all classes with more than 1000 annotations.

II.3. Selecting the Enrichment Mode and Input Type

There are three enrichment analysis modes: User-provided CASRN list, Chemicals with shared substructures, and Chemicals with structural similarity. A fourth mode, View annotations for Tox21 chemicals, does not perform enrichment but rather retrieves annotations from the Tox21Enricher-Shiny database that correspond to the input chemicals.

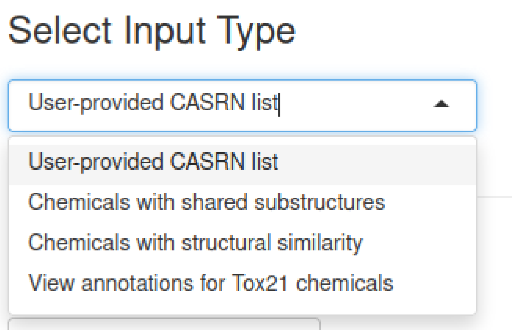

II.3.a. Enrich From User Provided CASRN List

This enrichment analysis type accepts CASRN strings as input with one CASRN per line. Multiple sets may be independently enriched at the same time. To enrich multiple sets, separate lines of CASRN strings with **unique** set names of the form "#SetName" with a pound symbol (#) before the name. Only alphanumeric characters (A-Z, a-z and 0-9) may be used in set names. If you choose to provide names for input sets, **all** sets must be assigned a name. Spaces in set names or CASRN strings are ignored, and extra newline characters between lines are also ignored. If no set names are provided, all lines of the input box will be treated as one set and will be assigned the default name of "Set1."

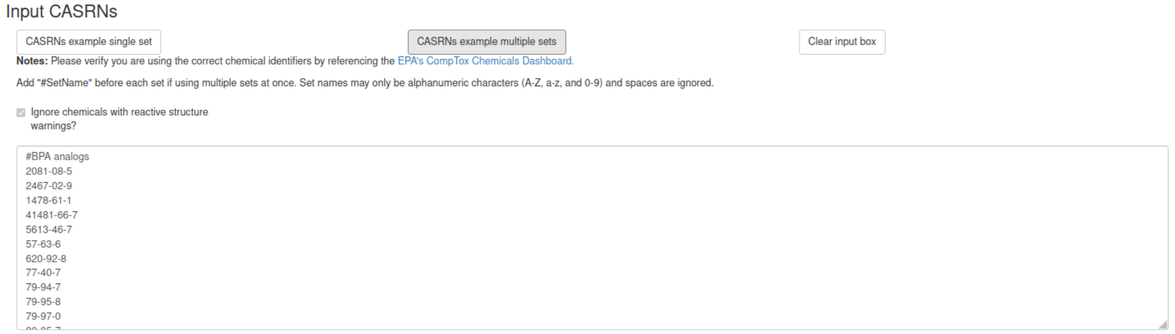

Before performing enrichment from a user provided CASRN list, please cross-reference your CASRN inputs with those in the EPA's CompTox Chemicals Dashboard (https://comptox.epa.gov/dashboard) to ensure accuracy during the enrichment analysis. Clicking the **CASRNs example single set** button will fill the input box with one, unnamed set of CASRNs. Clicking the **CASRNs example multiple sets** button will fill the input box with three sets of CASRNs. Clicking the **Clear input box** button will reset the input box to a blank state.

II.3.b. Enrich From Chemicals With Shared Substructures

This enrichment analysis type accepts either SMILES or InChI strings (one per line). Each string is treated as its own data set and will be enriched independently of any other sets provided. SMILES and InChI strings may be mixed: you may perform enrichment with a SMILES string on one line and an InChI string on another. Clicking the **SMILES/InChI**

example set button will fill the input box with three chemicals: two SMILES strings and one InChI string. Clicking the **Clear input box** button will reset the input box to a blank state.

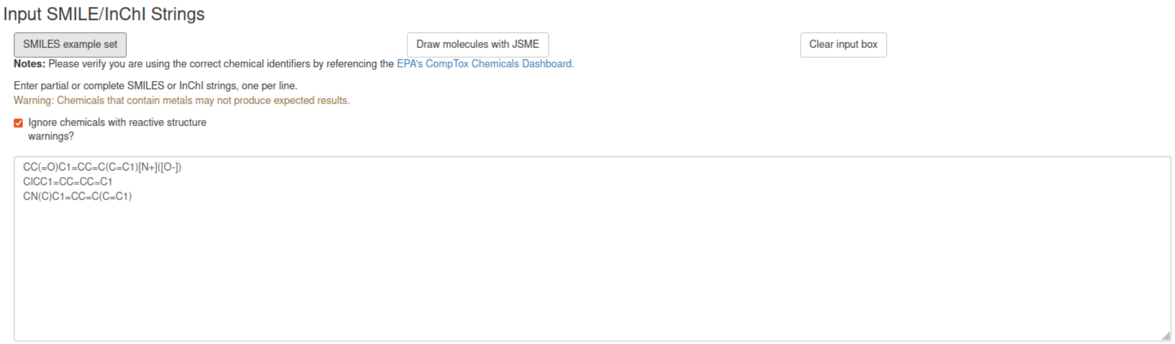

Clicking the **Draw molecules with JSME** button will open the JSME Molecule Editor by Peter Ertl and Bruno Bienfait (Bienfait and Ertl, 2013). Here, you can draw a molecule using the applet's tools. When you are finished drawing, we can right-click in the whitespace of the canvas and select "Copy as SMILES" or "Copy as InChI" and paste the copied text in the input box.

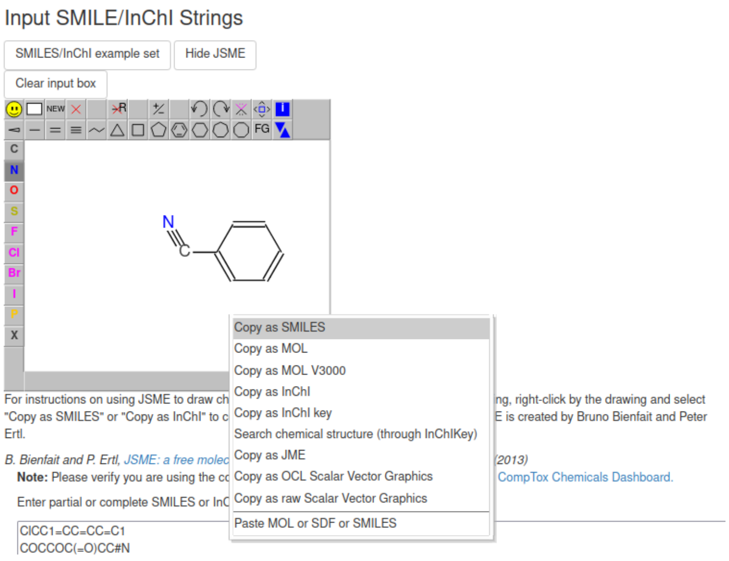

A submitted chemical may contain a certain reactive structure while some of its matching chemicals do not; alternatively, the matching chemicals may contain a reactive structure not present in the original chemical. Checking the **Ignore chemicals with reactive structure warnings** checkbox will cause Tox21Enricher-Shiny to ignore matching chemicals with this discrepancy and not include them in the enrichment process. **Note that inputting compounds that contain metals may not produce the expected results**.

II.3.c. Enrich From Chemicals With Structural Similarity

This enrichment analysis type accepts either SMILES or InChI strings (one per line). Each string is treated as its own data set and will be enriched independently of any other sets provided. SMILES and InChI strings may be mixed: you may perform enrichment with a SMILES string on one line and an InChI string on another. Clicking the **SMILES/InChI example set** button will fill the input box with three chemicals: two SMILES strings and one InChI string. Clicking the **Clear input box** button will reset the input box to a blank state. The **Tanimoto similarity threshold** may be specified using the slider controls above the input box to set the strictness of the search. By default, this is set to 50% and must be between 2% and 100% (inclusive).

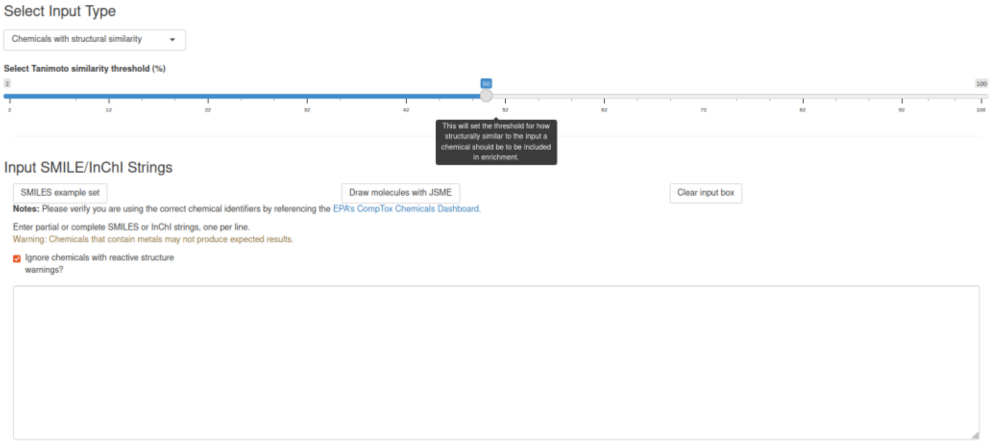

Clicking the **Draw molecules with JSME** button will open the JSME Molecule Editor by Peter Ertl and Bruno Bienfait (Bienfait and Ertl, 2013). Here, you can draw a molecule using the applet's tools. When you are finished drawing, we can right-click in the whitespace of the canvas and select "Copy as SMILES" or "Copy as InChI" and paste the copied text in the input box.

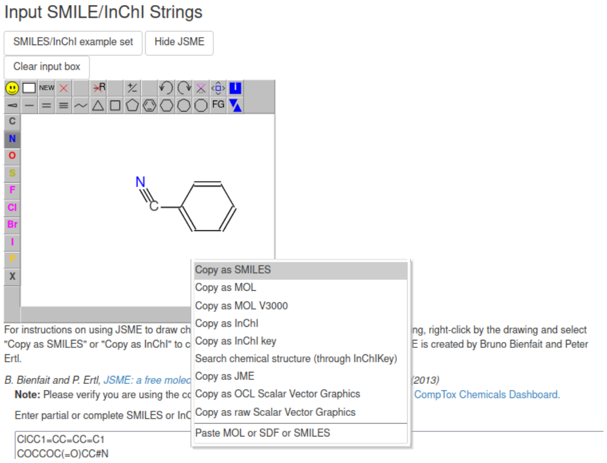

A submitted chemical may contain a certain reactive structure while some of its matching chemicals do not; alternatively, the matching chemicals may contain a reactive structure not present in the original chemical. Checking the **Ignore chemicals with reactive structure warnings** checkbox will cause Tox21Enricher-Shiny to ignore matching chemicals with this discrepancy and not include them in the enrichment process. **Note that inputting compounds that contain metals may not produce the expected results**.

II.3.d. View Annotations for Tox21 Chemicals

This function does not actually perform enrichment analysis; rather, it returns a list of every annotation in the Tox21 database that is associated with each of the supplied chemicals. Like the **User-provided CASRN list** enrichment type, this enrichment analysis type accepts only CASRN strings as input (one CASRN per line). You may find annotations for multiple sets of chemicals at the same time. To use multiple sets, separate lines of CASRN strings with **unique** set names of the form “#SetName” with a pound symbol (#) before the name. Only alphanumeric characters (A-Z, a-z and 0-9) may be used in set names. If you choose to provide names for input sets, **all** sets must be assigned a name. Spaces in set names or CASRN strings are ignored, and extra newline characters between, after, and before lines are also ignored. If no set names are provided, all lines of the input box will be treated as one set and will be assigned the default name of “Set1.”

This mode will not provide any annotations for a CASRN if that chemical is not present in the Tox21 screening library. To view all the chemicals in the Tox21 screening library, click the **chemicals in the Tox21 screening library** link which opens the page https://comptox.epa.gov/dashboard/chemical_lists/TOX21SL in a new window.

Clicking the **CASRNs example single set** button will fill the input box with one, unnamed set of CASRNs. Clicking the **CASRNs example multiple sets** button will fill the input box with three sets of CASRNs. Clicking the **Clear input box** button will reset the input box to a blank state.

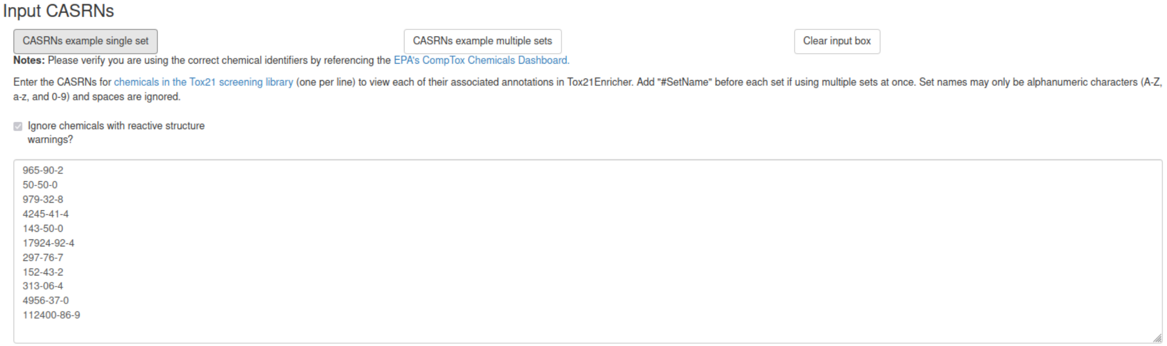

II.4. Selecting Enrichment Cutoff Value

Under the tabs for selecting the appropriate annotation categories, the landing page displays a slider input for specifying the enrichment cutoff value. This value determines the maximum number of enrichment results displayed per data set submitted. By extension, this will also determine the maximum number of nodes displayed during network generation. By default, this is set to 10 and can be increased to a maximum of 50 or decreased to a minimum of 1.

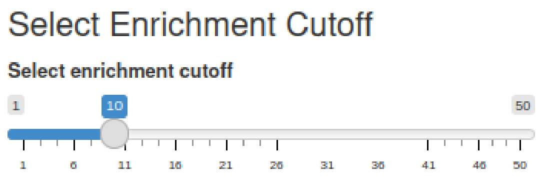

II.5. Selecting the P-Value Calculation Method

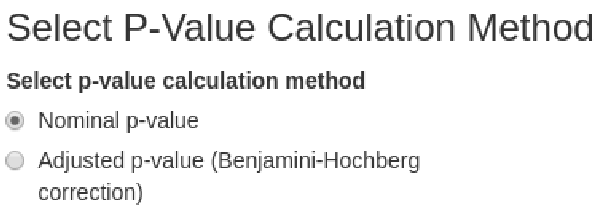

Tox21Enricher-Shiny calculates p-values for annotations during enrichment analysis to determine which terms are significantly overrepresented. Annotations with $p < 0.05$ are kept in the results while the others are discarded. Selecting “Nominal p-value” will cause the enrichment process to filter results using the raw p-value. Selecting “Adjusted p-value” will cause the enrichment process to filter results using each annotation’s p-value after being adjusted using Benjamini-Hochberg correction (Benjamini and Hochberg, 1995).

II.6. Performing Enrichment

After input has been entered, it is time to begin enrichment. This can be done by clicking on the **Submit** button under the enrichment cutoff slider. If there are no problems validating your input, you will be sent to the waiting page. Validation errors may occur if you input a string that is not a properly formatted CASRN, SMILES, or InChI identifier, if you forget to name a set, or if you try to submit a blank input. If a validation error occurs, Tox21Enricher-Shiny will display a warning and the request will not proceed. Note that your request may be rejected for a number of reasons. If your request is rejected, you may be asked to adjust your query or try again later. A request may be rejected if:

- You input nothing.
- None of your input SMILES or InChI strings match any chemicals in the database.
- The client application cannot connect to the Tox21Enricher-Shiny API.
- An input SMILES or InChI string matches an excessively large number of chemicals in the database. This number is variable and may be configured by the administrator of the remote host running the Tox21Enricher-Shiny API.

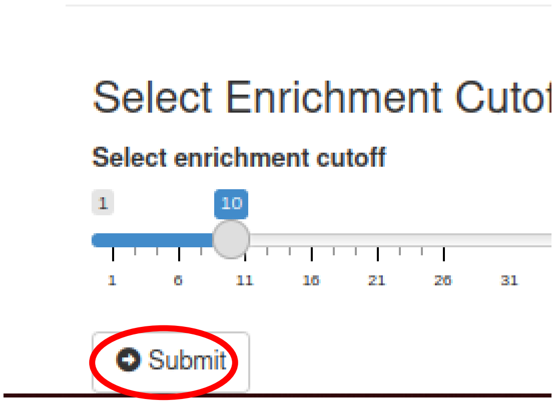

- You have input too many separate sets. The number of concurrent input sets is variable and may be configured by the administrator of the remote host running the Tox21Enricher-Shiny API.

III. Waiting Page

After submitting your request, you will be taken to the waiting page.

Your request has been submitted and placed in the queue. After your request is processed and completed, the results can be accessed by clicking the "Results" button. **Please make sure to save the request's UUID for future reference and access to the results.**

Copy UUID to clipboard | Cancel enrichment

Your Request

Show 10 entries | Search:

Status	Request Mode	Request UUID	Selected Annotations	Node Cutoff	User Input
Set2: (Step 1/4): Processing input file(s). Set3: (Step 1/4): Processing input file(s). Set1: (Step 1/4): Processing input file(s).	Enrich from chemicals with structural similarity	3034ba21-8cf8-45b6-84e2-69195ff3ecb9	MESH, PHARMACTIONLIST, ADVERSE_EFFECT, INDICATION, STRUCTURE_ACTIVITY, TISSUE_TOXICITY, TA_LEVEL_3, MECH_LEVEL_3, MECH_LEVEL_2, TA_LEVEL_2, THERAPEUTIC_CLASS, MECH_LEVEL_1, ACTIVITY_CLASS, MECHANISM, TA_LEVEL_1, MODE_CLASS, PRODUCT_CLASS, KNOWN_TOXICITY, DRUGBANK_TARGETS, DRUGBANK_ATC, DRUGBANK_ATC_CODE, DRUGBANK_ENZYMES, DRUGBANK_TRANSPORTERS, DRUGBANK_CARRIERS, CTD_CHEMICALS_DISEASES, CTD_CHEMICALS_GENES, CTD_CHEMICALS_PATHWAYS, CTD_CHEMICALS_GOENRICH_MOLFUNCT, CTD_CHEMICALS_GOENRICH_CELLCOMP, CTD_GOSLIM_BIOPROCESS, TOXINS_TARGETS, TOXCAST_ACTIVE, TOXPRINT_STRUCTURE, HTS_ACTIVE, HTS_STRONGACTIVE, LEADSCOPE_TOXICITY, MULTICASE_TOX_PREDICTION, TOXREFDB,	10	CC(=O)C1=CC=C(C=C1)[N+]([O-]) ClCC1=CC=CC=C1 CN(C)C1=CC=C(C=C1)

Showing 1 to 1 of 1 entries | Previous 1 Next

There are two buttons at the top of the waiting page that perform different tasks when clicked:

- **Copy UUID to clipboard** – This will copy your request's UUID to the clipboard so it may be pasted elsewhere.
- **Cancel enrichment** – This will open a prompt to cancel the request. Canceled requests cannot be recovered. The request's temporary file will be deleted from the client filesystem and records of the request will be deleted from the Tox21Enricher-Shiny database.

The waiting page will automatically update every few seconds to display the current status of each input set of the request. When the request is complete, you will automatically be taken to the results page.

Important: ***Please make sure to save your request's UUID somewhere where you may reference it later. Although every submitted request UUID is saved in a cookie in the browser, knowing the UUID for a given request is the only way to view that request's results after the cookie expires or is deleted.***

The **Your Request** table contains some helpful information related to the parameters of your request:

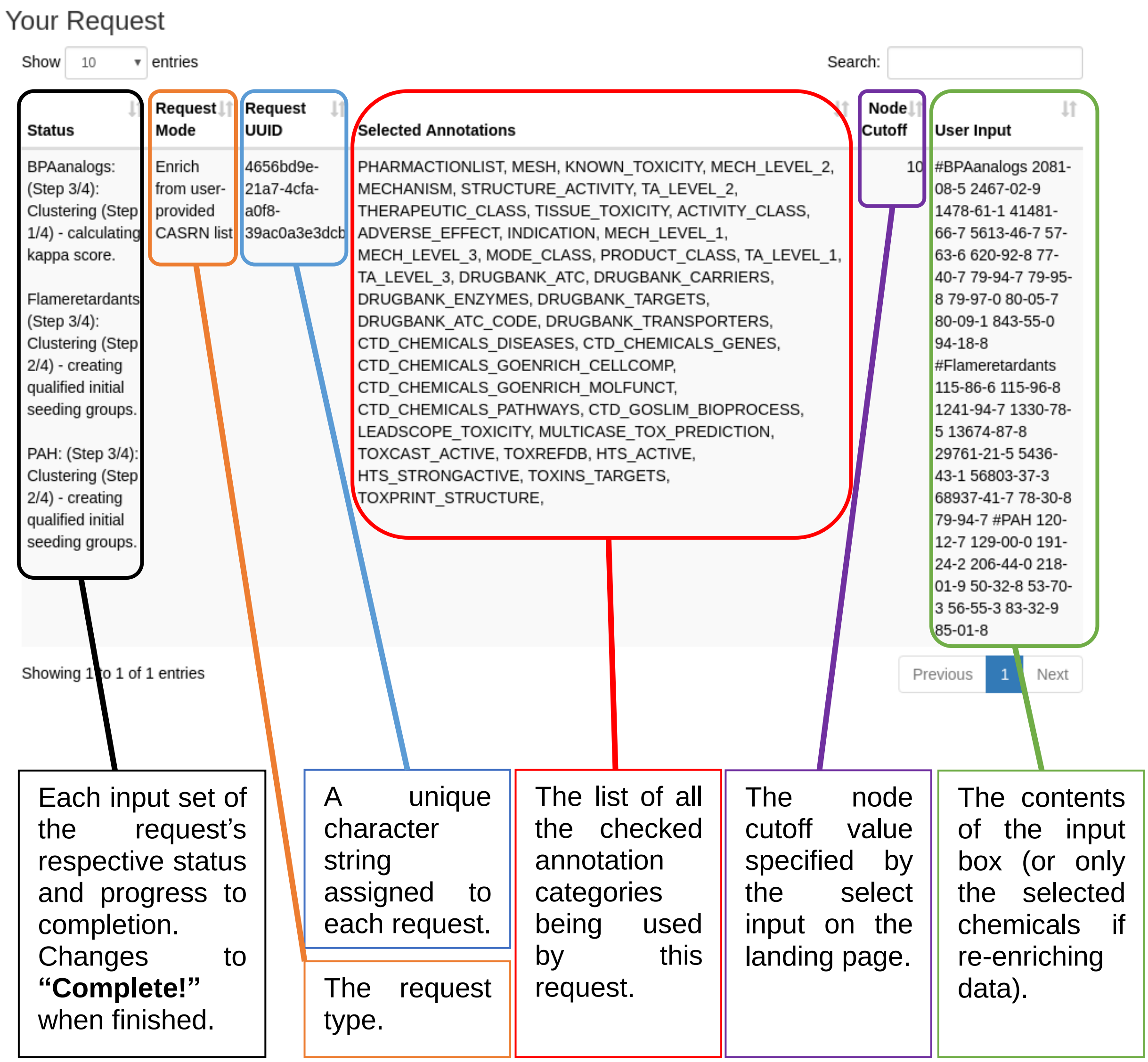

IV. Results Page

IV.1. Enrichment Results

Once your request has completed, you may click the **Results** button to view the request's results.

IV.1.a. Result Files

The top of the results page displays relevant files generated by the enrichment analysis process. If multiple sets were submitted, the files corresponding to each set are organized into separate tabs.

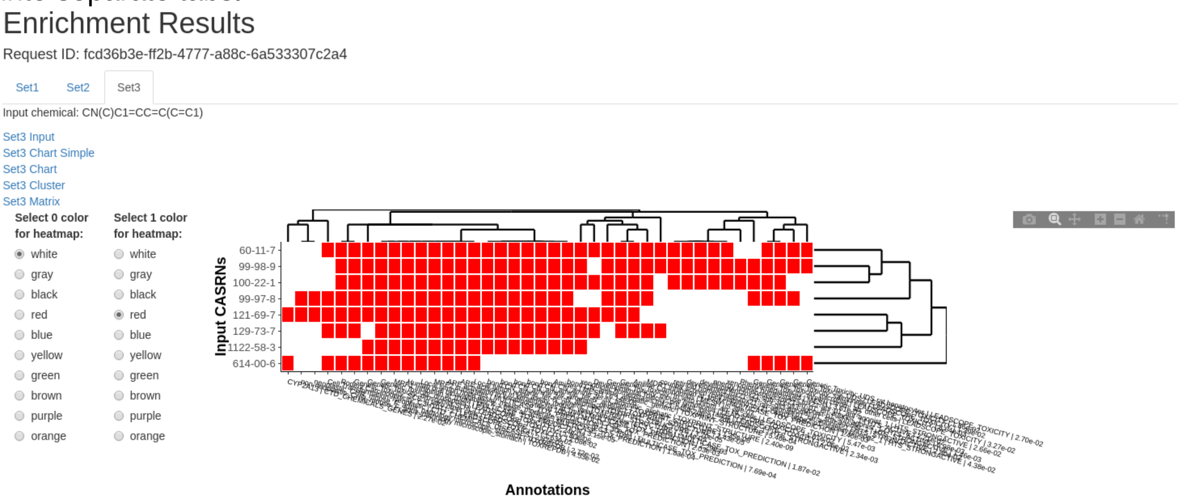

Additionally, if you submitted SMILES or InChI strings for enrichment, the original SMILES/InChI string is displayed with the set's results.

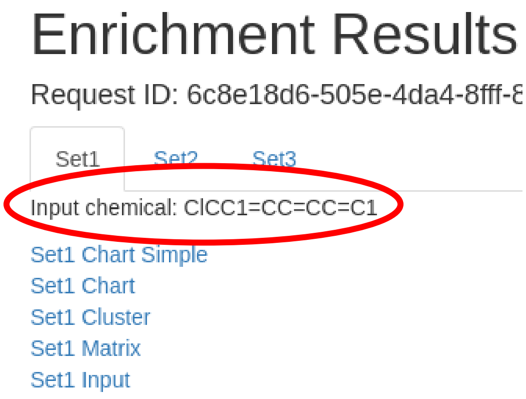

If one could be generated, an interactable heatmap using the Plotly library for R (https://plotly.com/r/) will be displayed under the result file links. A value of 1 signifies that the annotation on the x-axis belongs to the CASRN(s) on the y-axis, while a value of 0 signifies that the annotation is not associated with the CASRN(s) on the y-axis. The colors may be configured using the radio buttons input to the left. The cells in the heatmap are clustered by row and column with dendrograms.

Clicking any of the result file links will download a copy of the corresponding file from the Tox21Enricher-Shiny server to the client application's temporary cache so that it may be viewed in a table in the client application.

The result files are as follows:

- **<set_name> Chart Simple** – A simplified list of the top *N* most significantly overrepresented annotations for each annotation class, adjustable using the enrichment cutoff (.txt format).
- **<set_name> Chart** – A list of all significantly overrepresented annotations (.txt format).
- **<set_name> Cluster** – A list of significant terms in which functionally similar annotations are grouped together to remove redundancy. This is performed with respect to the whole annotation set rather than to individual annotation classes (.txt format).
- **<set_name> Matrix** – A plaintext representation of the heatmap (.txt format).
- **<set_name> Input** – A plaintext list of the input chemicals for this set (.txt format).
- **<set_name> Error CASRNS** – A list of any submitted or matched CASRNs that were not able to be matched with any annotations.

Chart file for Set1 Close preview

Copy CSV Excel PDF Print

Search:

Category	Term	Count	%	PValue	CASRNs	List Total	Pop Hits
TOXPRINT_STRUCTURE	bond:CC(=O)C_ketone_aliphatic_acyclic	3	100.00	2.1e-3	1009-61-6, 99-93-4, 122-00-9	3	38[illegible]
TOXPRINT_STRUCTURE	bond:CC(=O)C_ketone_aromatic_aliphatic	3	100.00	2.4e-3	122-00-9, 99-93-4, 1009-61-6	3	40[illegible]

IV.1.b. Result Chemicals

The **Result Chemicals** table is only visible when enriching from chemicals with shared substructures or structural similarity. This table displays information about all of the related chemicals to the original SMILES/InChI input strings.

Result Chemicals

Copy CSV Excel PDF Print Column visibility

Search:

Select	Chemical Structure	DSSTox Substance ID	CASRN	IUPAC Name	SMILES	InChI	InChI Key	Molecular Formula	Molecular Weight	Similarity
✔	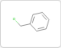	DTXSID0020153	100-44-7	chloromethylbenzene	ClCC1=CC=CC=C1	InChI=1S/C7H7Cl/c8-6-7-4-2-1-3-5-7/h1-5H,6H2	KCXMKQUNVWSEMD-UHFFFAOYSA-N	C7H7Cl	126.58	1
✔	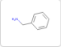	DTXSID5021839	100-46-9	phenylmethanamine	NCC1=CC=CC=C1	InChI=1S/C7H9N/c8-6-7-4-2-1-3-5-7/h1-5H,6,8H2	WGQKYBSKWIADBV-UHFFFAOYSA-N	C7H9N	107.15	0.56

Additionally, if you performed enrichment from chemicals with structural similarity, an additional column is shown displaying a Tanimoto value describing the similarity of each result chemical to the original input chemical. Clicking the chemical structure image for a given chemical will open a modal that displays a larger image of the chemical structure, additional information about the chemical, and links to view the chemical's entries on the EPA's CompTox Chemicals Dashboard website and the PubChem database.

100-44-7 Close

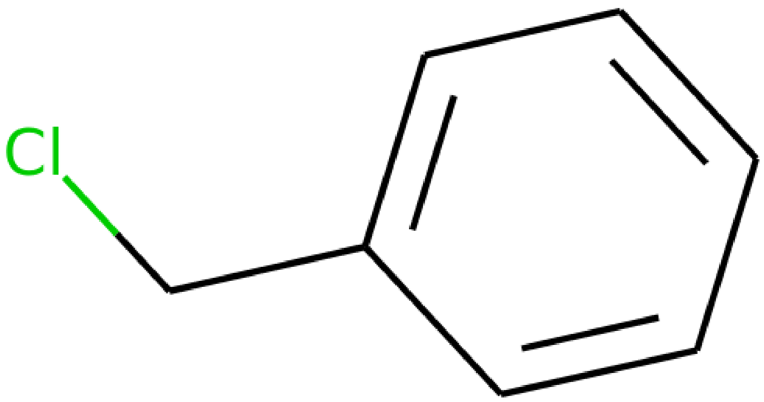

**DTXSID**	DTXSID0020153
**DTXRID**	75405
**Chemical Name**	Benzyl chloride
**IUPAC Name**	chloromethylbenzene
**CASRN**	100-44-7
**SMILES**	ClCC1=CC=CC=C1
**InChI**	InChI=1S/C7H7Cl/c8-6-7-4-2-1-3-5-7/h1-5H,6H2
**InChI Key**	KCXMKQUNVWSEMD-UHFFFAOYSA-N
**Molecular Formula**	C7H7Cl
**Molecular Weight**	126.58
View at EPA	View at PubChem

Close

The menu buttons above the table perform additional functions:

- **Copy** – Copies the entire table to the clipboard.
- **CSV** – Allows you to save a copy of the table as a .csv file.
- **Excel** – Allows you to save a copy of the table as an Excel spreadsheet.
- **PDF** – Allows you to save a copy of the table as a .pdf file.
- **Print** – Allows you to print a copy of the table.
- **Column visibility** – Allows you to selectively show or hide certain columns of the table.

Each chemical has an associated checkbox that marks the chemical for re-enrichment. By default, all chemicals' checkboxes are checked.

If an originally submitted SMILES/InChI string contains a certain reactive structure, Tox21Enricher-Shiny will check to see if each of its related chemicals also contain that structure. If a related chemical does not contain the structure, an additional **Reactive Structure Warning** column is added to the **Result Chemicals** table displaying a picture of the suspect structure. A warning will also be displayed if the original input string does not contain a reactive structure, but one of its related chemicals does. Currently, Tox21Enricher-Shiny displays warnings if the following structural groups are detected in either (but not both) the input chemical and one of its related chemicals:

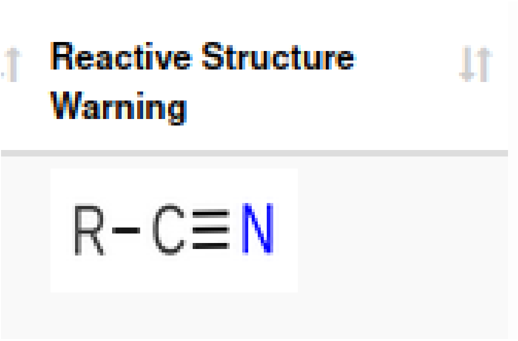

- **Nitrile group (cyanide)**
- **Aldehyde**
- **Epoxide**
- **Isocyanate**

IV.1.c. Download Results Button

Clicking the **Download results** button will prompt you to download a .zip archive of all of the results files for all the sets for a given request.

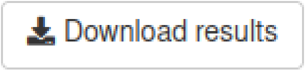

IV.1.d. Adjust Network Node Cutoff & Perform Re-enrichment

Below the result files and chemicals is a series of controls for rerunning enrichment on the result set. First is a slider control for adjusting the node cutoff value so that you may change the number of nodes without having to completely perform enrichment again from scratch.

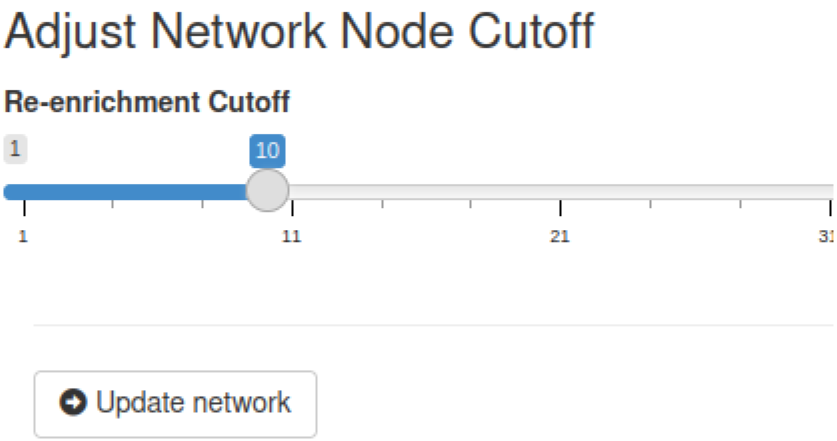

Under this slider input, there is a button to either update the network (if enriching from a user-provided CASRN list) or perform enrichment again with only the selected result chemicals (if enriching from chemicals with structural similarity or shared substructures). Additionally, if enriching from chemicals with structural similarity or shared substructures, more buttons are present that interact with the **Result Chemicals** table:

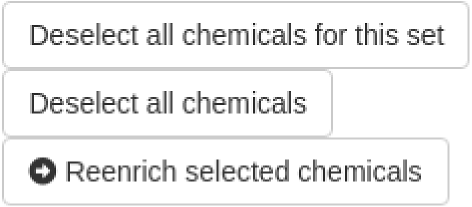

- **Deselect all chemicals for this set** – Deselects all of the result chemicals only for the currently selected tab (result set).
- **Deselect all chemicals** – Deselects all of the result chemicals across all tabs.

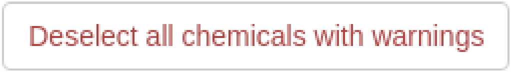

Additionally, if any result sets have chemicals with reactive structure warnings, a third **Deselect all chemicals with warnings** button will appear. Clicking this button will deselect all chemicals with reactive structure warnings across all tabs.

IV.1.e. Chart and Cluster Heatmaps

Below the re-enrichment and node cutoff controls are interactive heatmaps using the Plotly library for R. The chart heatmap displays the most significant annotations across all the chemicals in each set.The colors can be configured using the radio buttons input to the left

Chart Full Heatmap

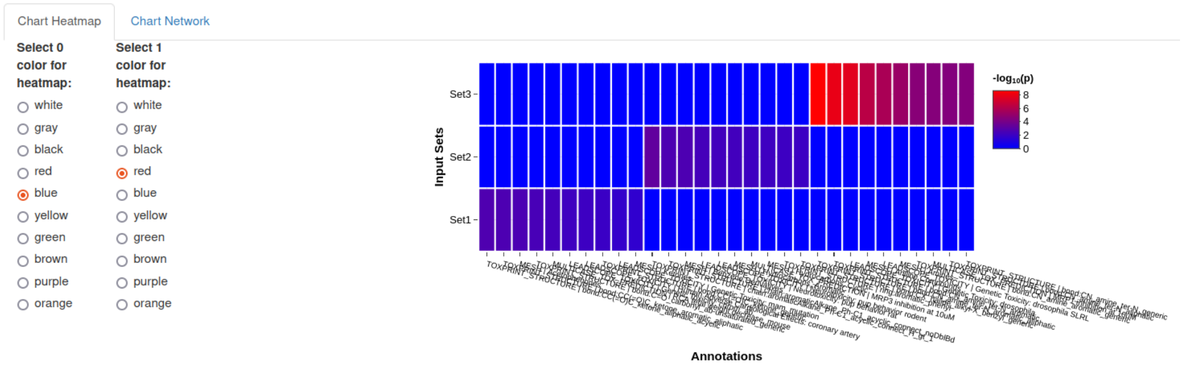

The cluster heatmap displays the most significant annotations across all the chemicals in each set as well, but reduces these annotations down to groups of functionally similar annotations. The colors can be configured using the radio buttons input to the left:

Cluster Full Heatmap

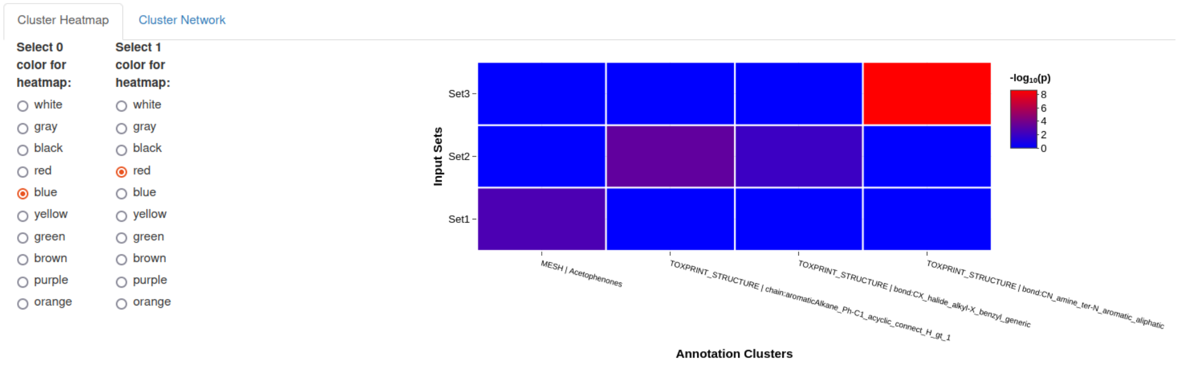

IV.1.f. Chart and Cluster Networks

Clicking on the **Chart Network** tab next to the **Chart Heatmap** tab will open the chart network display. Similarly, clicking on the **Cluster Network** tab next to the **Cluster Heatmap** tab will open the cluster network display. Both network displays have identical layouts. The nodes correspond to significantly enriched annotations and the edges indicate that there is significant overlap between the two annotations in terms of chemical contents. The edge color gradient indicates the degree of overlap based on a Jaccard index. Note that the network may take some time to load depending on the number of nodes represented.

On the top-left side, there is a number input for changing the q-value. This is the cutoff value for the statistically significant overlap between pairs of nodes. You may input a -$\log_{10}$-transformed q-value (for convenience), and the application will display the corresponding raw q-value next to the input. Clicking the **View q-value distribution** link under the input will open a modal that shows the q-value distribution as a table and a bar plot for the pairs in the current network. There are also checkboxes for changing which input sets and/or annotation classes should be represented in the network. Under the **Other Options** section, there is the option to enable network physics, which will make the nodes physically react with each other. There is also the option to toggle curve smoothing for the network edges. This is enabled by default, but disabling it may improve performance. Clicking the **Update network** button will regenerate the network, reflecting any changes made to selected input sets, physics, or the q-value.

In the middle of the network display, there are two drop-down menus. The top menu allows you to select certain annotations in the network without having to click on the nodes in the network. The bottom menu allows you to hide all annotations not of the selected category, making the network easier to navigate and read. Below these menus is a legend that shows each category’s unique color. You may interact with the network by clicking and dragging nodes around the canvas. The mouse wheel can be used to zoom in and out of the network view as well. Additionally, the green buttons overlaid on the network may also be clicked to manipulate the network view. Under the network, there is the **Export as png** button which allows you to save an image of the network as a .png file. Under the **More Information for Selected Annotation** section, you will see a prompt to select any node in the network. Doing this will display a link to an external website or database with more details about the annotation in the selected node.

Under the **Overlapping Chemicals** section, you will see a prompt to select any edge in the network. Doing this will generate a Venn diagram under the network detailing the number of chemicals shared and unique to the annotations displayed in the connecting nodes of the selected edge. Clicking an edge will also generate buttons that will allow you to view a list of all the chemicals (as CASRNs) associated with either of the two given annotations. A third button will also be generated that will allow you to view a list of all the chemicals (as CASRNs) that are associated with both given annotations. Underneath these buttons are links that allow you to save the Venn diagram as a .png image or .pdf document when clicked.

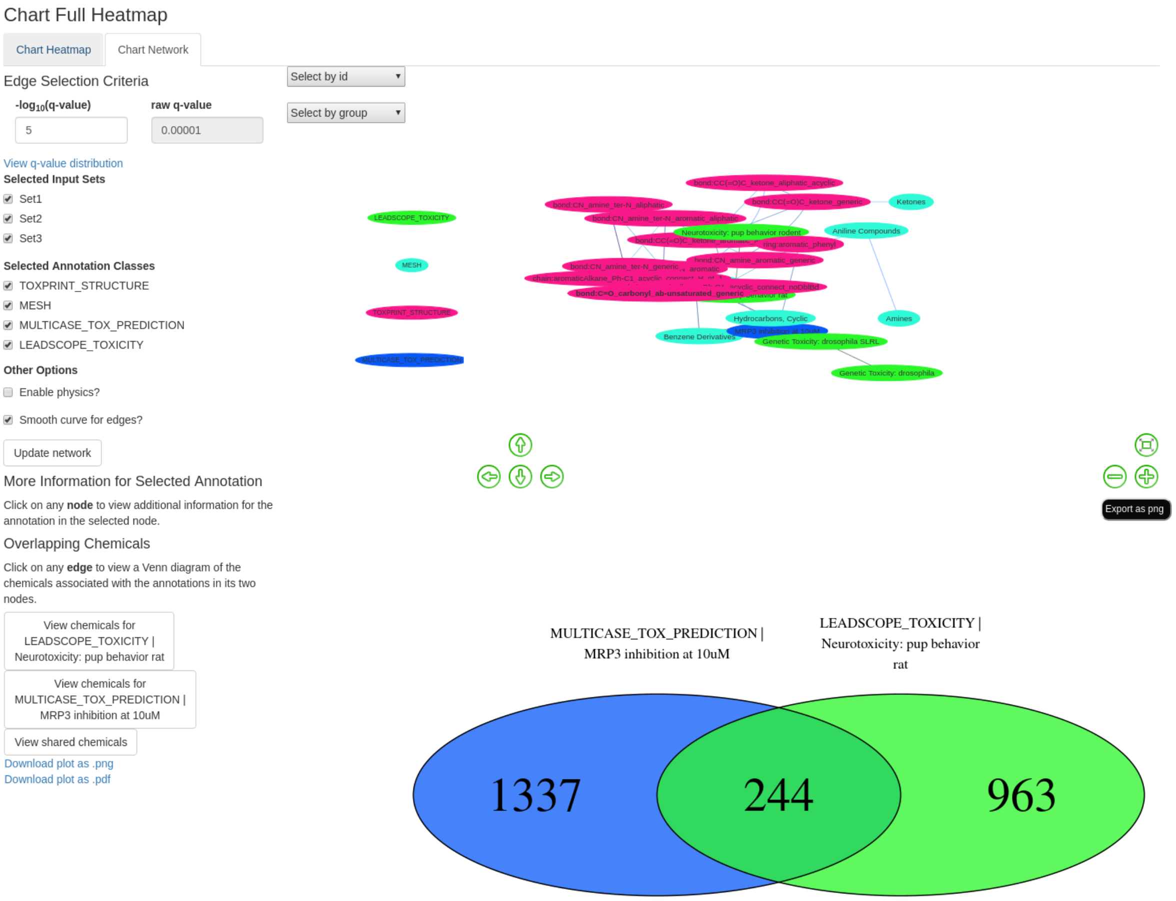
Chart Full Heatmap
Chart Heatmap
Chart Network
Edge Selection Criteria
-log10(q-value)
raw q-value
5
0.00001
View q-value distribution
Selected Input Sets
Set1
Set2
Set3
Selected Annotation Classes
TOXPRINT_STRUCTURE
MESH
MULTICASE_TOX_PREDICTION
LEADSCOPE_TOXICITY
Other Options
Enable physics?
Smooth curve for edges?
Update network
More Information for Selected Annotation
Click on any node to view additional information for the annotation in the selected node.
Overlapping Chemicals
Click on any edge to view a Venn diagram of the chemicals associated with the annotations in its two nodes.
View chemicals for LEADSCOPE_TOXICITY | Neurotoxicity: pup behavior rat
View chemicals for MULTICASE_TOX_PREDICTION | MRP3 inhibition at 10uM
View shared chemicals
Download plot as .png
Download plot as .pdf
Select by id
Select by group
Ketones
Aniline Compounds
Amines
Hydrocarbons, Cyclic
Benzene Derivatives
Neurotoxicity: pup behavior rodent
Genetic Toxicity: drosophila SLRL
Genetic Toxicity: drosophila
Export as png
MULTICASE_TOX_PREDICTION | MRP3 inhibition at 10uM
LEADSCOPE_TOXICITY | Neurotoxicity: pup behavior rat
1337
244
963

IV.1.g. Significant P-value per Annotation Category Bar Graphs

At the bottom of the results page, you can find a series of bar graphs that display all the significant annotations across all result sets and each set's inverse p-value for each annotation, if applicable. Separate bar graphs are created for each annotation category and are put into each category's respective tab. A color legend appears on the righthand side of each bar graph if multiple sets are represented. Clicking on a set name in this legend will hide its trace from the graph, while double-clicking a set name will hide all other sets' traces. To the left of the bar graphs is a menu that allows you to select one set to order values by. Clicking the **Update plot** button will update all the bar graphs to be ordered with respect to the selected set. Trace colors are unique for each set and are randomly selected from a predefined list each time enrichment is performed. Bar graphs are always ordered with the most significant value for the selected set at the top and the least significant value for the selected set at the bottom. The data in each bar graph are also represented in a table below each bar graph. Each table may be saved in different formats by using the buttons above each table.

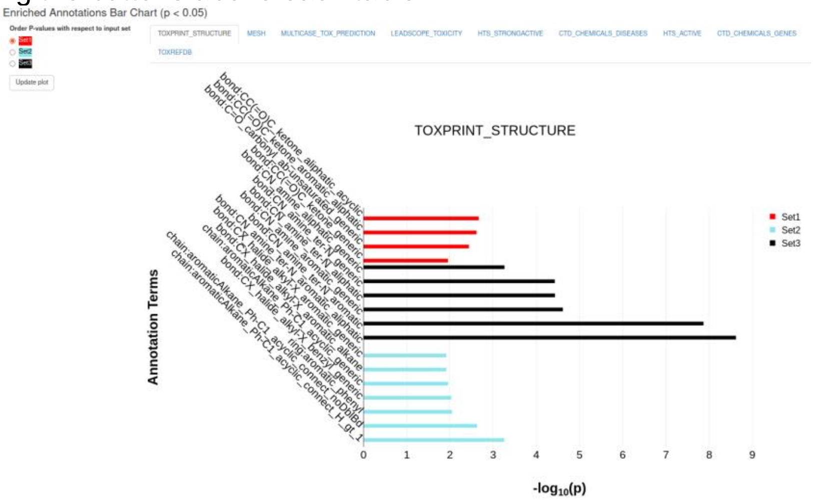

$-log_{10}(p)$ for TOXPRINT_STRUCTURE

Annotation	Set1	Set2	Set3
bond:CC(=O)C_ketone_aliphatic_acyclic	2.67	0.00	0.00
bond:CC(=O)C_ketone_aromatic_aliphatic	2.62	0.00	0.00
bond:C=O_carbonyl_ab-unsaturated_generic	2.44	0.00	0.00
bond:CC(=O)C_ketone_generic	1.96	0.00	0.00
chain:aromaticAlkane_Ph-C1_acyclic_connect_H_gt_1	0.00	3.26	0.00
chain:aromaticAlkane_Ph-C1_acyclic_connect_noDblBd	0.00	2.63	0.00
ring:aromatic_phenyl	0.00	2.05	0.00
bond:CX_halide_alkyl-X_benzyl_generic	0.00	2.03	0.00

IV.1.h. Start Over Button

After enrichment is successfully performed, the **Start over** button is added to the bottom of the sidebar. Clicking this button will take you back to the landing page. This button is functionally identical to refreshing the application from within an internet browser.

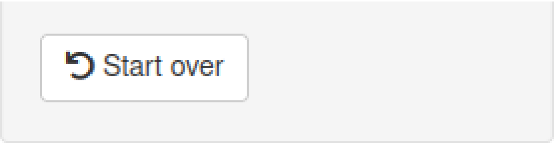

IV.2. Annotation Results

IV.2.a. Result Files

The top of the results page displays relevant files generated when fetching annotations. If multiple sets were submitted, the files corresponding to each set are organized into separate tabs.

Each submitted CASRN will have its own text file with a name of the form **<casrn>**. This file will contain all of the annotations in the Tox21Enricher-Shiny database that are associated with the submitted chemical.

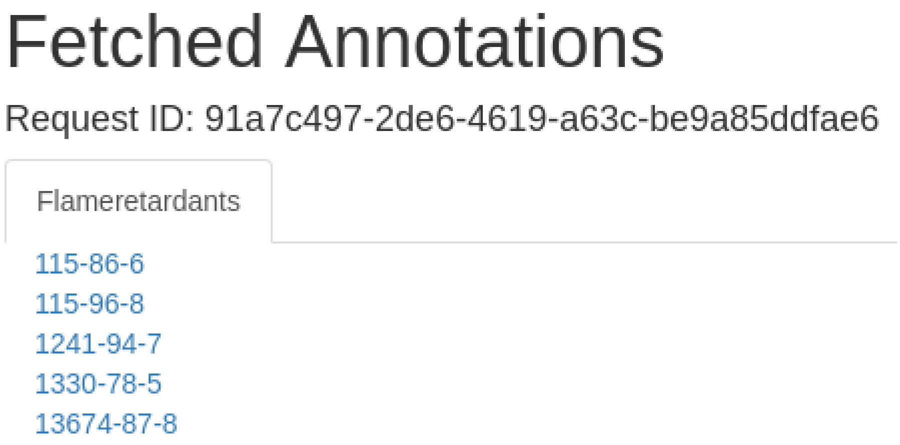

The annotations in this file are organized so that each line contains one complete annotation: the category the annotation belongs to is on the left and the annotation's name is on the right, and they are separated by a tab. If Tox21Enricher-Shiny could not find any associated annotations for a submitted CASRN, the CASRN will be ignored and a corresponding result file will not be generated. The annotation-fetching process will only search through the annotation categories that were checked on the landing page.

An additional text file of the **name <set_name> Full Matrix** will be generated for each submitted set of CASRNs. This file contains a plaintext representation of a matrix of all the fetched annotations in the set and if they are associated with each of the submitted CASRNs. An interactive and configurable heatmap for this matrix will also be displayed.

Much like when performing enrichment, The input file will be displayed here with the name **<set_name> Input**. If any CASRNs produced errors or did not match any annotations in the database, a file will be produced called **<set_name> Error CASRNs** listing the problem chemicals.

IV.2.b. Download Results Button

Clicking the **Download results** button will prompt you to download a .zip archive of all of the results files for all the sets for a given request.

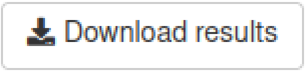

V. Viewing Results From Previous Requests

Clicking the **View previous results** button on the sidebar will take you to the **View Results from Previous Request** page.

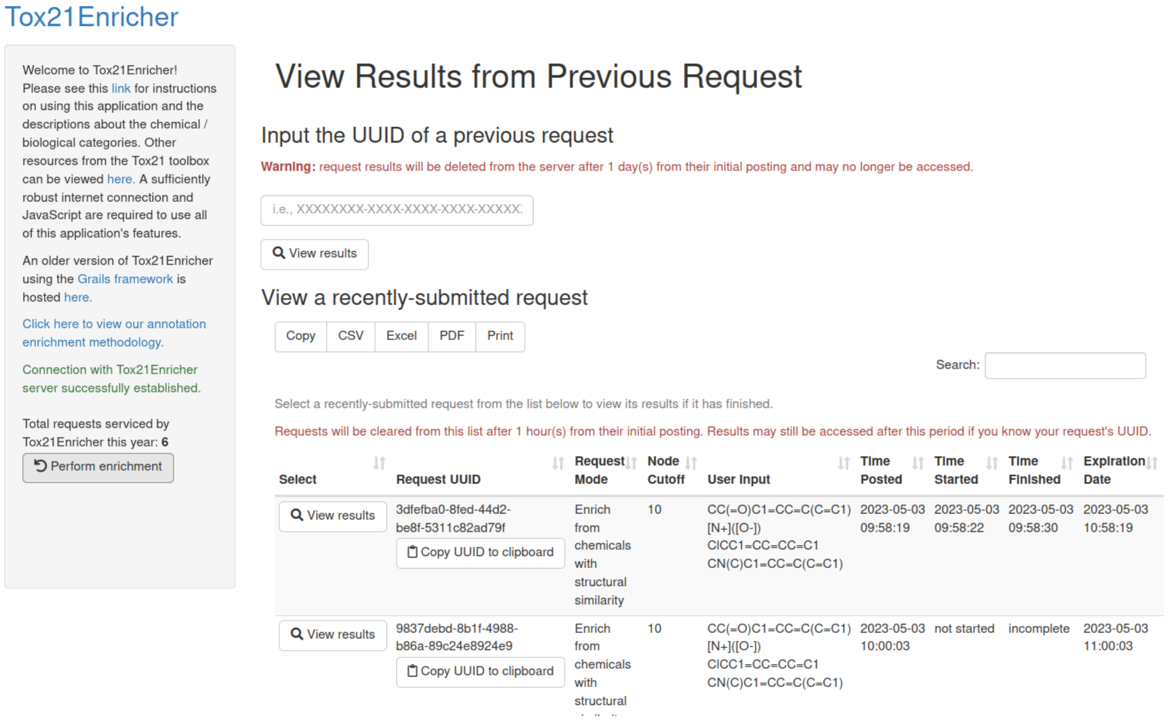

You may input the UUID of a previous request in the text input box, labeled **Input the UUID of a previous request**. You can then select the **View results** button. If the request exists and has completed, you will be redirected to the results page of the request.

Please note that records for all submitted requests will be deleted after a certain number of days have passed since the request was posted. The exact number of days may be configured by the host of the Tox21Enricher-Shiny API but otherwise requests will be deleted after a default of 30 days. After a request's records are deleted, you may no longer use this page to view its result files. The request's entry will remain in the Tox21Enricher-Shiny database indefinitely until it is deleted by the database's administrator.

When a request is submitted, a cookie called **previous_session_id__<uuid>**, where <uuid> is the UUID of the submitted request, will be saved to your browser. This cookie's value will be set to the UUID of the submitted request. The cookie's expiry time may be set in the configuration file of the API on the host but otherwise has a default value of 48 hours or 2 days from the time the request was submitted (not when the request was first processed by the queue). If the client application detects that one or more non-expired cookies exist, this page will display information and a "View results" button for each cookie's corresponding request under the **View a recently-submitted request** section.

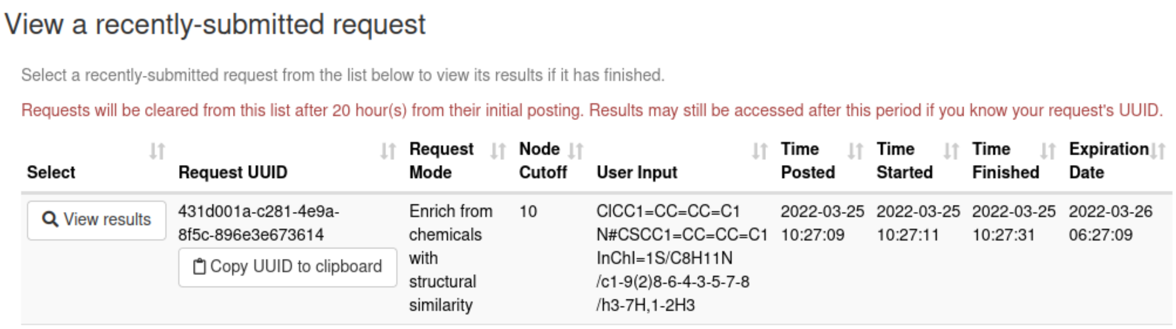

View a recently-submitted request

Select a recently-submitted request from the list below to view its results if it has finished.

Requests will be cleared from this list after 20 hour(s) from their initial posting. Results may still be accessed after this period if you know your request's UUID.

Select	Request UUID	Request Mode	Node Cutoff	User Input	Time Posted	Time Started	Time Finished	Expiration Date
View results	431d001a-c281-4e9a-8f5c-896e3e673614 Copy UUID to clipboard	Enrich from chemicals with structural similarity	10	ClCC1=CC=CC=C1 N#CSCC1=CC=CC=C1 InChI=1S/C8H11N /c1-9(2)8-6-4-3-5-7-8 /h3-7H,1-2H3	2022-03-25 10:27:09	2022-03-25 10:27:11	2022-03-25 10:27:31	2022-03-26 06:27:09

Additionally, if the client application detects that one or more non-expired cookies exist, it will display this blue notification in the bottom-right corner of the screen. Clicking the “here” link will take you back to the **View Results from Previous Request** page, where you may then proceed to view the request’s results if it has finished.

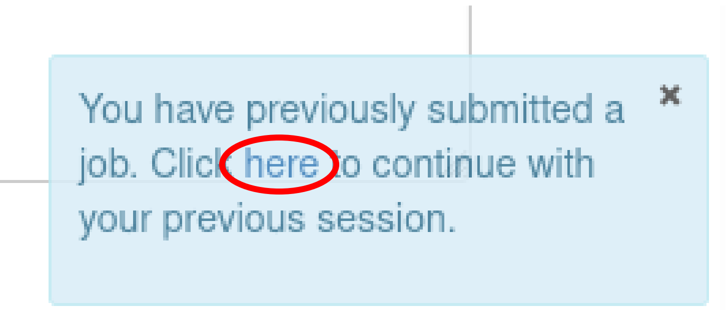

If you cancel a submitted request from the **Waiting Page**, its cookie will be deleted from your browser and the request will not show up when viewing previous requests.

Please note that in order to take full advantage of all the features listed in this section, you must allow cookies in your browser when using the Tox21Enricher-Shiny client application.

VI. Headless Mode

A user may make direct calls to the Tox21Enricher-Shiny API to perform enrichment, fetch annotations, and download results without using the client application's graphical user interface (GUI). The recommended tool for accessing the API from the command line is **cURL**. To make a request, use the cURL command:

```
curl --header "Content-Type: application/json" --request POST -data
'{"mode":"<mode>", "input":"<input_string>",
"annotations":"<annotation_class_list>", "cutoff":<cutoff>,
"tanimoto":<tanimoto>}' http://<server_address>:<port_api_is_running_on>/submit
```

Where:

- **<mode>** is the type of request (casrn, substructure, similarity, or annotation)
- **<input_string>** is a newline (\n)-separated string of input chemical identifiers. If inputting multiple sets of CASRNs, also include set names with a pound symbol (#) in front of each name.
    - Make sure you are submitting the correct identifier for the type of request you are making (i.e., CASRNs for "casrn" and "annotation" and SMILES or InChI strings for "substructure" and "similarity"). Also, if submitting CASRNs, make sure you are using the correct chemical identifiers as included in the EPA's CompTox Chemicals Dashboard: https://comptox.epa.gov/dashboard.
    - For example, for the mode "casrn," you could input something like: `#Set1\n965-90-2\n50-50-0\n#Set2\n4245-41-4\n143-50-0\n17924-92-4`
    - For example, for the mode "similarity," you could input something like: `ClCC1=CC=CC=C1\nN#CSCC1=CC=CC=C1`
- **<annotation_class_list>** is a comma-separated string of annotation class names. This denotes which annotation classes should be included when matching annotations to the input.
    - For example, you could input something like: `MESH,PHARMACTIONLIST,ACTIVITY_CLASS,ADVERSE_EFFECT,INDICATION`
    - You may run the command `curl http://<server_address>:<port>/annotationList` to view a list of all available annotation classes.
- **<cutoff>** is the integer value that determines the maximum number of annotations per annotation class to include in things like the network and ChartSimple files.
    - This must be an integer between 1 and 50 inclusive.
    - If not supplied, this will default to 10.
- **<tanimoto>** is the float value that determines the Tanimoto threshold. If a chemical's similarity score is greater than this value, it will be excluded from enrichment analysis.
    - This is only used with the "similarity" mode and will be ignored if supplied with another mode.
    - If not supplied, this will default to 0.50. This must be between 0.02 and 1.00 (inclusive).

Once a request is made, it will be placed in the queue like any other request made from the client application. If the request was successfully made, cURL will return the newly assigned UUID of the request in the terminal.

To check the status of the request, you may run the cURL command:
`curl http://<server>:<port>/isComplete?<uuid>`. This function will either return a 0 if the request has not completed or a 1 if the request has completed.

To download the results once the request is completed, you may run the cURL command:
`curl http://<server>:<port>/download?<uuid> > <name_of_downloaded_file>.zip`.
This will download a zipped folder containing all the request's results to the file <name_of_downloaded_file>.zip in the current working directory. This request will fail if the request has not yet completed. You may also use this command to download the results of any request, even those that were initially submitted using the client application's GUI.

To view a list of all annotation classes currently in the Tox21Enricher-Shiny database, you may run the cURL command `curl http://<server>:<port>/getAnnotationList`.

Examples for using Tox21Enricher-Shiny's headless mode can be found in the project's GitHub repository (https://github.com/hurlab/tox21enricher) at `<project_root>/demos/`. You may need to run the command `sed -i -e 's/\r$//'` on the bash scripts in this folder if you have first cloned the repository to a Windows machine, as Windows will insert different newline characters into the scripts.

VII. Common Issues & Questions

Both the client and server code for Tox21Enricher-Shiny are still in continued development, so there are likely issues that may arise when trying to either perform certain tasks or set up the application. Known bugs in the code and planned features are documented on the project's GitHub repository at: https://github.com/hurlab/tox21enricher/issues. Other known issues not necessarily related to the code itself are documented in this section of the manual.

Q: I am trying to run a structural similarity search, but Tox21Enricher-Shiny gives me the error, “no valid input sets.”

A: Try relaxing the Tanimoto similarity threshold. Thresholds that are too strict may not match many, if any, chemicals in Tox21.

VIII. Resources

Below are a few resources that users may find helpful:

Name	Link	Description
CompTox Chemicals Dashboard	https://comptox.epa.gov/dashboard	Reference for chemical identifiers to be used in Tox21Enricher-Shiny.
Hurlab Server	http://hurlab.med.und.edu/tox21enricher/	Location of Tox21Enricher-Shiny on the Hurlab web server.
Old Tox21Enricher-Shiny	http://hurlab.med.und.edu/tox21enricher-grails/	Location of the old Tox21Enricher-Shiny using Grails on the Hurlab web server.
RDKit	https://www.rdkit.org/docs/index.html	Documentation for the RDKit cheminformatics tool.
Tox21Enricher-Shiny GitHub Repository	https://github.com/hurlab/tox21enricher	Tox21Enricher-Shiny public GitHub repository.
Toxicology in the 21st Century	https://tox21.gov/	Information regarding the Tox21 data set.

IX. References

B. Bienfait and P. Ertl, JSME: a free molecule editor in JavaScript, J. Cheminformatics 5:24 (2013).

Y. Benjamini and Y. Hochberg, Controlling the False Discovery Rate: A Practical and Powerful Approach to Multiple Testing, Journal of the Royal Statistical Society: Series B (Methodological) 57:1 (1995) pp. 289-300. doi: 10.1111/j.2517-6161.1995.tb02031.x.

END OF THE USER MANUAL
